# Bypassing the lack of reactivity of *endo*-substituted norbornenes with the catalytic rectification–insertion mechanism†
†Electronic supplementary information (ESI): Experimental procedures, NMR characterization, kinetic plots, ORTEP diagrams and cif files. CCDC 1034345–1034348 and 1034422. For ESI and crystallographic data in CIF or other electronic format see DOI: 10.1039/c4sc03575e


**DOI:** 10.1039/c4sc03575e

**Published:** 2014-12-24

**Authors:** Basile Commarieu, Jerome P. Claverie

**Affiliations:** a Quebec Center for Functional Materials , UQAM, Dept of Chemistry , Succ Centre Ville CP8888 , Montreal H3C3P8 , Qc , Canada . Email: claverie.jerome@uqam.ca

## Abstract

The catalytic 1,2-insertion polymerization of polar norbornenes (NBEs) leads to the formation of functional rigid macromolecules with exceptional thermal, optical and mechanical properties. However, this remarkable reaction is plagued by the low reactivity of the polar monomers, and most notably of those bearing a functional group in *endo* position. We have examined the polymerization mechanism of NBEs bearing one or two CO_2_Me groups either in *exo* or *endo* position catalyzed by the so-called naked allyl Pd^+^ SbF_6_^–^ catalyst (**1**). Although *endo* dimethyl ester of 5-norbornene-2,3-dicarboxylic acid (NBE(CO_2_Me)_2_) is polymerized by **1**, two *endo* units are never inserted consecutively along the polymer chain. Indeed, **1** is a tandem catalyst which not only catalyzes the insertion of the monomer but also the isomerization of *endo* and *exo* isomers. Thus, the polymerization of *endo* monomers proceeds *via* a novel mechanism, coined rectification–insertion mechanism, whereby half of the *endo* monomers are rectified into *exo* ones prior insertion, leading to the formation of an alternating *endo*–*exo* copolymer using an *endo* only feedstock. With this mechanism, the lack of reactivity of *endo* norbornenes is bypassed, and the polymerization of predominantly *endo* polar NBEs bearing a variety of functionalities such as esters, imides, acids, aldehydes, alcohols, anhydrides, or alkyl bromides proceeds with catalyst loadings as low as 0.002 mol%.

## Abstract 2

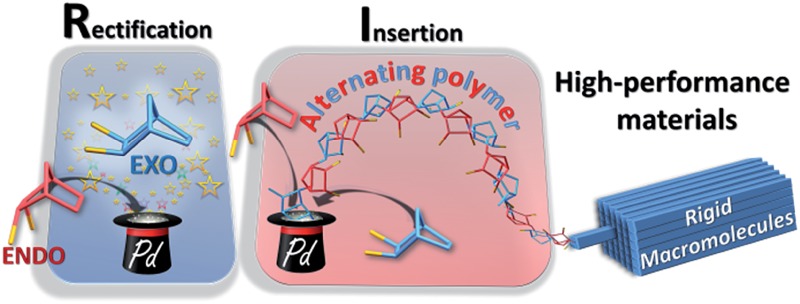
The novel rectification–insertion mechanism for the polymerization of polar norbornenes: making alternating copolymers from a single monomer.

## Introduction

Functional macromolecules are essential components for the formation of complex nanostructures with defined shape and functionality with applications in optical and electronic materials, catalysis, recognition/separation technologies and drug delivery.^1^ Among those, polynorbornene (PNBE) prepared by 1,2-insertion catalytic polymerization is particularly interesting as it has excellent thermal and chemical stability (degradation above 400 °C), high Tg (>300 °C), low dielectric constant and low birefringence.^2^,^3^ Furthermore, when disyndiotactic, this rigid polymer adopts a distinctive tubular helical molecular crystalline structure.^4^ Polar norbornenes (NBEs) can be prepared from straightforward Diels–Alder reactions between cyclopentadiene and simple dienophiles. Thus, one envisions that the polymerization of polar NBEs could become a remarkably efficient and green route to prepare functional polymers which could eventually rival in practicality with controlled radical polymerization,^5^–^7^ group-transfer polymerization^8^,^9^ or metathesis-based polymerizations.^10^–^14^ However, despite the existence of numerous NBE polymerization catalysts based on either early transition metals such as Zr or V or late transition metals such as Pd or Ni,^15^–^18^ the polymerization of polar NBEs is still plagued by several ineluctable issues. First, and probably most importantly, NBEs bearing *endo* polar substituents are known to deactivate the catalyst,^15^–^17^ so that *exo* and *endo* isomers must be separated using time-consuming techniques in order to achieve high yields. Furthermore, Lewis acids such as alkyl aluminums, methylalumoxane or fluorinated boranes which are cocatalysts of early transition metal catalysts for NBE polymerization usually react with polar monomers.^18^ Nickel-based catalysts have recently been used to catalyze the homopolymerization of polar norbornenes, but with low yields (less than 5%) unless an excess of Lewis acid cocatalyst relative to monomer is used.^19^–^25^ In 1992, Risse *et al.* demonstrated that [Pd(CH_3_CN)_4_][BF_4_]_2_ promoted the living polymerization of a series of esters of bicyclo[2.2.1]hept-5-ene-2-*exo*-methanol.^26^ Later, the same team reported that cationic (η^3^-allyl)palladium compounds with BF_4_^–^ or SbF_6_^–^ counter ions were able to catalyze the polymerization of bicyclo[2.2.1]hept-5-ene-2-carboxylic acid methyl ester (NBECO_2_Me).^27^ Copolymerization of NBE and bicyclo[2.2.1]hept-5-ene-2-carboxylic acid (NBECO_2_H) was also reported. In 1995, Novak *et al.* reported that neutral Pd(ii) complexes bearing an hexafluoroacetyl acetonate and a σ–π bicyclic alkyl ligands catalyze the living polymerization of NBE and a substituted oxanorbornene.^28^ This catalyst, under its neutral form, is unable to catalyze the polymerization of NBECO_2_Me but a cationic analog was found to be active for the polymerization of NBECO_2_Me and the copolymerization of NBECO_2_H with NBE in a non-living manner, with either BF_4_^–^,^29^ SbF_6_^–^ (ref. 30) or MAO^30^ as anion. These studies, and subsequent ones^24^,^25^,^31^–^40^ point out toward the same difficulties: (1) polar norbornenes are little reactive, and therefore the polymerization proceeds with low or moderate yield unless it is copolymerized (*i.e.* diluted) with NBE or with ethylene^38^,^39^,^41^ (2) resulting from the lack of reactivity of polar norbornenes, the polymerization must be performed with high catalyst loadings (>0.5 mol%); (3) the *endo* isomer is practically non-reactive and it deactivates the catalyst. The lack of reactivity of the *endo* substituted polar NBEs^42^,^43^ is believed to be due to the formation of a chelate between the Pd catalyst and the functionality either upon coordination of the monomer, or after insertion into the Pd–C bond. Although the insertion of NBE into Pd–C bonds is generally found to occur *via* the *exo* face,^2^,^44^–^46^ the formation of a chelate requires that the Pd atom adds to the *endo* face of the monomer, as shown by Sen *et al.* through the X-ray structure of an homologous Pt complex.^42^ At the opposite, Nozaki *et al.* has ruled out the formation of chelate for naked cationic Pd complexes stabilized by the ^*t*^Bu_3_P phosphine and found that *endo* isomers are as reactive as *exo* ones.^47^ Obviously, the mechanism of polymerization requires clarification.

**Scheme 1:**
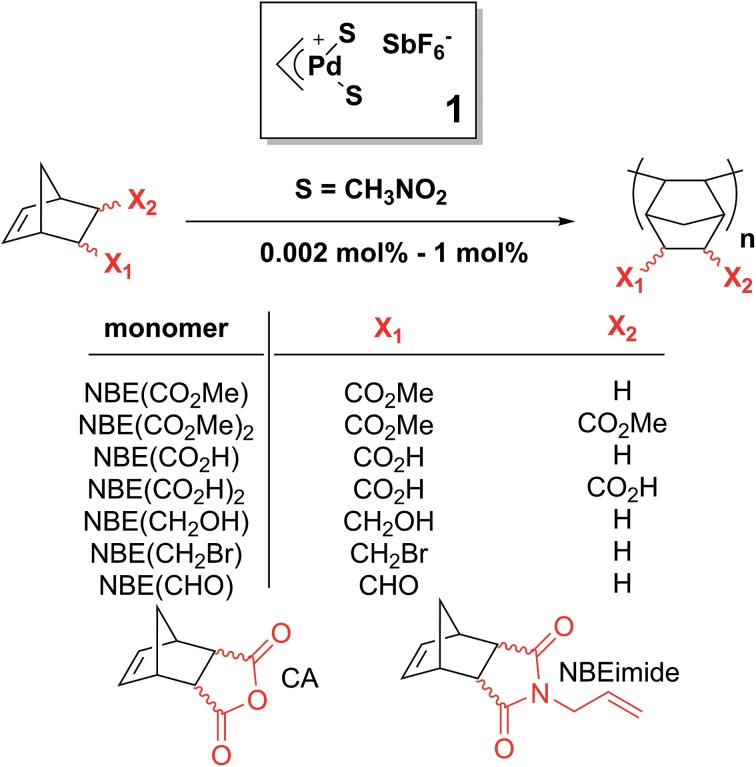
Scope of the rectification–insertion polymerization reaction.

In this paper, we show that the lack of reactivity of the *endo* isomer is due to a series of factors (solubility, chelation and impossibility to insert two *endo* monomers consecutively). Furthermore, the catalyst promotes the rectification of the monomer (*i.e.* the interconversion from *endo* to *exo*, and *vice versa*). Numerous functional PNBEs (Scheme 1) have thus been prepared starting from monomers obtained directly from Diels–Alder reaction and not enriched in *exo* isomer, and using catalyst loadings as low as 0.002 mol%, which contrasts with literature data typically reported at catalyst loading of 1 mol%.

## Results and discussion

Our initial work on the polymerization of polar norbornenes was inspired by the discovery that cationic ‘naked’ Pd and Ni catalysts^2^,^27^ are excellent catalysts for the homopolymerization of NBE. The term naked is used because the metal is believed to be only surrounded by the growing polymer chain, the monomer and/or solvent molecules with no ancillary ligand. These catalysts are cationic in nature, with a counter anion which belongs to the class of weakly coordinating anions.^48^ Several anions (BF_4_^–^, PF_6_^–^, MAO, B(C_6_F_5_)_4_^–^, SbF_6_^–^) have been investigated in the past for the polymerization of NBE, and we have selected SbF_6_^–^ as it is more bulky than BF_4_^–^ and PF_6_^–^, and therefore putatively less coordinating, but it does not require handling pyrophoric MAO and B(C_6_F_5_)_3_.

We started our study with catalyst **1**, [(η^3^-allyl)PdS_2_]^+^SbF_6_^–^ (S = solvent).^27^ The polymerization of NBE(CO_2_Me) (73% *endo*) occurs in low to moderate yield (0–56%) at high catalyst loadings (≥0.2 mol%) in the majority of solvents (Table 1), in good agreement with past observations^27^ which have emphasized the difficulties associated with the polymerization of *endo* rich monomers. Surprisingly, in nitromethane, the polymerization proceeds with catalyst loadings as low as 0.02 mol%. Coordinating solvents such as DMSO, acetonitrile, acetone, water, DMF, methanol, ethyl acetate or THF efficiently solvate the ionic catalyst, but also inhibits monomer coordination (see Fig. 2 for catalyst structures coordinated by THF and water). Non-polar solvents such as tetrachloroethane, chlorobenzene or dichloromethane are only weakly (if at all) competing for the vacancy, resulting in yields which are higher, but the polymerization is then limited by the lack of solubility of the growing polymer chain: the polymerization eventually stops due to the precipitation of the cationic Pd active site anchored to the polymer, as visually observed. Nitromethane offers an acceptable compromise between catalytic deactivation *via* coordination and loss of solubility.

**Table 1:** Polymerization of NBE(CO_2_Me) (73% *endo*) with catalyst **1**: influence of solvent and catalyst loading

Catalyst loading (mol%)							
Solvent	0.5	0.2	0.1	0.02	0.01	0.005	0.003
	Yield^*a*^ (%)						
DMSO	0	0	0	0	0	0	0
CH_3_CN	0	0	0	0	0	0	0
Acetone	0	0	0	0	0	0	0
DMF	0	0	0	0	0	0	0
CH_3_OH	0	0	0	0	0	0	0
THF	6	0	0	0	0	0	0
Et_2_O	36	16	0	0	0	0	0
EtOAc	73	18	0	0	0	0	0
Toluene	81	31	0	0	0	0	0
C_6_H_5_Cl	92	39	0	0	0	0	0
DCM	96	56	0	0	0	0	0
CH_3_NO_2_	100	85	65	14	0	0	0
Neat	68	65	65	50^*b*^	35^*b*^	18^*b*^	20^*b*^

^*a*^Monomer concentration = 2 g in 6.5 g solvent, 72 hours at room temperature.

^*b*^ *T* = 70 °C.

When the polymerization is performed neat (no solvent excepted traces of nitromethane during catalyst preparation), the yield is limited to 65% (catalyst loadings of 0.5–0.1%). This limitation is purely physical in nature, and corresponds to a vitrification phenomenon: a mixture of 65% of polymer and 35% of monomer is so viscous that the monomer cannot diffuse to reach the active site. Thus, the yield of the neat polymerization modestly decreases when the catalyst loading is lowered, so that the homopolymerization of NBE(CO_2_Me) becomes feasible even with a catalyst loading as low as 0.003 mol% which contrasts with past results reported in literature whereby catalyst loadings are typically comprised between 0.5 and 1%. In this study, all mechanistic studies have been performed in nitromethane as it keeps the polymer in solution without coordinating too strongly catalyst **1**.

**Fig. 1:**
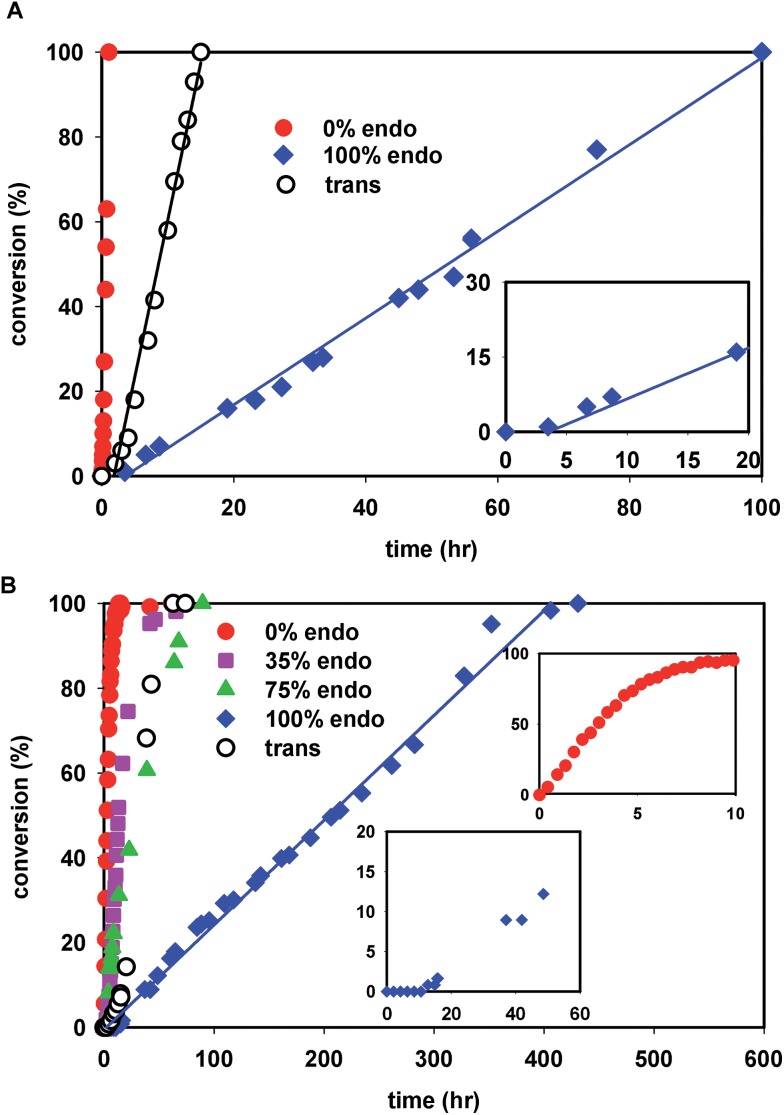
(A) Kinetic plot for the polymerization of NBE(CO_2_Me)_2_ by 1 in nitromethane ([NBE(CO_2_Me)_2_]/[**1**] = 100, [NBE(CO_2_Me)_2_] = 2.4 mol L^–1^, *T* = 50 °C) and inset: zoom of the *endo*-NBE(CO_2_Me)_2_ kinetic plot. (B) Kinetic plot the polymerization of NBE(CO_2_Me)_2_ by 1 ([NBE(CO_2_Me)_2_]/[**1**] = 10, [NBE(CO_2_Me)_2_] = 0.34 mol L^–1^, *T* = 25 °C) and insets: zoom of the *exo*-NBE(CO_2_Me)_2_ kinetic plot and of the induction period for the *endo*-NBE(CO_2_Me)_2_ polymerization. The lines correspond to linear fits.

The kinetics of polymerization of *cis*-NBE(CO_2_Me)_2_ (abbreviated NBE(CO_2_Me)_2_) and *trans*-NBE(CO_2_Me)_2_ (one *endo* and one *exo* CO_2_Me group) are illustrated in Fig. 1. The rate of polymerization sharply decreases with increasing *endo* content, the kinetics of *trans* isomer being comprised between the one of 35% *endo* and of 75% *endo*, and therefore being comparable to 50% *endo*. When the *endo* content in the monomer is greater than 25%, the polymerization is zero order in monomer, as shown by a linear evolution of the conversion *vs.* time, a behavior which is also observed for the polymerization of other monomers with **1** (Fig. S35†). For the *exo* monomer, the kinetics deviates from zero order when [NBE(CO_2_Me)_2_]/[**1**] = 10 (Fig. 1B, inset). Furthermore, an induction period of a few hours is observed for the polymerization of the 100% *endo* monomer (Fig. 1A and B, insets).

**Scheme 2:**
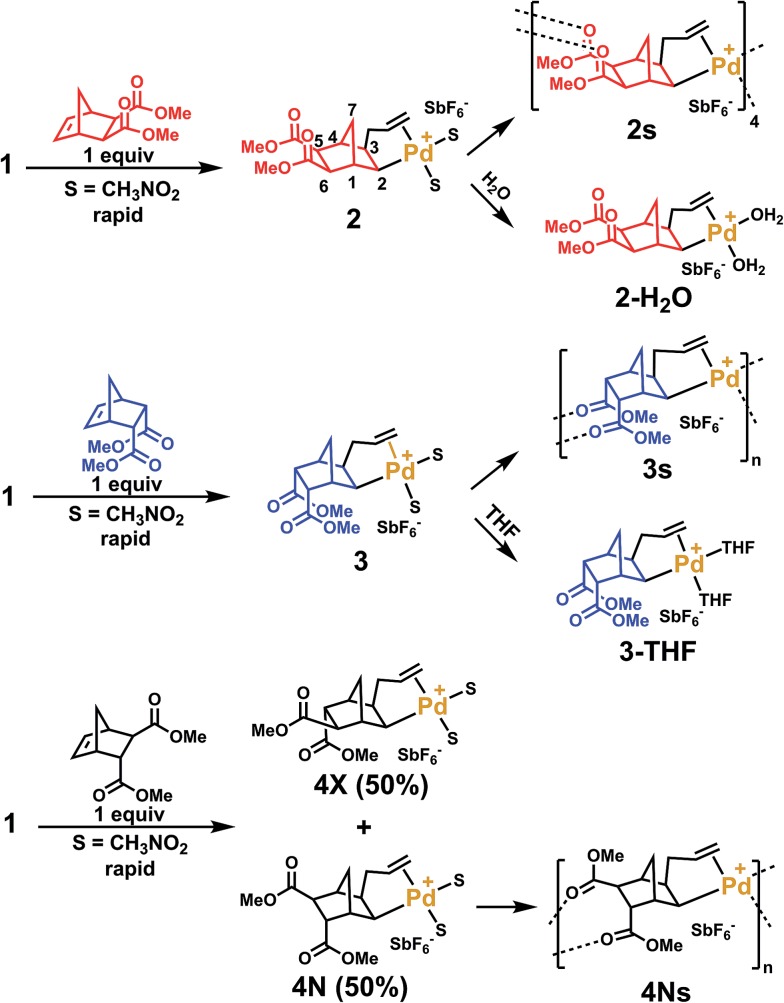
Catalysts **2**, **3** and **4** obtained upon single insertion of NBE(CO_2_Me)_2_ in 1.

**Fig. 2:**
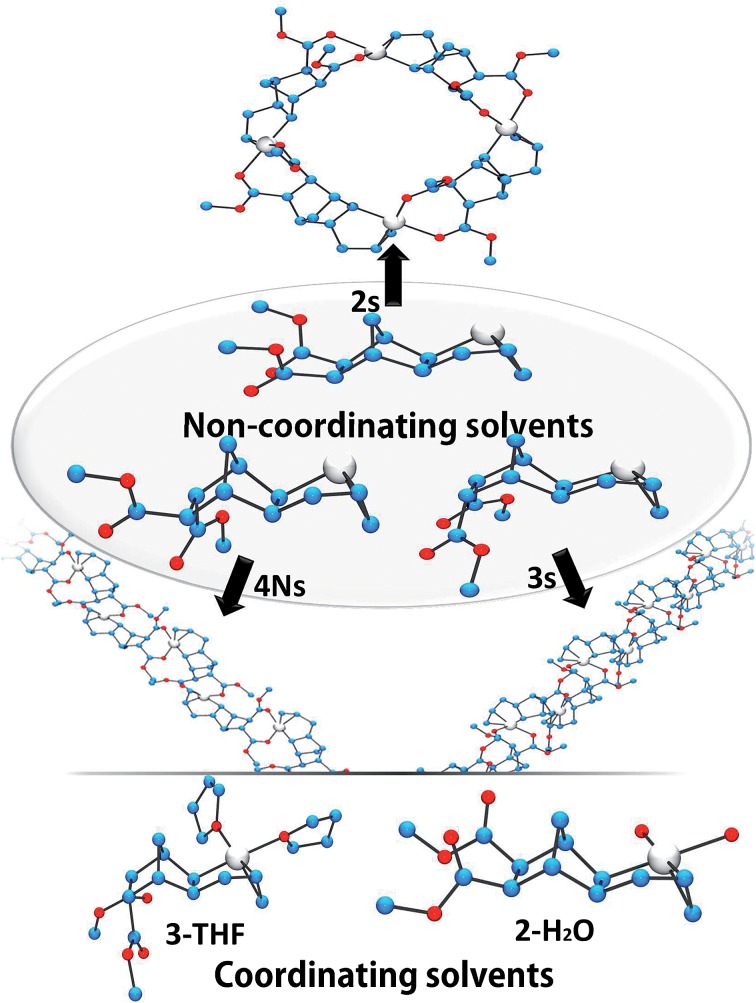
Solid-state structures of catalysts **2s**, **3s**, **4Ns**, **2-H_2_O** and **3-THF**. Solvent molecules and SbF_6_^–^ anions are omitted for clarity. Compound **2s** is a tetrameric macrocycle, **3s** and **4Ns** are polymeric (monomeric units shown within the ellipse).

In order to clarify the mechanism of polymerization, *exo*, *endo* and *trans* NBE(CO_2_Me)_2_ were inserted in **1**, leading to the formation of catalysts **2**, **3** and **4** respectively (Scheme 2), which were fully characterized either by nuclear magnetic resonance (^1^H, ^13^C, DEPT, COSY and HMQC) and by X-ray crystallography (Fig. 2). The reaction of **1** with either *endo*, *exo* or *trans* NBE(CO_2_Me)_2_ is rapid, *i.e.* it is quantitative in less than a minute at room temperature (compared to several hours of polymerization, Fig. 1). The addition of the Pd–C bond to the double bond of NBE(CO_2_Me)_2_ occurs in a *cis* fashion on the *exo* face, as shown in X-ray structures. The ^3^J coupling value between protons H_2_ and H_3_ is comprised between 6 and 8 Hz which is characteristic of a *cis* coupling (see Scheme 2 for atom numbering). In NBE structures, ^3^J couplings between bridgehead protons and protons in *endo* positions are less than 2 Hz, whereas couplings with protons in *exo* positions are of the order of 3–4 Hz.^49^ No ^3^J coupling between protons H_1_ and H_2_ or H_3_ and H_4_ could be observed in our case, which is consistent with a Pd attack on the *exo* face of the monomer. It has been proposed in the past that catalyst addition could occur on the *endo* face of *endo*-substituted monomers, because of the directing effect of the functional group.^42^ In our case, coupling patterns of all Pd species characterized in this work (^3^J between protons H_1_ and H_2_ or H_3_ and H_4_) are consistent with an *exo* placement of the Pd atom, ruling out the presence of a directing effect. Addition of NBE(CO_2_Me)_2_ (*trans*) to **1** results in the formation of two products (**4X** and **4N**) in 50 : 50 mol% ratio, as shown by ^1^H NMR (Fig. S11†). In the **4X** (resp. **4N**) product, the Pd atom is on the same side of the bridge as the *exo* (resp *endo*) CO_2_Me. The fact that **4X** and **4N** are in equal proportion is another indication that the *endo* ester does not act as a directing group for catalyst addition. In solution, the ester groups of the inserted NBE(CO_2_Me)_2_ are not coordinated, as shown by ^13^C NMR chemical shifts comprised between 173.2 and 175.5 ppm for C=O of complexes **2**, **3**, **4N** and **4X**, which correspond to the usual chemical shifts of CO_2_Me esters. For the sake of comparison, in a norbornane ring bearing two CO_2_Me groups in *exo* (resp *trans*), the C=O resonates at 173.1 ppm (ref. 50) (resp 173.6 and 174.6 ppm)^51^ whereas coordinated CO_2_Me either on these naked Pd complexes (see below) or on cationic Pd diimine complexes are found at lower field (177–195 ppm).^52^

Crystals of complexes **2**, **3** and **4N** were formed by adding a non-coordinating solvent and/or by letting the solvent evaporate slowly (the letter **s** stands for solid-state structure). Structure **2s** is a tetrameric macrocycle whereas **3s** and **4Ns** are polymeric (Fig. 2). In these solid-state structures, intermolecular coordination of the ester is observed, however, when the crystallization experiment is performed in the presence of a stronger Lewis base, such as water and THF, monomeric complexes (**2-H_2_O** and **3-THF**) are obtained. The tetrameric solid **2s** is soluble in non-coordinating solvents such as dichloromethane, chlorobenzene and tetrachloroethane however, polymeric **3s** and **4Ns** are insoluble in such solvents. Therefore, when the polymerization is performed in a non-coordinating solvent, the insertion of *endo* and the *trans* monomer leads to the formation of an insoluble active species (**3s** and **4s**). Thus, the lack of reactivity of *endo* isomers in chlorinated solvents is in part explainable by the loss of Pd-containing species by precipitation. Interestingly, characteristic bond lengths and bond angles of complexes **2s**, **3s**, **4Ns**, **2-H_2_O** and **3-THF** (Table S1†) are quite similar, once again pointing out that there is no significant structural difference for the first insertion product of the *endo* and *exo* monomers. However kinetic plots in nitromethane (Fig. 1) point out toward a very different reactivity for *endo* and *exo* isomers. Thus differences must occur after the first monomer insertion.

**Scheme 3:**
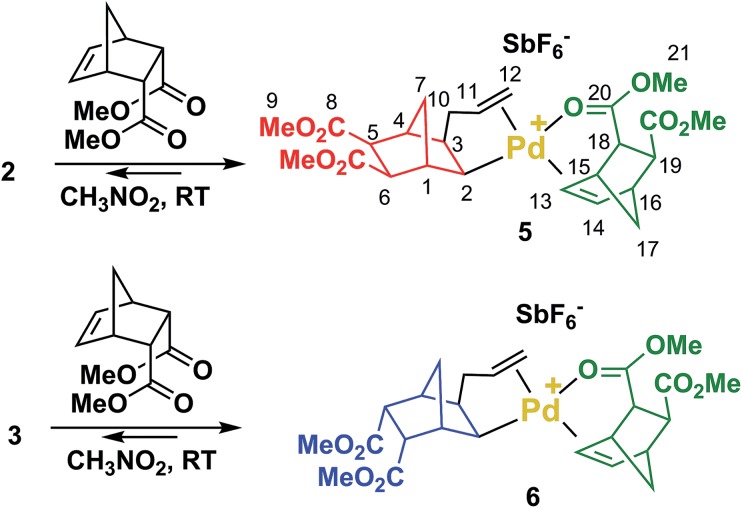
Reaction of NBE(CO_2_Me)_2_ (*endo*) with **2** and **3** to yield **5** and **6** respectively.

The reaction of **2** or **3** with 1 equivalent of *endo*-NBE(CO_2_Me)_2_ at room temperature result in the immediate formation of **5** and **6** (Scheme 3), which were fully characterized by NMR (^1^H, ^13^C, DEPT, COSY and HMQC). In these complexes, *endo*-NBE(CO_2_Me)_2_ is chelated *via* its *endo* face to the Pd complex, as deduced by an unexpected downfield resonance for the HC_13_=C_14_H moiety (^1^H NMR: 7.02 for **5**, 7.22 ppm for **6***vs.* 6.29 ppm for the uncoordinated monomer and ^13^C NMR: 135.7 for **5** and 135.4 ppm for **6***vs.* 134.7 ppm for the uncoordinated monomer). The ^13^C carbonyl resonances are also shifted downfield (177.5 and 177.1 respectively for **5** and **6***vs.* 172.7 ppm for the uncoordinated monomer). This chelate is labile and rapidly exchanging within NMR timescale. For example, from 7.05 ppm in **5**, the H_13/14_ resonance is displaced to 6.83 ppm for a 1 : 1 mixture of **5** and *endo*-NBE(CO_2_Me)_2_, and to 6.48 ppm for a 1 : 10 mixture of **5** and *endo*-NBE(CO_2_Me)_2_. Such exchange phenomenon is not observed when an excess of *exo* monomer is added to **5** or **6** as shown by separate resonances for the chelated *endo* monomer and free *exo*-NBE(CO_2_Me)_2_ (Fig. S26 and S28†). Therefore, if it occurs, displacement of the chelated *endo* monomer by the *exo* monomer is slow.

When **2** is reacted with 10 equivalents of *exo*-NBE(CO_2_Me)_2_, polymerization is quantitative within 9 hours, as shown by the decrease of the olefin resonances in ^1^H NMR at 6.35 ppm. To our surprise, although no *endo* monomer was added to this reaction, the presence of the chelated complex **5** is also clearly detected in the reaction mixture, as shown by the presence of (1) a new downfield olefin resonance (6.8–7.0 ppm) characteristic of the *endo* chelated double bond, (2) a OCH_3_ resonance at 3.81 ppm which is neither observed in **2** (3.74 ppm) nor in the *exo* growing polymer chain (3.74 ppm) and (3) a characteristic H_17_ bridge proton for the chelated *endo* monomer at 1.71 ppm (Fig. S26†). The *exo* monomer used in this study contained traces of *endo* monomer (less than 2%) which could account for a maximum of 20% of the chelated Pd complexes (10 equivalents of monomer were used relative to Pd). However, as much as 70% of the Pd atoms are found to be chelated. Therefore, the *endo* isomer must be generated during the polymerization reaction. Lewis acids are known to catalyze direct and retro Diels–Alder reactions,^53^ and therefore Lewis acidic cationic Pd complexes could be responsible for the transformation of the *exo* monomer in *endo* monomer. Although is it well known that the *exo* monomer (thermodynamic product) is more stable than the *endo* monomer (kinetic product), DFT calculations indicate that the gain of stability is only 2.7 kJ mol^–1^. Therefore, the equilibrium constant between both isomers is 3, and the *exo*–*endo* monomer distribution at equilibrium is 75 : 25. Thus, even when starting from 100% *exo* monomer, *endo* isomer is generated during the polymerization. It will be seen below that the reverse situation is also true, that is to say that when starting from pure *endo* monomer, *exo* monomer is generated during the reaction.

When the *exo* monomer is polymerized by **2**, the Lewis-acid catalyzed formation of the *endo* isomer leads to the generation of chelated **5** which is significantly less active for polymerization. Thus, only a fraction of the catalyst is 'naked' and active, resulting in the formation of polymers with molecular weights which are higher than expected (Table 2, entry 5, 10, 15–17). The higher than expected molecular weights may also originate from the low initiating-ability of catalyst **2**. The initiation of the polymerization of *exo*-NBE(CO_2_Me)_2_ by **2** (rate constant *k*_i_) is slow relative to subsequent insertions (*k*_p_) as the Pd–C2–C3–C10–C11–C12 6-member chelate must be broken during the first insertion. When polymerizing *endo*-NBE(CO_2_Me)_2_, the propagation rate (*k*_p_) is greatly decreased, thus the discrepancy between *k*_p_ and *k*_i_ is less noticeable. As the result, experimental and theoretical molecular weights are in good agreement for *endo* monomers (Table 2, entry 1, 6, 11). The *in situ* formation of chelated **5** during the polymerization of *exo*-NBE(CO_2_Me)_2_ explains the deviation from linearity observed in the polymerization kinetics at high conversion (see inset Fig. 1B). Initially, all the active sites are unchelated, and the polymerization proceeds rapidly. As the polymerization progresses, the *endo* isomer is generated *in situ*, leading to the gradual formation of chelated catalysts which are less active, and resulting in a decrease of the polymerization rate. Such effect is not observed when polymerizing *endo*-NBE(CO_2_Me)_2_ or mixtures of *endo* and *exo* NBE(CO_2_Me)_2_, as the catalyst is then entirely chelated from the onset of the polymerization.

**Table 2:** Polymerization of NBE(CO_2_Me)_2_ (*c* = 4.76 mol L^–1^, *T* = 70 °C) in nitromethane

Expt	*Endo* (%)	Cat loading mol%	Yield/%	*M*_n,theor_ g mol^–1^	*M*_n_ ^*a*^ g mol^–1^	PDI^*a*^
1	100	1	40 (59)^*b*^	12 400	13 100	1.3
2	75	1	83	17 400	26 000	1.5
3	*Trans*	1	92 (99)	20 800	53 000	1.3
4	35	1	98	20 600	68 500	1.9
5	0	1	100	21 000	241 000	1.2
6	100	0.2	15 (21)	22 000	17 000	1.3
7	75	0.2	56	59 000	43 000	1.5
8	*Trans*	0.2	55	58 000	87 000	1.6
9	35	0.2	85	88 000	66 400	1.6
10	0	0.2	100	105 000	380 000	1.3
11	100	0.1	8 (10)	21 000	20 700	1.4
12	75	0.1	37 (39)	82 000	66 000	1.5
13	*Trans*	0.1	16 (42)	88 000	59 000	1.3
14	35	0.1	65 (69)	145 000	84 000	1.4
15	0	0.1	100	210 000	389 000	1.5
16	0	0.02	(13)	136 000	509 000	1.2
17	0	0.01	(4)	84 000	316 000	1.4

^*a*^Number average molecular weight and polydispersity index determined by GPC-LS in THF.

^*b*^Without ( ): 24 hours reaction, with ( ): 72 hours reaction.

**Scheme 4:**
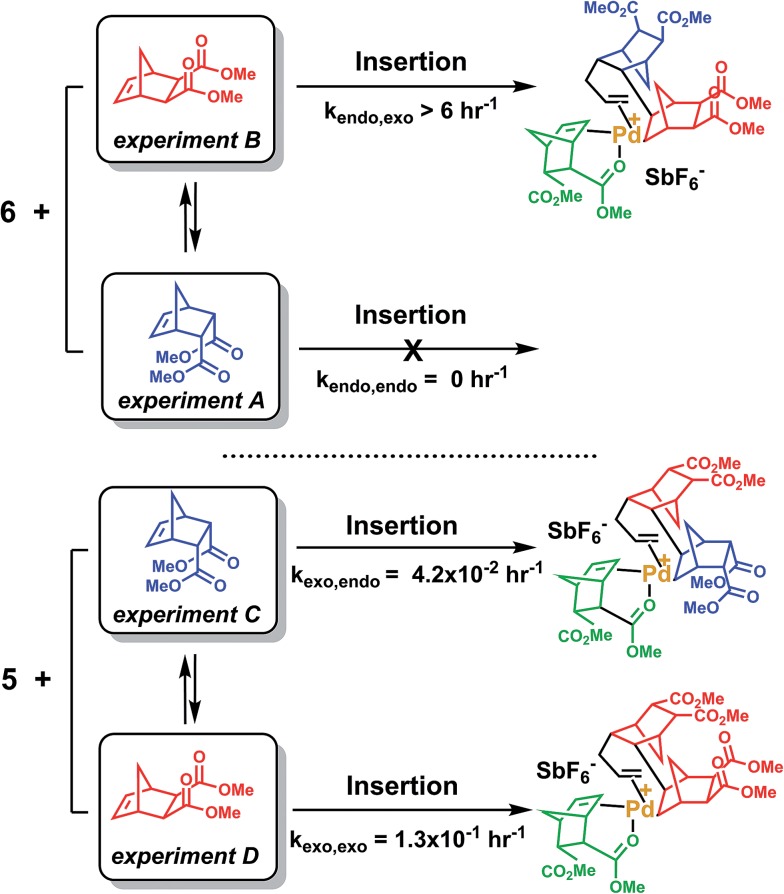
Reaction of NBE(CO_2_Me)_2_ with **5** or **6**. Only the first insertion is shown for the sake of simplification.

**Fig. 3:**
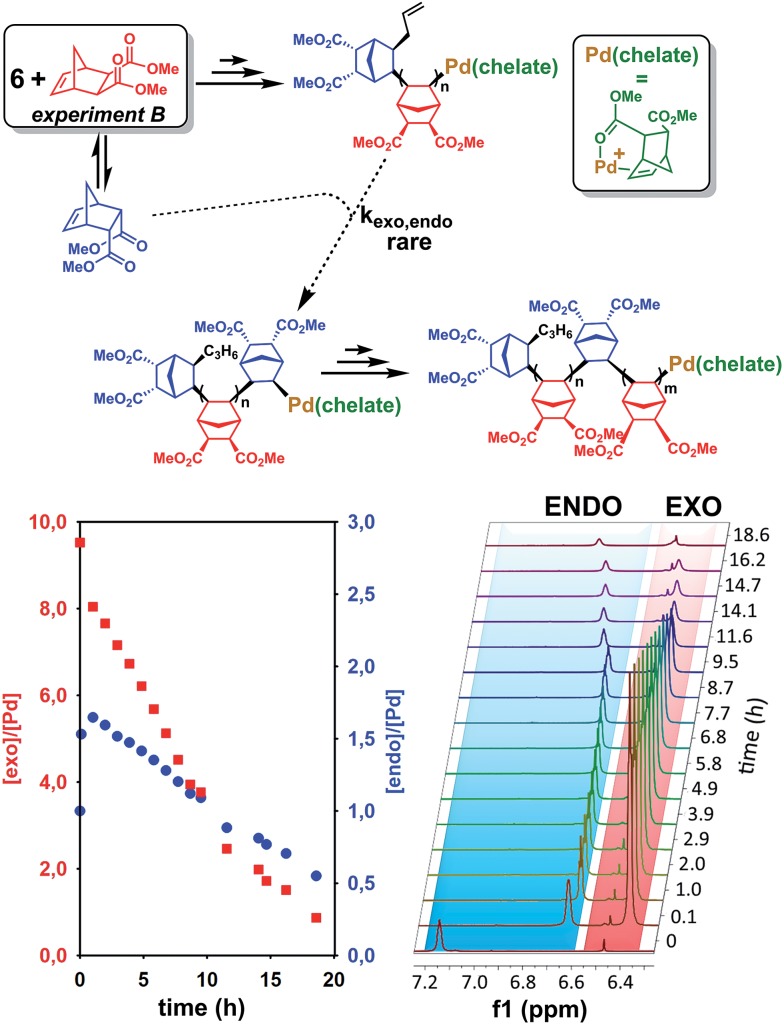
Polymerization of *exo*-NBE(CO_2_Me)_2_ (10 eq.) by **6** (experiment B). Left: equivalents of *endo* and *exo* monomers *vs.* time, right: overlay of the ^1^H NMR spectra (olefinic region) *vs.* time. The olefinic resonance at 6.9–7.1 ppm corresponds to the double bond of the *endo* monomer which is in rapid exchange between the free form (at 6.29 ppm) and the chelated form (at 7.2 ppm, as seen at *t* = 0).

The last paragraph pertained to the polymerization of *exo*-NBE(CO_2_Me)_2_. However, it is desirable to polymerize monomers directly obtained by Diels–Alder reaction, that is to say rich in *endo* isomer in order to avoid a painstaking separation step between *endo* and *exo* isomers. In this case, due to the presence of excess *endo* isomer, the catalyst is entirely chelated. Therefore, we will now concentrate on the reactivity of catalysts **5** and **6**. Using ^1^H NMR, one can assess the regiochemistry of the last inserted monomer unit. Indeed, the methine proton in α of Pd (H_2_, Scheme 3) resonates respectively at 4.3 and 3.9 ppm when it is part of an *endo* and an *exo* unit. When **6** is reacted with 10 equivalents of *endo*-NBE(CO_2_Me)_2_ (experiment A in Scheme 4), polymerization occurs very slowly over a period of 500 hours. During the first 9 hours, no polymerization is observed (corresponding to an induction period, see inset Fig. 1B). By contrast, when **6** is reacted with 10 equivalents of *exo*-NBE(CO_2_Me)_2_ (experiment B), insertion of a first *exo* monomer occurs within minutes (Fig. S28†). Thus, the addition of an *exo* monomer after an *endo* unit is very rapid (experiment B), whereas the addition of two consecutive *endo* units is very slow (experiment A). Using the usual denomination for copolymerization rate constants, *k*_*endo*,*exo*_ ≫ *k*_*endo*,*endo*_. The value of *k*_*endo*,*exo*_ is too high to be measured precisely *via*^1^H NMR, but a lower limit for *k*_*endo*,*exo*_ could be determined on the account that the insertion of one *exo* monomer by **6** proceeds in less than 10 minutes (Fig. S28 and S32†), that is to say a minimum of 6 insertions per hour, thus, *k*_*endo*,*exo*_ ≥ 6 h^–1^. The isomerization of *exo*-NBE(CO_2_Me)_2_ in *endo*-NBE(CO_2_Me)_2_ is clearly observed in experiment B (Fig. 3). At the beginning of the polymerization, the only *endo* monomer present is engaged in the chelate **6** (1 equivalent relative to Pd), but soon after, 1.6 equivalents of *endo* monomer are present (Fig. 3). Despite the presence of a significant amount of *endo* monomer, its insertion is never observed (no *endo* H_2_ protons, Fig. S28†). This could either be due to the fact that the *endo* monomer is non-reactive (it is not inserted), or that the insertion of *endo* monomer is immediately followed by the insertion of an *exo* monomer, due to the high value of *k*_*endo*,*exo*_. To lift this ambiguity, we have examined the reaction of **5** with 10 equivalents of *endo*-NBE(CO_2_Me)_2_ (experiment C in Scheme 4, Fig. S29†).

In experiment C, *endo*-NBE(CO_2_Me)_2_ is inserted, as shown by the apparition over a few hours of a characteristic doublet at 4.3 ppm corresponding to H_2_ in an *endo* unit. Thus, the *endo* monomer is reactive and *k*_*exo*,*endo*_ is non null. The low stability of catalyst **5** in solution is another indication that the *endo* isomer can be inserted after an *exo* unit. Indeed, when a solution of **5** in CD_3_NO_2_ ([**5**] = 0.032 mol L^–1^) is left for 7 hours, 30% of the chelated *endo* monomer is inserted (Fig. S30†). To determine the value of *k*_*exo*,*endo*_, the initial rate at which the *endo* monomer is inserted into the Pd-*exo*-C2 bond has been measured in three separate experiments (experiment C performed with respectively 0.5, 1 and 7 equivalents of *endo* NBE(CO_2_Me)_2_, Fig. S33†). The reactions are zero order with respect to monomer concentration (as shown by linear kinetic profiles, Fig. S33†), probably because the rate determining step is the insertion of the coordinated monomer in the Pd–C bond. The values of *k*_*exo*,*endo*_, obtained from the slope of the kinetic profiles, are respectively 3.6 × 10^–2^, 4.1 × 10^–2^ and 4.9 × 10^–2^ h^–1^, which yield an average value of *k*_*exo*,*endo*_ of 4.2 × 10^–2^ h^–1^ (Table 3).

**Table 3:** Rate constants for the copolymerization of *exo* and *endo* NBE(CO_2_Me)_2_ and reactivity ratios, *r*

Rate constant, h^–1^		Reactivity ratio
*k*_*exo*,*exo*_	1.3 × 10^–1^	*r*_*exo*_ = 3.1
*k*_*exo*,*endo*_	4.2 × 10^–2^	
*k*_*endo*,*exo*_	>6	*r*_*endo*_ = 0
*k*_*endo*,*endo*_	∼0	

**Scheme 5:**
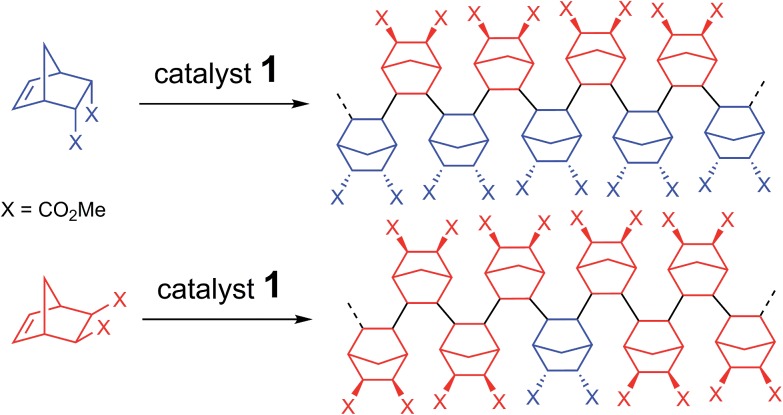
Polymerization *via* the rectification–insertion mechanism of functional PNBEs prepared from respectively *endo* and *exo* monomers.

Contrasting with the lack of stability of **5** in solution (insertion of the *endo* chelate in Pd-*exo*-C2), solutions of **6** are stable indefinitely (Fig. S31†), indicating that *k*_*endo*,*endo*_ ∼0. Therefore, two *endo* units cannot be inserted consecutively. The presence of an induction period for experiment A can be explained by the necessity for the catalyst to interconvert some of the *endo*-NBE(CO_2_Me)_2_ into *exo*-NBE(CO_2_Me)_2_ before polymerization can proceed. The value of *k*_*exo*,*exo*_ was measured in two separate kinetic experiments (experiment D in Scheme 4 conducted with respectively 3 and 10 equivalents of *exo* monomer, Fig. S34†), leading to *k*_*exo*,*exo*_ = 0.13 h^–1^. Once again, the kinetics is zero order with respect to monomer concentration. From the determination of the rate constants and reactivity ratios (Table 3), it is clear that the polymerization of functional polar norbornenes present some unique features. As *r*_*endo*_ = 0, an *endo* unit is always isolated in the chain. When polymerizing an *exo* monomer, *endo* monomers are generated *via* retro Diels–Alder reaction and inserted as isolated units within the mainly *exo* chain (Scheme 5). When an *endo* monomer is polymerized, an *exo* monomer generated by retro Diels–Alder reaction is immediately inserted due to the high value of *k*_*endo*,*exo*_; therefore the monomer feed is nearly exclusively constituted of *endo* monomer. Although the addition of an *exo* monomer after an *exo* unit is 3.1 times faster than the addition of an *endo* monomer (*r*_*exo*_ = 3.1), the insertion of two *exo* monomers consecutively is highly unlikely due to the low concentration of *exo* monomer in solution. Thus, the polymerization of the *endo* monomer leads to the formation of an alternating *endo*–*exo* copolymer. The catalyst has rectified 50% of the less reactive *endo* monomers into more reactive *exo* monomers. We have coined such mechanism rectification–insertion.

Microstructural analysis of these polymers, either by ^1^H NMR or ^13^C NMR has been hampered by the broadness of the peaks caused by the rigidity of polynorbornenes in solution (Fig. S37–48†). For the polymer prepared with *exo* NBE(CO_2_Me)_2_, the ^13^C spectrum is constituted of very broad resonances, with a single peak observed at 173.1 ppm for C=O (Fig. S37–38†). The complete absence of fine structure is consistent with the presence in the polymer of *exo* and *endo* units statistically distributed. In contrast, for the polymer prepared with the *endo* monomer, the ^1^H and ^13^C NMR spectra are constituted of slightly sharper peaks, which are consistent with the regioregular structure of the alternating polymer. Three resonances observed at 172.4, 173.4 and 174.3 ppm in a ∼2 : 1 : 1 ratio respectively (Fig. S39†) could conceivably correspond to one resonance for *endo* C=O and two resonances for *exo* C=O (*endo* C=O are upfield relative to *exo* ones in **2**, **3**, **5** and **6**). The presence of two *exo* C=O peaks in same proportion could arise from two different tacticities arising from the placement of consecutive NBEs.

In order the rectification–insertion mechanism to be operating, two conditions must be met. First, the *endo* isomer must be interconverted into *exo*. It is also well established that Lewis acids (such as **1**) catalyze the Diels–Alder and retro Diels–Alder reaction.^53^–^55^ Since all the monomers of this study (Table 4) have been prepared by Diels–Alder reaction in one step, such interconversion is expected, albeit it is anticipated to be slower for poor dienophiles such as allyl alcohol. In the case of NBE(CHO), the occurrence of retro Diels–Alder reaction during the polymerization is revealed by the presence of inserted dicyclopentadiene within the polymer (acroleine loss by evaporation). We also have checked that dicyclopentadiene can be homopolymerized by **1** (yield = 30% for 0.2 mol% catalyst loading at 70 °C). Interestingly, freshly cracked cyclopentadiene can also be polymerized with **1**, leading to a polymer which is not entirely similar to polydicyclopentadiene by ^1^H NMR (precise analysis of this polymer is beyond the scope of this paper, as it not a norbornene polymer). Second, *k*_*endo*,*endo*_ must be significantly lower than the other propagation rate constants. Preliminary theoretical calculations indicate that *k*_*endo*,*endo*_ is very low because of the large steric hindrance between the *endo* substituents of the penultimate inserted unit and the active site when two *endo* monomers are inserted in a row. Several elements point toward the fact the rectification–insertion mechanism is not only prevailing with NBE(CO_2_Me)_2_ but also with other functionalized norbornenes. Induction periods are observed for the polymerization of predominantly *endo* monomers (Fig. S35†), whereas induction periods are not observed for predominantly *exo* monomers. This induction period corresponds to the time period necessary to rectify the *endo* isomer into the *exo* isomer in order to unblock the *endo*-terminated growing chain. Furthermore, the ^13^C NMR spectra of polymers prepared with mostly *endo* monomer or mostly *exo* monomer are not clearly different, and contain overlapping resonances, which is consistent with polymers which are constituted of both *endo* and *exo* units. With the rectification–insertion mechanism in operation, *endo* isomers become polymerizable. Thus, by extension, the polymerization can proceed with monomers containing as much as 70–100% *endo* isomer, that is to say, monomers obtained directly from Diels–Alder reaction which are not enriched in *exo* isomer (Table 4).

**Table 4:** Polymerization of functional NBEs (*T* = 70 °C)

Monomer	*Endo* (%)	Cat. loading (mol%)	Yield/%	*M*_n_ ^*c*^ g mol^–1^	PDI^*c*^
NBE(CO_2_Me)^*a*^	73	0.1	65	46 000	1.2
NBE(CO_2_Me)^*b*^	73	0.01	35	63 000	1.5
NBE(CO_2_H)^*a*^	75	0.1	71	31 000	1.2
NBE(CO_2_H)^*b*^	75	0.1	57	24 000	1.2
NBE(CO_2_H)^*b*^	75	0.01	40	252 000	1.3
NBE(CO_2_H)^*b*^	45	0.1	55	81 000	1.7
NBE(CO_2_H)^*b*^	45	0.01	40	477 000	2.1
NBE(CO_2_H)^*b*^	45	0.002	15	609 000	2.6
NBE(CO_2_H)_2_^*a*^	0	1	93	81 000	1.5
CA^*a*^	0	0.5	83	146 000	2.1
CA^*a*^	0	0.2	13	315 000	1.3
NBE(CH_2_Br)^*b*^	86	0.2	15	14 700	1.2
NBE(imide)^*a*^	35	1	80	8050	1.3
NBE(imide)^*a*^	35	0.1	45	97 000	1.6
NBE(imide)^*a*^	5	1	80	57 800	1.1
NBE(CH_2_OH)^*b*^	82	0.2	53	14 000	1.3
NBE(CHO)^*b*^	80	0.2	73	11 600	1.6
NBE(CHO)^*b*^	80	0.02	21	13 200	1.9

^*a*^In solution in CH_3_NO_2_.

^*b*^Without solvent.

^*c*^Determined by GPC (see ESI for conditions).

Thanks to this mechanism, catalyst **1** proved to be active for a wide array of monomers (Tables 1, 2 and 4). The catalyst is very versatile, as it is able to function in the presence of alcohols, esters, carboxylic acids, anhydrides, aldehydes, alkyl bromides, and amides. To our knowledge, out of the nine monomers probed here, four had never been polymerized before (CA, NBE(CO_2_H)_2_, NBE(imide), and NBE(CHO)). As a proof of the unique versatility of this reaction, the polymerization of aldehyde containing monomer NBE(CHO) was found to proceed in high yield (73% at 0.2 mol% catalyst loading) which contrasts with radical, cationic and anionic polymerizations which are usually not efficient to prepare linear polymers containing pendant aldehyde groups.^56^ Furthermore, this method is not only efficient for organosoluble but also for water soluble polymers such as PNBE(CO_2_H)_2_.

When the polymerization is performed in the absence of solvent, very low amounts of catalyst can be used (as low as 0.002 mol%), which is indicative of the exceptional robustness of the active species. The polymerization is then controlled by the drastic increase of viscosity associated with the formation of high Tg polymers, a physical limitation which could for example be mitigated by the use of heterophase processes. The polymers have in general low polydispersity indices (1.1 ≤ PDI ≤ 1.6), indicating some degree of livingness for this type of polymerization (a feature which will be further explored in a subsequent report). Monomers with high *exo* content (PNBE(imide) 5% *endo*, PCA, PNBE(CO_2_H)_2_) lead to polymers with a molecular weight higher than expected. As shown above, this behavior is a consequence of the rectification–insertion mechanism: even when starting from pure *exo* monomer, *endo* chelated species can be formed, and only the remaining (unchelated) fraction is rapidly polymerizing. When the monomer contains high amount of *endo* isomer, experimental molecular weights are commensurate with theoretical values, which is again indicative of a high degree of control for the polymerization. All these polymers exhibit Tg which are higher than 300 °C (Fig. S48†), which is a consequence of the high rigidity of the PNBE backbone. Finally, it should be noted that the polymerization is highly tolerant, as monomers could even be polymerized in air (catalyst **1** was prepared under nitrogen) with virtually identical yields to those obtained under inert atmosphere. For example, the polymerization of NBE(CO_2_H) (75% *endo*, catalyst = 0.01 mol%, 24 hours at 70 °C) occurs in 40% and 42% yield when performed respectively under inert atmosphere and in air.

## Conclusions

The novel rectification–insertion polymerization mechanism is a powerful mechanism for the preparation of rigid macromolecules obtained from polar NBEs, yielding functional polymers bearing highly valuable functional groups such as aldehydes, anhydrides, alcohols, alkyl halogens and carboxylic acids. Thus, this method offers the same level of versatility and practicality as highly popular chain-growth polymerizations such as ROMP or radical polymerizations. The reaction readily proceeds with *endo*-rich monomers directly obtained *via* Diels–Alder reaction with no need for cumbersome and time-consuming separation of both isomers. Furthermore, catalyst loadings as low as 0.002 mol% can be used, and both the monomer preparation and the polymerization can be performed in the absence of any solvent. Thus, the preparation of these rigid macromolecules is an archetypal example of green chemical process. This study has also aimed at clarifying the mechanism of polymerization of substituted NBEs. The *endo* isomers deactivate the catalyst because the *endo* active species are less soluble than the *exo* ones and the *endo* monomer forms a chelate with the naked catalyst. However, these limitations can be counteracted by the judicious choice of polymerization conditions, and, most importantly, by the action of the rectification–insertion mechanism. Thus, the naked Pd complex has a tandem role of polymerization catalyst and of *endo*/*exo* isomerization catalyst. As two *endo* units cannot be inserted consecutively, it is possible to prepare alternating *endo*–*exo* copolymers when starting from an *endo* monomer only. We envision that this novel mechanism could be easily exploited further, for example by adding a separate Lewis acid which could catalyze the retro/direct Diels–Alder reaction, which should putatively lead to a rate acceleration and the disappearance of the induction period. Furthermore, due to high degree of control of these polymerization, we believe that this mechanism open the way to the formation of a wealth of hierarchical nanostructures generated upon self-assembly of rigid functional amphiphilic block PNBEs.

## Supplementary Material

Supplementary information

Crystal structure data
