# Supplementary material for: Bypassing the lack of reactivity of endo-substituted norbornenes with the catalytic rectification–insertion mechanism
Source: Chem Sci. 2014 Dec 24;6(4):2172–81. doi: 10.1039/c4sc03575e (PMC5701727; doi:10.1039/c4sc03575e)
Supplement: Supplementary file 1 [file SC-006-C4SC03575E-s001.pdf]

Electronic Supplementary Material (ESI) for Chemical Science.
This journal is © The Royal Society of Chemistry 2014

Electronic Supplementary Information

Bypassing the Lack of Reactivity of *endo*-Substituted Norbornenes with the Catalytic Rectification-Insertion Mechanism

Basile Commarieu, Jerome P. Claverie*

Quebec Center for Functional Materials, UQAM, Dept of Chemistry, Succ Centre Ville CP8888, Montreal H3C3P8, Qc., Canada

Table of Content

Experimental Section 6
General considerations 6
Instrumentation: 6
NBE(CO_2ME) (73% *endo*): 6
NBE(CO_2Me) (47% *endo*) 7
NBE(CO_2H) (75% *endo*) 7
NBE(CO_2H) (45% *endo*): 7
NBE(CO_2Me)$_2$ (75% *endo*): 7
NBE(CO_2Me)$_2$ (35% *endo*): 8
NBE(CO_2Me)$_2$ (trans): 8
CA (100% *endo*): 8
CA (35% *endo*): 8
CA (20% *endo*): 8
CA (0% *endo*): 9
NBE(CO_2Me)$_2$ (100% *endo*): 9
NBE(CO_2Me)$_2$ (0% *endo*): 9
NBE(CO_2H)$_2$ (0% *endo*): 9
NBEimide (35% *endo*): 9
NBEimide (5% *endo*): 10
Catalyst 2 11
Crystals of 2 (structure 2s) 11

Compound 2-H_2O ... 11
Catalyst 3 ... 11
Crystals of 3 (structure 3s) ... 11
Compound 3-THF ... 12
Catalyst 4 ... 12
Crystals of 4 (structure 4Ns) ... 12
Preparation of 6 ... 13
Polymerization kinetics of NBE(CO_2Me)$_2$ ... 13
Polymerization kinetics of NBE(CO_2Me)$_2$ ... 13
Polymerization kinetics of NBE(CO_2Me) (Monomer/catalyst=200). ... 14
Polymerization kinetics of NBE in the presence of PNBE(CO_2Me)$_2$. ... 14
Preparation of Polymers (Typical polymerisation): ... 14
Preparation of PCA: ... 15
DFT calculations: ... 15
Figure S1. ^{1}H NMR of 2 in CD_3NO_2 ... 16
Figure S2. COSY of 2 in CD_3NO_2 ... 17
Figure S3. ^{13}C{^{1}H} NMR of 2 in CD_3NO_2 ... 18
Figure S4. DEPT-135 of 2 in CD_3NO_2 ... 19
Figure S5. HSQC of 2 in CD_3NO_2 ... 20
Figure S6 ^{1}H NMR of 3 in CD_3NO_2 ... 21
Figure S7. ^{13}C{^{1}H} NMR of 3 in CD_3NO_2 ... 22
Figure S8. DEPT135 of 3 in CD_3NO_2 ... 23
Figure S9. HSQC of 3 in CD_3NO_2 ... 24
Figure S10. COSY of 3 in CD_3NO_2 ... 25
Figure S11. ^{1}H of 4 in CD_3NO_2 ... 26
Figure S12. ^{13}C{^{1}H} of 4 in CD_3NO_2 ... 27
Figure S13. DEPT135 of 4 in CD_3NO_2 ... 28
Figure S14. HSQC of 4 in CD_3NO_2 ... 29
Figure S15. COSY of 4 in CD_3NO_2 ... 30
Figure S16. ^{1}H NMR of 5 in CD_3NO_2 ... 31
Figure S17. ^{13}C{^{1}H} of 5 in CD_3NO_2 ... 32

Figure S18. DEPT 135 of 5 in CD_3NO_2....33

Figure S19. HMBC of 5 in CD_3NO_2....34

Figure S20. COSY of 5 in CD_3NO_2....35

Figure S21. ^{1}H of 6 in CD_3NO_2 (impurity: dimethyl fumarate, less than 5%).....36

Figure S22. ^{13}C{^{1}H} NMR of 6 in CD_3NO_2 (impurity: dimethyl fumarate, less than 5%).....37

Figure S23. DEPT135 of 6 in CD_3NO_2....38

Figure S24. HSQC of 6 in CD_3NO_2....39

Figure S25. COSY of 6 in CD_3NO_2....40

Figure S26. ^{1}H NMR spectra of the polymerization of NBE(CO_2Me)$_2$ (100% *exo*) with 2 in CD_3NO_2. Top: decrease of the *exo* monomer olefinic resonance at 6.35 ppm, and apparition of the chelated catalyst 5 (not initially present in 2). Center: OCH_3 resonances, indicating the presence of a chelate (protons H_{21}). Bottom, characteristic bridge protons H_{17} of the chelated catalyst 5.....41

Figure S27. (*Experiment A)* ^{1}H NMR spectra of the polymerization of NBE(CO_2Me)$_2$ (100% endo, 10 eq.) with 6 in CD_3NO_2. Top: region of the H_2 protons, indicating that both *endo* and *exo* monomers are inserted. At the end of the polymerization, the chelate disappears and the last *endo* monomer is inserted. Center: region of the C=C double bonds, showing that only traces of *exo* monomer are present (less than 1.5% relative to *endo* monomer). Bottom: increase of the curvature of the baseline with time, indicative of the formation of a polymer (very broad resonance).....42

Figure S28. (*Experiment B)* ^{1}H NMR spectra of the polymerization of NBE(CO_2Me)$_2$ (100% *exo*, 10 eq.) with 6 in CD_3NO_2. Top: region of the H_2 protons. Center: region of the C=C double bonds. Bottom: region of the aliphatic protons.....43

Figure S29. (*Experiment C)* ^{1}H NMR spectrums of NBE(CO_2Me)$_2$ (100% *endo*, 10 eq.) with 5 in CD_3NO_2. Top: region of the H_2 protons, showing that the *endo* H_2 appears gradually. Center: region of the C=C double bonds. Bottom: region of the aliphatic protons.....44

Figure S30. ^{1}H NMR spectra of catalyst 5 vs time. Top: region of the H_{12} protons and H_2, showing that the *endo* H_2 and H_{12} gradually appear to the expense of the exo H_2 and H_{12}. Center: region of the C=C double bonds, showing the rectification of *endo* monomer and the decrease of both monomers concentration. Bottom: region of the aliphatic protons.....45

Figure S31. ^{1}H NMR of catalyst 6 over time, indicating the high stability of the chelate.....46

Figure S32. ^{1}H NMR spectra of the polymerization of NBE(CO_2Me)$_2$ (100% exo, 1 eq.) with 6 in CD_3NO_2. Top: vinyl region, showing that the exo monomer is inserted, but the endo chelate is conserved. Bottom. Rapid disappearance of the H_2 proton in 6 to the benefit of H_2 proton in A, indicative of the high value of $k_{endo,exo}$.....47

Figure S33. Kinetics plots for the reaction of 5 with *endo*-NBE(CO_2Me)$_2$. A. [5] = 0.037 mol/L, [*endo*-NBE(CO_2Me)$_2$]/[5] = 7, B. [5] = 0.041 mol/L, [*endo*-NBE(CO_2Me)$_2$]/[1] = 0.75 C. [5] = 0.031 mol/L,

[*endo*-NBE(CO_2Me)$_2$]/[5] = 1. The value of $k_{exo,endo}$ was determined by taking the ratio of the slope to the catalyst concentration. 48

Figure S34. Kinetics plots for the reaction of 5 with *exo*-NBE(CO_2Me)$_2$. A. [5] = 0.038 mol/L, [NBE(CO_2Me)$_2$]/[5] = 10, B. [5] = 0.030 mol/L, [*exo*-NBE(CO_2Me)$_2$]/[5] = 3 The value of $k_{exo,exo}$ was determined by taking the ratio of the slope to the catalyst concentration. 49

Figure S35. Kinetic plot for the polymerization of NBE(CO_2Me) (73% endo) at 25 ^oC and 50 ^oC by 1 in nitromethane ([NBE(CO_2Me)]/[1] = 200, [NBE(CO_2Me)$_2$] = 0.47 mol/L). The lines correspond to linear fits. The inset corresponds to a zoom of the kinetics at early times (induction period)..............................50

Figure S36. Kinetic plot for the polymerization of NBE at 25 ^oC by 1 in the absence of added polymer, and in the presence of PNBE(CO_2Me)$_2$ (100% exo or 100% endo). Experimental conditions T = 25 ^oC in in deuterated tetrachloroethane, [NBE] = 0.22 mol/L, [NBE]0/[1] = 2000, when added PNBE(CO_2Me)$_2$ is present [C=O] /[1] = 220) 51

Figure S37. ^{1}H NMR of PNBE(CO_2Me)$_2$ prepared with the *exo* monomer and PNBE(CO_2Me)$_2$ prepared with the *endo* monome 52

Figure S38. ^{13}C{^{1}H} NMR of PNBE(CO_2Me)$_2$ prepared with the *exo* monomer and PNBE(CO_2Me)$_2$ prepared with the *endo* monomer (top) and zoom of the carbonyl region (bottom)..................................53

Figure S39. Zoom of the carbonyl region of the ^{13}C spectrum PNBE(CO_2Me)$_2$ prepared with the endo monomer and superimposed deconvolution by three peaks. 54

Figure S40. ^{1}H spectrum PNBE(CO_2Me) (catalyst loading = 0.5 mol%, prepared with NBE(CO_2Me) 75% *endo,* solvent 1,1,2,2-Tetrachloroethane-d2). 55

Figure S41. ^{1}H spectrum PNBE(CO_2H) (catalyst loading = 0.01 mol%, prepared with NBE(CO_2H) 75% *endo,* solvent DMSO-d6). 56

Figure S42. ^{1}H spectrum PNBE(CO_2Me)$_2$ (catalyst loading = 0.01 mol%, prepared with NBE(CO_2Me)$_2$ 75% *endo,* solvent chloroform-d). 57

Figure S43. ^{1}H spectrum PNBECH_2Br (catalyst loading = 0.2 mol%, prepared with NBECH_2Br 86% *endo,* solvent $CDCl_3$) 58

Figure S44. ^{1}H spectrum PNBE(COOH)$_2$ (catalyst loading = 1 mol%, prepared with NBE(COOH)$_2$ *exo,* solvent D_2O)..59

Figure S45. ^{1}H spectrum of PCA (catalyst loading = 0.5 mol%, prepared with CA *exo,* solvent DMSO d6) 60

Figure S46. ^{1}H spectrum of PNBE(imide) (catalyst loading = 0.1 mol%, prepared with NBE imide 90% *exo,* solvent $CDCl_3$)..61

Figure S47 ^{1}H spectrum PNBE(CHO) (catalyst loading = 0.05 mol%, prepared with NBE(CHO) 80% *endo,* solvent $CDCl_3$) 62

Figure S48 DSC thermograms of the polymers (second heating ramp 50 - 300 ^oC), showing the absence of Tg. 63

Table S1. Characteristic bond lengths and bond angles for structures 2-H_2O, 3-THF, 2-s, 3-s and 4-s (for the sake of simplicity, the atom numbering used in this table corresponds to the attached scheme, and not to the atom numbering in the X-Ray structures)....64

Figure S49 ORTEP view of 2-H_2O with 50% probability ellipsoids....65

Figure S50. ORTEP view of 3-THF with 50% probability ellipsoids (for the sake of clarity, the SbF_6 anion has been omitted)....66

Figure S51. ORTEP view of 2s with 50% probability ellipsoids (for the sake of clarity, the four SbF_6 anions and solvent molecules have been omitted)....67

Figure S52. ORTEP view of 3s with 50% probability ellipsoids (for the sake of clarity, the SbF_6 anion and solvent molecules have been omitted)....68

Figure S53. ORTEP view of 4Ns with 50% probability ellipsoids (for the sake of clarity, the SbF_6 anion and solvent molecules have been omitted)....69

References....70

Experimental Section

General considerations: All manipulations that involved air- and/or moisture-sensitive compounds were performed in a nitrogen-filled glove-box. Allylpalladium chloride dimer, palladium dichloride, silver hexafluoroantimonate, norbornene, monomer precursors, NBE(CHO), deuterated solvents for NMR and phenyl silanes were all purchased from Sigma-Aldrich and were stored under nitrogen. Solvents and liquid monomers used for polymerisation were dried over molecular sieves and deoxygenated by bubbling nitrogen.

Instrumentation: ^{1}H, ^{13}C, ^{19}F, COSY, DEPT 145, and HSQC NMR spectra were recorded on Bruker Ultrashield 300 MHz or Varian Inova 600 MHz spectrometers at ambient temperature. ^{1}H and ^{13}C NMR chemical shifts were referenced to the solvent signal. The molecular weight distributions of the polymers were determined by gel permeation chromatography (GPC) using a Viscotek instrument equipped with one PL-Gel mixed A LS 20 μm column, one PL-Gel mixed B LS 10 μm column and one polypore 5 μm column, a Wyatt DSP refractometer and a Wyatt Dawn light scattering detector. For the PNBE(CO_2Me)$_2$ and PNBE(CO_2Me) polymers, elution was performed in THF at 40°C, and all samples were analyzed using a dn/dc of 0.03 and 0.028 respectively. For PNBE(CO_2H), elution was performed at 40°C in DMF containing 1g/L of LiCl (dn/dc = 0.04). PNBE(CO_2H)$_2$ and PCA were analyzed in an aqueous GPC equipped with two Aquagel PAA-M columns from Poly Analytik, using a Wyatt DSP refractometer and a Wyatt Dawn light scattering detector. Elution was performed at 60 °C (0.5 mL/min) using an aqueous buffer ($NaHCO_3$ 0.05 M, $NaNO_3$ 0.1 M, NEt_3 0.02 M, NaN_3 0.05 wt%). All other polymers were analyzed using the same conditions as PNBE(CO_2Me)$_2$, but using dn/dc = 0.04. Crystallographic analysis was performed at the X-ray crystallography laboratory of the University of Delaware, Dept of Chemistry and Biochemistry, by Prof. G. Yapp and Mr G. Andrade. Differential scanning calorimetry measurements (DSC) of solid polymers were performed on a DSC823e (TOPEM modulation) equipped with an FRS5 sample cell, a sample robot, a Julabo FT400 intracooler and an HRS7 sensor from Mettler Toledo. Samples were heated from 50°C to 300°C at a rate of 10 °C/minute and data were analyzed with STAR software. The data associated to the second heated ramp are shown.

NBE(CO_2ME) (73% *endo*): The synthesis of NBE(CO_2Me) was adapted from reference [1]. In short, 269g (3.13 mol, 1.15eq) of methyl acrylate were mixed with 2.1g of hydroquinone (0.019 mol, 0.007eq) and diluted with 100ml of diethyl ether. This solution was cooled down on an iced bath, and freshly cracked cyclopentadiene (180g, 2.73 mol, 1eq) was added dropwise over 60 minutes. When the addition was complete, the reaction was stirred under reflux for 12h. Then diethyl ether was evaporated and the product (a clear liquid) was collected by distillation under vacuum (bp = 110°C at 10mmHg). Yield: 349g (84%). ^{1}H NMR (300MHz, $CDCl_3$) δ 6.10 (m, 1H), 6.04 (m, 0.73H), 5.85(m, 1H), 3.61(s, 1.17H), 3.53(s, 3.16H), 3.11(s, 1.01H), 2.95(s, 0.37H), 2.91-2.78(m, 2.43H), 2.14(m, 0.37H), 1.82(m, 1.50H), 1.46(m, 0.41H), 1.36(m, 2.93H) 1.21(m, 1.08H). The *endo/exo* ratio was determined by ^{1}H NMR analysis: δ 3.61(s, 1.17H, NBE(CO_2ME) (*exo*)), 3.53 (s, 3.16H, NBE(CO_2ME) (*endo*))

NBE(CO_2Me) (47% *endo*): NBE(CO_2Me) (73% *endo*) (50g, 0.33 mol) was heated at 220 ^{o}C for 12 hours under a nitrogen flow. During the heat treatment the solution turned dark-orange. The solution was distilled under vacuum at high temperature to obtain a color-free liquid product containing now 47% endo isomer, as measured by ^{1}H NMR. Yield 47.5g (95%). ^{1}H NMR (300MHz, $CDCl_3$) δ 6.12 (m, 0.92H), 6.06 (m, 1.96H), 5.87(m, 1H), 3.62(s, 3.15H), 3.56(s, 2.77H), 3.14(s, 0.88H), 2.98(s, 1H), 2.91-2.85(m, 2.89H), 2.16(m, 1.06H), 1.85(m, 1.97H), 1.46(m, 1.10H), 1.35(m, 3.91H) 1.23(m, 1H). The *endo/exo* ratio was determined by ^{1}H NMR analysis: δ 3.62(s, 3.15H, NBE(CO_2ME) (*exo*)), 3.56 (s, 2.77H, NBE(CO_2ME) (*endo*))

NBE(CO_2H) (75% *endo*) The synthesis of NBE(CO_2H) was adapted from reference [2]. Acrylic acid (83g, 1.15 mol, 1.2eq) and hydroquinone (1.2 g, 10.9mmol, 0,01 eq) were dissolved in 50mL of ethyl acetate. This solution was cooled in an iced bath, and freshly cracked cyclopentadiene (63g, 0.95 mol, 1eq) was added dropwise over 60 minutes. The reaction was stirred under reflux for 12h. Ethyl acetate was then evaporated and the product was distilled under vacuum (to yield a color-free liquid (bp = 150°C at 5 mmHg). Yield : 100g (76%). The liquid crystallized when left for a few days. ^{1}H NMR (300MHz, $CDCl_3$): δ 6.18(m, 1H), 6.10(m, 0.70H), 5.98(m, 0.97H), 3.21(s, 0.94H), 3.07(s, 0.30H), 2.97(m, 0.98H), 2.89(s, 1.37H), 2.23(m, 0.30H), 1.89(m, 1.33H), 1.51(m, 0.32H), 1.40(m, 2.84H), 1.27(m, 1.07). The *endo/exo* ratio was determined by ^{1}H NMR analysis: δ 3.21(s, 0.94H, NBE(CO_2H) (*endo*)), 3.07(s, 0.30H, NBE(CO_2H) (*exo*))

NBE(CO_2H) (45% *endo*): NBE(CO_2H) (75% endo) (100g, 0.72mol) and hydroquinone (1.6g, 14.5mmol) were heated at 220 ^{o}C for 12 hours under a nitrogen flow. During the heat treatment the solution turned dark-orange. The solution was distilled under vacuum at high temperature to obtain a color-free liquid product containing 55% *exo* isomer and 45% *endo* isomer, as measured by ^{1}H NMR. Yield 50g (0.36mol, 50%) (bp = 150°C at 5 mmHg). The liquid crystallized when left for a few days. ^{1}H NMR (300MHz, $C_2D_2Cl_4$): δ 6.20(m, 1H), 6.12(m, 2.42H), 5.98(m, 1.59H contain $C_2D_2Cl_4$), 3.22(s, 1.02H), 3.08(s, 1.21H), 2.99 (m, 1.04H), 2.92(s, 2.22H), 2.25(m, 1.21H), 1.91(m, 2.35H), 1.49(m, 1.29H), 1.38(m, 4.62H), 1.29(m, 1.10H). The *endo/exo* ratio was determined by ^{1}H NMR analysis: δ 3.22(s, 1.02H, NBE(CO_2H) (*endo*)), 3.08(s, 1.21H, NBE(CO_2H) (*exo*)).

NBE(CO_2Me)$_2$ (75% *endo*): The synthesis of NBE(CO_2Me)$_2$ (75% *endo*) was adapted from reference [3]. Dimethyl maleate (170g, 1.18mol, 1.05eq) and water (9.1g, 0.50 mol, 0.44eq) were mixed in a round bottom flask. This solution was cooled down on an iced bath, and freshly cracked cyclopentadiene (74g, 1.12 mol, 1eq) was added dropwise over 60 minutes. The reaction was stirred under reflux for 12h. The product was distilled under vacuum to yield a color-free liquid (bp = 150°C at 5 mmHg). Yield 174g (0.83mol, 74%). ^{1}H NMR (300MHz, $CDCl_3$): δ 6.19(s, 1H), 6.15(s, 0.30H), 3.60(m, 1.04H), 3.54(m, 3.04H), 3.24(s, 1.04H), 3.10(s 1.02H) 3.03(s 0.31H), 2.56(s, 0.30H) 2.06(m, 0.18H), 1.41(m, 0.71H), 1.29(m, 0.55H). The *endo/exo* ratio was determined by ^{1}H NMR analysis: δ 3.24(s, 1.04H, NBE(CO_2Me)$_2$ (*endo*)), 3.03 (s, 0.31H, NBE(CO_2Me)$_2$ (*exo*))

$NBE(CO_2Me)_2$ (35% *endo*): $NBE(CO_2Me)_2$ (75% *endo*) (60g, 0,285mol) was heated at 220 °C for 12 hours under a nitrogen flow in a round-bottom flask. During the heat treatment the liquid turned dark-orange. The liquid was distilled under vacuum at high temperature to obtain a color-free liquid product containing 65% exo isomer and 35% isomer, as measured by ^{1}H NMR. Yield 50g (0.24 mol, 83%) (bp = 150°C at 5 mmHg). ^{1}H NMR (300MHz, $CDCl_3$): δ 6.17(m, 1H), 6.13(m, 1.94H), 3.57(s, 6.59), 3.52(s, 3.03H), 3.21(s, 1.09H), 3.07(s, 1.07H), 3.00(s, 2.03H), 2.54(m, 2.06H), 2.01(d, 1.07), 1.40(m, 1.65H), 1.27(d, 0.58). The endo/exo ratio was determined by ^{1}H NMR analysis: δ 3.21(s, 1.09H, $NBE(CO_2H)_2$ (*endo*)), 2.54(s, 2.06H, $NBE(CO_2H)_2$ (*exo*)).

$NBE(CO_2Me)_2$ (trans): The synthesis of $NBE(CO_2Me)_2$ (fumarate) was adapted from reference [3]. Dimethyl fumarate (50g, 0.34mol, 1eq) and hydroquinone (1.5g, 13.6mmol) were dissolved in 150mL of ethyl acetate. This solution was cooled in an iced bath, and freshly cracked cyclopentadiene (25g, 0.38 mol, 1.11eq) was added dropwise over 60 minutes. The reaction was stirred under reflux for 12h. Ethyl acetate was then evaporated and the product was distilled under vacuum to yield a color-free liquid (bp = 150°C at 5 mmHg). During distillation, the column was monitored as an impurity (identified as dimethyl fumarate) tended to plug the column. Yield: 42g (0.2mol, 59%). ^{1}H NMR (300MHz, $CDCl_3$): δ 6.22(m, 1H), 6.03(m, 1H), 3.70(s, 3.10H), 3.59,(s, 3.10H), 3.31(t, 1.01H), 3.21(s, 1.01H), 3.07(s, 1.01H), 2.64(m, 0.97H), 1.55(m, 1.05H), 1.42(m, 1.06H).

CA (100% *endo*): Maleic anhydride (95g, 0.97mol) was added to 115g of acetyl acetate. This solution was cooled down on an iced bath, and freshly cracked cyclopentadiene (75g, 1.14 mol) was added dropwise over 60 minutes. When the addition was complete, the reaction was stirred at room temperature for 12 hours. The white precipitate formed was filtered under vaccum, washed 4 times with hexane and dried on an oven at 60 °C for 12 hours. Yield: 145g (0.88 mol, 90%). ^{1}H NMR (300MHz, $CDCl_3$) δ 6.26(s, 1H), 3.56(s, 1.01H), 3.46(s, 1.01H), 1.73(d, 0.55H), 1.57(d, 0.54H).

CA (35% *endo*): The synthesis of CA (35% *endo*) was adapted from reference [5]. In a 250ml Schlenk flask equipped with a condenser, CA (100% *endo*) (50g, 0.30 mol) was heated at 180 °C during 12 hours under a nitrogen flow. The walls of the Schlenk tube were covered with white crystals which were carefully collected, and proved to be CA containing 35% *endo* isomer and 65% *exo* isomer, as measured by ^{1}H NMR. Yield 40g (80%). ^{1}H NMR (300MHz, $CDCl_3$) δ 6.28(s, 1.83H), 6.26(s, 0.94H), 3.54(s, 1H), 3.45(s, 1.04H), 3.40(s, 1.88), 2.96 (s, 1.94), 1.75(d, 0.55), 1.63(d, 1.04), 1.55(d, 0.52), 1.41(d, 1.00). The endo/exo ratio was determined by ^{1}H NMR analysis: δ 3.54(s, 1H, CA (*endo*)), 2.96(s, 1.94H, CA (*exo*)).

CA (20% *endo*): The synthesis of CA (20% *endo*) was adapted from reference [5]. In a 250ml Schlenk flask equipped with a condenser, CA (35% *endo*) (50g, 0.30 mol) was heated at 180 °C during 12 hours under a nitrogen flow. The walls of the Schlenk tube were covered with white crystals which were carefully collected, and proved to be CA containing 20% *endo* isomer and 80% *exo* isomer, as measured by ^{1}H NMR. Yield 35g (70%). ^{1}H NMR (300MHz, $CDCl_3$) δ 6.29(s, 3.92H), 6.27(s, 1.02H), 3.54(s, 1H), 3.47(s, 1.04H), 3.41(s, 4.01H), 2.96(s, 4.01H), 1.73(d, 0.55H), 1.65(d, 2.15H), 1.55(d, 0.55H), 1.42(d, 2.12H). The endo/exo ratio was determined by ^{1}HNMR analysis: δ 3.54(s, 1H, CA (*endo*)), 2.96 (s, 4.01H, CA (*exo*)).

CA (0% *endo*): The synthesis of CA (0% *endo*) was adapted from reference [5]. In a 250ml round bottom flask, CA (20% *endo*) (8.5g, 0.052mol) was solubilised with a minimum of ethyl acetate (~15g ethyl acetate) at ~70 °C. Then the solution was cooled down at a rate of 0.1°C/min (using a programmable heater) in order to obtain pure EXO crystals growing in the solution. When the solution reached room temperature, the crystals were filtered and washed with a low quantity of cold ethyl acetate. Purity greater than 98% as measured by ^{1}H NMR. Yield: 4.7g (0.028 mol, 55%). ^{1}H NMR (300MHz, $C_2D_2Cl_4$) δ 6.19(s, 1H), 3.28(s, 1H), 2.89(s, 1H), 1.53(d, 0.52H), 1.27(d, 0.51H).

NBE(CO_2Me)$_2$ (100% *endo*): The synthesis of NBE(CO_2ME)$_2$ (100% *endo*) was adapted from reference [4]. CA (100% *endo*) (86g, 0.52 mol) and p-TsOH (2.5g, 14.5mmol) were dissolved in MeOH (150mL) in a round-bottom flask and heated at reflux for 24 hours. The methanol was evaporated, and the slurry was diluted with ethyl acetate and extracted with water, dried over $MgSO_4$, filtered and concentrated on a rotary evaporator. The product was distilled under vacuum to yield a color-free liquid (bp = 150°C at 5 mmHg). Yield 80g (0.38mol, 73%). ^{1}H NMR (300MHz, CD_3NO_2) δ 6.19(s, 1H), 3.56(s, 3.42H), 3.35(s, 1.15H), 3.12(s, 1.09H), 1.41(s, 1.20H). ^{1}H NMR (300MHz, CDCl3) δ 6.21(s, 1H), 3.57(s, 2.94H), 3.26(s, 0.99H), 3.12(s, 0.96H), 1.42(d, 0.5H), 1.31(d, 0.5H).^{13}C NMR (75 MHz, $CDCl_3$) δ 172.68, 134.74, 51.28, 48.51, 47.89, 46.14.

NBE(CO_2Me)$_2$ (0% *endo*): The synthesis of NBE(CO_2ME)$_2$ (0 % *endo*) was adapted from reference [4]. CA (0% *endo*) (34g, 0.21 mol) and p-TsOH (1g, 5.8 mmol) were dissolved in MeOH (100 mL) in a round bottom flask and heated at reflux for 24 hours. The methanol was evaporated, and the slurry was diluted with ethyl acetate and extracted with water, dried over $MgSO_4$, filtered and concentrated on a rotary evaporator. The product was distilled under vacuum to yield a color-free liquid (bp = 150°C at 5 mmHg). Care was taken to monitor the column and the condenser as the product tended to crystallize and plug the column. Yield 34g (0.16 mol, 76%). The liquid was fully crystallized a few hours after distillation. ^{1}H NMR (300MHz, CD_3NO_2) δ 6.25(s, 1H), 3.61(s, 3.13H), 3.03(s, 1.03H), 2.63(s, 1.04H), 2.02(s, 0.52H), 1.43(s, 0.56H). ^{1}H NMR (300MHz, CDCl3) δ 6.17(s, 1H), 3.62(s, 2.97H), 3.06(s, 0.98H), 2.59(s, 0.99H), 2.06(d, 0.52H), 1.49(d, 0.54) ^{13}C NMR (75 MHz, $CDCl_3$) δ 173.96, 137.94, 51.77, 47.22, 45.52, 45.39.

NBE(CO_2H)$_2$ (0% *endo*): In a 250ml Schlenk flask equipped with a condenser, CA (0% *endo*) (20g, 0.122 mol, 1eq) were dissolved in 100g of THF and water (10g, 0.555mol, 4.5eq) was added. Then the reaction was stirred under reflux for 6h. The solvent was evaporated under vacuum to obtain white dry powder. Yield (20.9g 0.115 mol, 94%). ^{1}H NMR (300MHz, acetone d) δ 6.23(s, 1.05H), 3.02(s, 1H), 2.59(s, 1H), 2.14(d, 052H), 1.35(d, 0.55)

NBEimide (35% *endo*): In a 250ml Schlenk flask equipped with a condenser, CA (35% *endo*) (30g, 0.183mol, 1eq) was dissolved in 150g of THF. This solution was cooled in an iced bath, and allyl amine (15g, 0.262mol, 1.43eq) was added dropwise over 60 minutes (reaction very exothermic). A white precipitate was immediately formed. When the addition was complete, the reaction was stirred at room temperature for 2 hours. The solution was concentrated on a rotary evaporator to yield an off-white solid. The product was distilled under vacuum to yield a color-free liquid (bp=200°C at 5 mmHg). The high temperature induces the imidation reaction conducting to formation of water which was trapped in an dry-

ice trap connected to the vacuum outlet. Care was taken to monitor the column and the condenser as the product tended to crystallize and plug the column. Occasionally, the column and refrigerant walls were heated to avoid plugging. Yield 31g (0.152mol, 83%). ^{1}H NMR (300MHz, $CDCl_3$) δ 6.19 (s, 1.01H), 5.99(s, 0.54H), 5.69(m, 0.51H), 5.54(m, 0.26), 5.07(m, 1.58H), 3.98(d, 1.04H), 3.82(d, 0.58H), 3.29(m, 0.57H) , 3.18 (m, 1.55H), 2.60(d, 1.00H), 1.62(m, 0.31H), 1.47(m, 0.30H), 1.39(m, 0.53H), 1.16(m, 0.53H). The endo/exo ratio was determined by ^{1}H NMR analysis: δ 6.19(s, 1.01H, NBE(imide) (*exo*)), 5.99(s, 0.54H, NBE(imide) (*endo*)).

NBEimide (5% *endo*): In a 250ml Schlenk flask equipped with a condenser, CA (20% *endo*) (30g, 0.183 mol, 1eq) was dissolved in 150g of THF. This solution was cooled in an iced bath, and allyl amine (15g, 0.262mol, 1.43eq) was added dropwise over 60 minutes (reaction very exothermic). A white precipitate was immediately formed. When the addition was complete, the reaction was stirred at room temperature for 2 hours. The solution was vacuum-filtered in order to separate the solid from the solution. We believe that the solid is enriched in *endo* adduct whereas the liquid contains the *exo* adduct. The solution was cooled in the fridge, leading to the precipitation of *endo* rich adducts. After separation, the solution was concentrated on a rotary evaporator to yield a white solid. Yield 16g. The product was distilled under vacuum to yield a color-free liquid (bp=200°C at 5 mmHg). The high temperature induces the imidation reaction conducting to formation of water which was trapped in an dry-ice trap connected to the vacuum outlet. Care was taken to monitor the column and the condenser as the product tended to crystallize and plug the column. To avoid the crystallization on the column walls the column was heated at 60°C. Yield 15g (0.074 mol, 40%). ^{1}H NMR (300MHz, $CDCl_3$) δ 6.26(s, 0.95H), 6.06(s, 0.05H), 5.73(m, 0.47H), 5.20(m, 1.01H), 4.03(d, 0.97H), 3.91(d, 0.05H), 3.35(s, 0.05H), 3.25(s, 1.00H), 2.67(s, 0.94H), 1.66(m, 0.4H),1.49(d, 0.56H), 1.19 (d, 0.50H). The *endo/exo* ratio was determined by ^{1}H NMR analysis: δ 6.26(s, 0.95H, NBE(imide) (*exo*)), 6.06(s, 0.05H, NBE(imide) (*endo*)).

NBE(CH_2OH) (82% *endo*): The synthesis of NBE(CH_2OH) was adapted from reference [6]. In short, 40 mL of dicyclopentadiene (39 g, 0.30 mol, 2 eqs), 34 mL of allyl alcohol (29 g, 0.5 mol, 3.5 eqs) were introduced in a pressurized reactor at 210 °C for 1 hour (yield : 21 g). ^{1}H NMR^{1}H NMR (300 MHz, $CDCl_3$) δ 6.08 (dd, J = 5.7, 3.0 Hz, 1H), 6.02 (td, J = 6.2, 5.6, 3.0 Hz, 0.4 H), 5.91 (dd, J = 5.7, 2.9 Hz, 1H), 3.62 (dd, J = 10.6, 6.4 Hz, 0.4 H), 3.45 (dd, J = 10.6, 8.8 Hz, 0.4H), 3.31 (dd, J = 10.4, 6.6 Hz, 1H), 3.16 (dd, J = 10.3, 8.7 Hz, 2H), 2.88 (s, 1H), 2.81 – 2.63 (m, 2H), 2.24 (dtt, J = 13.2, 9.2, 3.9 Hz, 1H), 1.76 (ddd, J = 11.4, 9.2, 3.8 Hz, 1H), 1.63 – 1.51 (m, 0H), 1.46 – 1.34 (m, 1H), 1.33 – 1.11 (m, 2H), 1.06 (dq, J = 11.6, 4.0, 3.4 Hz, 0.4H), 0.46 (ddd, J = 11.5, 4.5, 2.6 Hz, 1H).

NBE(CH_2Br) (86% *endo*): The synthesis of NBE(CO_2Me) is described on this reference [7]. In short, 30 mL of dicyclopentadiene (29.4 g, 0.22 mol, 2 eqs), 46 mL of allyl bromide (64g, 0.55 mol, 5 eqs) and 150 mg of hyroquinone were introduced in a pressurized reactor at 130 °C for 15 hours (yield : 29 g). ^{1}H NMR (300 MHz, $CDCl_3$) δ 6.21 (dd, J = 5.8, 3.1 Hz, 1H), 6.10 (dd, J = 4.9, 2.9 Hz, 0H), 6.00 (dd, J = 5.8, 2.9 Hz, 1H), 3.52 – 3.32 (m, 0H), 3.21 (dd, J = 9.6, 6.8 Hz, 1H), 3.05 (d, J = 9.6 Hz, 1H), 3.02 – 2.95 (m, 1H), 2.88 (dq, J = 3.6, 1.8 Hz, 1H), 2.53 (dtdd, J = 13.0, 9.7, 5.7, 3.3 Hz, 1H), 1.95 (ddd, J = 11.9, 9.1, 3.8 Hz, 1H), 1.50 (dq, J = 8.3, 2.1 Hz, 1H), 1.31 (dt, J = 8.3, 1.6 Hz, 1H), 0.60 (ddd, J = 11.8, 4.4, 2.7 Hz, 1H).

Catalyst 2 In the drybox, a stock solution of catalyst **1** was prepared by adding $[PdCl(C_3H_5)]_2$ (30mg , 82 µmol, 164 µmol of Pd, 1 eq) and $AgSbF_6$ (69mg, 201 µmol, 1.22 eq) to deuterated nitromethane (5g, [**1**]=32.8 µmol/g). The yellow solution was filtered with a 0.22 µm syringe filtrer in order to remove the AgCl precipitate and the excess $AgSbF_6$. In a separate vial, $NBE(CO_2Me)_2$ (100% *exo*) (0.1g, 0.476 mmol, 1 eq) was mixed with deuterated nitromethane (0.9g, $[NBE(CO_2Me)_2]$= 0.48 mmol/g). In a NMR tube, part of the stock solution of **1** (0.73g, 23.9 µmol, 1eq) was mixed with part of the monomer solution (50mg, 23.8 µmol, 1eq). The tube was sealed, stirred and immediately analyzed by NMR, to yield a pale yellow catalytic solution. ^{1}H NMR (300 MHz, CD_3NO_2) δ 6.09 (H11, m, 1H), 4.75 (H12a, d, *J* = 14.7 Hz, 1H), 4.69 (H12s, d, *J* = 8.3 Hz, 1H), 3.93 (H2, d, *J* = 7.9, 1H), 3.70 (H9, s, 6H), 2.86 (H1+H4, s, 2H), 2.7-2.5 (H10, m, 2H), 2.32 (H5/6, s, 1H), 2.22 (H6/5, s, 1H), 2.15 (H7a, d, *J* = 11.0 Hz, 1H), 2.06 (H7b, d, *J* = 11.0 Hz, 1H), 1.96 (H3, dd, *J* = 7.4, 5.6 Hz, 1H). ^{13}C NMR (75 MHz, CD_3NO_2) δ 174.76, 173.25, 111.50, 103.23, 78.93, 68.84, 63.09, 52.80, 52.66, 50.40, 49.67, 47.50, 46.68, 46.60, 37.49, 32.92.

Crystals of 2 (structure 2s) In the drybox, a stock solution of catalyst **1** was prepared by adding $[PdCl(C_3H_5)]_2$ (52.5mg , 143.5 µmol, 287 µmol of Pd, 1 eq) and $AgSbF_6$ (120.75mg, 354.4 µmol, 1.23 eq) to dichloromethane (5g, [**1**]=57.4 µmol/g). The yellow solution was filtered with a 0.22 µm syringe filtrer in order to remove the AgCl precipitate and the excess $AgSbF_6$. In a separate vial, $NBE(CO_2Me)_2$ (100% exo) (0.1g, 0.476 mmol, 1 eq) was mixed with deuterated tetrachloroethane (0.9g, $[NBE(CO_2Me)_2]$= 0.476 mmol/g). In a vial, part of the stock solution of monomer (0.6g, 0.286 mmol, 1eq) was mixed with the catalyst solution. The vial was stirred to yield a pale yellow catalytic solution. Crystals were obtained by slow evaporation of the solvent from the rest of the solution.

Compound 2-H_2O This compound was prepared in the same manner as 2, but by replacing deuterated tetrachloroethane by a mixture of deuterated nitreomethane:water (9.5:0.5 v:v). Crystals were obtained by slow evaporation of the solvent.

Catalyst 3 In the drybox, a stock solution of catalyst **1** was prepared by adding $[PdCl(C_3H_5)]_2$ (30mg , 82 µmol, 164 µmol of Pd, 1 eq) and $AgSbF_6$ (69mg, 201 µmol, 1.22 eq) to deuterated nitromethane (5g, [**1**]= 32.8 µmol/g). The yellow solution was filtered with a 0.22 µm syringe filtrer in order to remove the AgCl precipitate and the excess $AgSbF_6$. In a separate vial, $NBE(CO_2Me)_2$ (100% *endo*) (0.1g, 0.476 mmol, 1 eq) was mixed with deuterated nitromethane (0.9g, $[NBE(CO_2Me)_2]$= 0.48 mmol/g). In a NMR tube, part of the stock solution of **1** (0.73g, 23.9 µmol, 1eq) was mixed with part of the monomer solution (50mg, 23.8umol, 1eq). The tube was sealed, stirred and immediately analyzed by NMR, to yield a pale yellow catalytic solution. ^{1}H NMR (300 MHz, CD_3NO_2) δ 6.07 (H11, tt, *J* = 14.5, 7.2 Hz, 1H), 4.82 (H12a, d, *J* = 14.7 Hz, 1H), 4.70 (H12s, d, *J* = 8.2 Hz, 1H), 4.33 (H2, d, *J* = 6.2 Hz, 1H), 3.73 (H9, s, 6H), 3.25 (H5 or H6, dd, *J* = 11.5, 4.4 Hz, 1H), 2.91 (H5 or H6, dd, *J* = 11.6, 4.1 Hz, 1H), 2.78 – 2.40 (H10, m, 2H), 2.34 (H7b + H3 + H1 + H4, m, 45H), 1.55 (H7a, d, *J* = 11.3 Hz, 1H). ^{13}C NMR (75 MHz, CD_3NO_2) δ 174.15, 110.46, 78.78, 66.90, 52.52, 52.35, 48.10, 47.59, 46.62, 46.40, 43.99, 37.25, 35.96.

Crystals of 3 (structure 3s) In the drybox, a stock solution of catalyst **1** was prepared by adding $[PdCl(C_3H_5)]_2$ (52.5mg , 143.5 µmol, 287 µmol of Pd, 1 eq) and $AgSbF_6$ (120.75mg, 354.4 µmol, 1.23

eq) to deuterated nitromethane (5g, [**1**]=57.4 µmol/g). The yellow solution was filtered with a 0.22 µm syringe filtrer in order to remove the AgCl precipitate and the excess $AgSbF_6$. In a separate vial, $NBE(CO_2Me)_2$ (100% endo) (0.1g, 0.476 mmol, 1 eq) was mixed with deuterated nitromethane (0.9g, [$NBE(CO_2Me)_2$]= 0.476 mmol/g). In a vial, part of the stock solution of monomer (0.6g, 0.286 mmol, 1eq) was mixed with the catalyst solution. The vial was stirred to yield a pale yellow catalytic solution. Crystals were obtained by slow evaporation of the solvent from the rest of the solution.

Compound 3-THF In the drybox, a stock solution of catalyst **1** was prepared by adding $[PdCl(C_3H_5)]_2$ (52.5mg , 143.5 µmol, 287 µmol of Pd, 1 eq) and $AgSbF_6$ (120.75mg, 354.4 µmol, 1.23 eq) to DCM (5g, [**1**]=57.4 µmol/g). The yellow solution was filtered with a 0.22 µm syringe filtrer in order to remove the AgCl precipitate and the excess $AgSbF_6$. In a separate vial, $NBE(CO_2Me)_2$ (100% endo) (0.1g, 0.476 mmol, 1 eq) was mixed with THF (0.9g, [$NBE(CO_2Me)_2$]= 0.476 mmol/g). In a vial, part of the stock solution of monomer (0.6g, 0.286 mmol, 1eq) was mixed with the catalyst solution. The vial was stirred to yield a pale yellow catalytic solution. Crystals were obtained by slow evaporation of the solvent from the rest of the solution.

Catalyst 4 In the drybox, a stock solution of catalyst **1** was prepared by adding $[PdCl(C_3H_5)]_2$ (30mg , 82 µmol, 164 µmol of Pd, 1 eq) and $AgSbF_6$ (69mg, 201 µmol, 1.22 eq) to deuterated nitromethane (5g, [**1**]=32.8 µmol/g). The yellow solution was filtered with a 0.22 µm syringe filtrer in order to remove the AgCl precipitate and the excess $AgSbF_6$. In a separate vial, $NBE(CO_2Me)_2$ (fumarate) (0.1g, 0.476 mmol, 1 eq) was mixed with deuterated nitromethane (0.9g, [$NBE(CO_2Me)_2$]= 0.48 mmol/g). In a NMR tube, part of the stock solution of **1** (0.73g, 23.9 µmol, 1eq) was mixed with part of the monomer solution (50mg, 23.8umol, 1eq). The tube was sealed, stirred and immediately analyzed by NMR, to yield a pale yellow catalytic solution. ^{1}H NMR (300 MHz, CD_3NO_2): δ 6.16 – 5.97 (H11, m, 1H), 4.80 (H12aN, d, *J* = 8.6 Hz, 1H), 4.74 (H12aX, d, *J* = 6.8 Hz, 1H), 4.69 (H12sN, d, *J* = 2.2 Hz, 1H), 4.66 (H12sX, d, *J* = 2.5 Hz, 1H), 3.80 (H2 N&X + H9 N&X, m, 7H), 3.20 (H1N, dd, *J* = 5.4, 4.3 Hz, 1H), 3.10 – 2.98 (H4X, dd, *J* = 4.9, 4.3 Hz, 1H), 2.80 (H1X + H4N, dd, *J* = 7.0, 6.7 Hz, 2H), 2.76 – 2.48 (H10 N&X, m, 4H), 2.43 (H5 or H6 N&X dd, *J* = 10.8, 6.5 Hz, 2H), 2.37 (H5 or H6 N&X d, *J* = 5.9 Hz, 2H), 2.21 (H7b, N&X, dd, *J* = 13.6, 4.0 Hz, 1H), 2.10 – 1.89 (H3 N&X, m, 2H), 1.63 (H7a N&X, d, *J* = 10.8 Hz, 1H). ^{13}C NMR (75 MHz, CD_3NO_2) δ 175.47, 175.31, 174.94, 111.67, 111.22, 79.08, 68.44, 64.99, 63.10, 53.60, 53.51, 53.25, 50.31, 49.75, 49.04, 48.73, 48.04, 47.95, 47.79, 46.68, 46.00, 43.47, 37.73, 37.45, 35.03, 34.78.

Crystals of 4 (structure 4Ns) In the drybox, a stock solution of catalyst **1** was prepared by adding $[PdCl(C_3H_5)]_2$ (52.5mg , 143.5 µmol, 287 µmol of Pd, 1 eq) and $AgSbF_6$ (120.75mg, 354.4 µmol, 1.23 eq) to CD_3NO_2 (5g, [**1**]=57.4 µmol/g). The yellow solution was filtered with a 0.22 µm syringe filtrer in order to remove the AgCl precipitate and the excess $AgSbF_6$. In a separate vial, $NBE(CO_2Me)_2$ (100% endo) (0.1g, 0.476 mmol, 1 eq) was mixed with deuterated nitromethane (0.9g, [$NBE(CO_2Me)_2$]= 0.476 mmol/g). In a vial, part of the stock solution of monomer (0.6g, 0.286 mmol, 1eq) was mixed with the catalyst solution. The vial was stirred to yield a pale yellow catalytic solution. Crystals were obtained by slow evaporation of the solvent from the rest of the solution.

Preparation of 5 In the drybox, a stock solution of monomer was prepared by mixing NBE(CO_2Me)$_2$ (100% endo) (0.05g, 0.238 mmol) in deuterated nitromethane (0.95g, [NBE(CO_2Me)$_2$]= 0.238 mmol/g). In a NMR tube containing the catalyst **2** solution (23.9 µmol, 1eq), 0.1 g of the NBE(CO_2Me)$_2$ stock solution (0.1g, 23.8 µmol, 1eq) was added. The tube was sealed, stirred and immediately analyzed by NMR. ^{1}H NMR (300 MHz, CD_3NO_2) δ 7.03 (H13+14, s, 2H), 6.09 (H11, m, 1H), 4.72 (H12a, d, *J* = 14.4 Hz, 1H), 4.60 (H12s, d, *J* = 8.5 Hz, 1H), 3.89 (H2, d, *J* = 7.9, 1H), 3.81 (H21, s, 6H) 3.75 (H15+16, s, 2H), 3.69 (H9, s, 6H), 3.43 (H18+19, s, 2H), 2.85 (H1+H4, s, 2H), 2.7-2.5 (H10, m, 2H), 2.31 (H5+6, s, 2H), 2.2-2.1 (H7, m, 2H), 2.1-2.0 (H3, m, 1H), 1.71 (H17, s, 1H). ^{13}C NMR (75 MHz, CD_3NO_2) δ 177.52, 174.67, 173.13, 135.72, 111.94, 78.22, 68.44, 53.83, 52.67, 52.57, 51.64, 50.47, 49.63, 48.47, 47.79, 46.83, 46.68, 37.60, 32.97.

Preparation of 6 In the drybox, a stock solution of monomer was prepared by mixing NBE(CO_2Me)$_2$ (100% endo) (0.05g, 0.238 mmol) in deuterated nitromethane (0.95g, [NBE(CO_2Me)$_2$]= 0.238 mmol/g). In the a NMR tube containing the catalyst **3** solution (23.9 µmol, 1eq), 0.1 g of the NBE(CO_2Me)$_2$ stock solution (0.1g, 23.8 µmol, 1eq) was added. The tube was sealed, stirred and immediately analyzed by NMR. ^{1}H NMR (300 MHz, CD_3NO_2) δ 7.22 (H13+14, s, 2H), 6.09 (H11, m, 1H), 4.77 (H12a, d, *J* = 14.4 Hz, 1H), 4.55 (H12s, d, *J* = 8.5 Hz, 1H), 4.34 (H2, d, *J* = 6.56 Hz, 1H), 3.80 (H21, s, 6H), 3.72 (H9, s, 6H), 3.68 (H15+16, s, 2H), 3.44 (H18+19, s, 2H), 3.29 (H5+H6, dd, *J* = 11.5, 4.4 Hz, 1H), 2.94 (H5 or H6, dd, *J* = 11.5, 4.4 Hz, 1H), 2.7-2.6 (H10, m, 2H), 2.40-2.37 (H1+H3+ H4+H7b, m, 4H), 1.72 (H17, dd, *J* = 9.7, 7.7 Hz, 2H), 1.55 (H7b, d, *J* = 12.0, 1H). ^{13}C NMR (75 MHz, CD_3NO_2) δ 177.11, 173.84, 135.46, 130.79, 111.71, 77.19, 65.22, 53.47, 52.76, 52.18, 51.93, 51.61, 49.28, 48.18, 47.90, 47.44, 46.43, 46.31, 44.09, 37.22, 35.71.

Polymerization kinetics of NBE(CO_2Me)$_2$ (Monomer/catalyst=10). A stock solution of catalyst **1** was prepared by adding [PdCl(C_3H_5)]$_2$ (30mg, 82 µmol, 164 µmol of Pd, 1 eq) and $AgSbF_6$ (69mg, 201 µmol, 1.22 eq) to deuterated nitromethane (5g, [**1**]=32.8 µmol/g). Then the solution was filtered with a 0.22µm filter to remove the AgCl precipitate. A transparent yellow catalytic solution was obtained. In a separate vial, NBE(CO_2Me)$_2$ (0.5g, 2.38mmol) was diluted with deutereted nitromethane (0.5g, [NBE(CO_2Me)$_2$]= 2.38 mmol/g). In a NMR tube, part of the stock solution of **1** (0.73g, 23.9 µmol, 1eq) was mixed with part of the monomer solution (0.1g, 238 µmol, 10eq). The tube was sealed, stirred and immediately analyzed by NMR. Monomer conversion was measured at regular intervals by ^{1}H NMR.

Polymerization kinetics of NBE(CO_2Me)$_2$ (Monomer/catalyst=100). In a 50ml flask, [PdCl(C_3H_5)]$_2$ (100mg, 273 µmol, 546 µmol of Pd, 1 eq) and AgSbF6 (230mg, 669 µmol 1.22 eq) were solubilized in nitromethane (11.4 g, 47.81 µmol/g). The temperature was set at 50°C then when the solid yellow catalyst was fully solubilized, NBE(CO_2Me)$_2$ (11.42g, 54.4 mmol, 100eq) was added under vigorous stirring. Monomer conversion was measured at regular intervals by withdrawing 1g aliquots (23.9 µmol of catalyst **1**, 0.0437eq) which were added to a vial containing 10.3mg of phenylsilane (95µmol, 0.174eq, phenylsilane:catalyst= 4:1 mol:mol) in order to kill the catalyst. The reaction of the Pd naked catalyst with phenyl silane leads to a violent reaction and results in the formation of palladium black. The polymer was

precipitated in diethyl ether (10ml) and washed 5 times with diethyl ether. It was then filtered and dried under vacuum at 80°C overnight.

Polymerization kinetics of NBE(CO_2Me) (Monomer/catalyst=200). In a 50ml flask, [PdCl(C_3H_5)]$_2$ (138mg, 377 µmol, 754 µmol of Pd, 1 eq) and AgSbF6 (317mg, 924 µmol 1.22 eq) were solubilized in nitromethane (22.9, 33.01 µmol/g). The temperature was set at 50°C then when the solid yellow catalyst was fully solubilized, NBE(CO_2Me) (22.84g, 150.2 mmol, 200eq) was added under vigorous stirring. Monomer conversion was measured at regular intervals by withdrawing 1g aliquots (16.50 µmol of catalyst **1**, 0.0219eq) which were added to a vial containing 5.15 mg of phenylsilane phenylsilane (47.5µmol, 0.087eq, phenylsilane:catalyst= 4:1 mol:mol) in order to kill the catalyst. The reaction of the Pd naked catalyst with phenyl silane leads to a violent reaction and results in the formation of palladium black. The polymer was precipitated in diethyl ether (10ml) and washed 5 times with diethyl ether. It was then filtered and dried under vacuum at 80°C overnight.

Polymerization kinetics of NBE in the presence of PNBE(CO_2Me)$_2$. A stock solution of palladium complex was prepared by adding [PdCl(C_3H_5)]$_2$ (2.0 mg, 5.5 µmol, 11 µmol of Pd) to DCM (1g) and chlorobenzene (9g). The Pd concentration in this solution was 1.1 µmol/g. A stock solution of co-catalyst was prepared by adding $AgSbF_6$ (4.6mg, 13.4 µmol) to chlorobenzene (10.0g, [$AgSbF_6$] = 1.34 µmol/g). A stock solution of monomer was prepared by adding NBE (20.0mg, 0.212 mmol) into deuterated tetrachloroethane (1.1g, [NBE]=0.193 mmol/g). A stock solution of PNBE(CO_2Me)$_2$ was prepared by adding 10mg of polymer (10mg, 47.6 µmol) in deuterated tetrachloroethane (1g, [PNBE(CO_2Me)$_2$]=47.6 µmol/g). In an NMR tube were consecutively added the Pd complex solution (0.1g, 0.11 µmol, 1eq) the polymer solution(0.23g, 10.948 µmol, 100eq) and the monomer solution (1.1g, 0.212 mmol, 2000eq) and then the tube was stirred. Immediately prior analysis, the co-catalytic solution (0.1g, 0,134 µmol, 1.22 eq) was added to trigger the polymerization. Monomer conversion was measured at regular intervals by ^{1}H NMR. When the polymerization was performed in the absence of added PNBE(CO_2Me)$_2$ (control experiment), 0.1g of solvent was added instead, so that all experiments were performed with an initial concentation of [NBE]=0.138 mmol/g and [NBE]/[**1**]=2000.

Preparation of Polymers (Typical polymerisation):

With solvent: A stock solution of catalyst **1** was prepared by adding [PdCl(C_3H_5)]$_2$ (100mg, 273 µmol, 546 µmol of Pd, 1 eq) and $AgSbF_6$ (230mg, 669 µmol, 1.22 eq) to nitromethane (10g, [**1**]=54.6 µmol/g). Then the solution was filtered using a 0.22µm filter to remove the AgCl precipitate. A transparent yellow catalytic solution was obtained. A vial was loaded with part of the stock solution of **1** (0.875g, 47.78 µmol, 1eq) nitromethane (0.125g) and NBE(CO_2Me)$_2$ (1g, 4.761mmol, 100eq). The solution was heated at 70°C under vigorous stirring for 24h. The polymer was precipitated by adding diethyl ether (10ml) and was washed 5 times with diethyl ether. It was then filtered and dried under vacuum at 80°C overnight.

Without solvent: A stock solution of catalyst **1** was prepared by adding [PdCl(C_3H_5)]$_2$ (100mg, 273 µmol, 546 µmol of Pd, 1 eq) and $AgSbF_6$ (230mg, 669 µmol, 1.22 eq) to nitromethane (3ml, [**1**]=182 µmol/ml). Then the solution was filtered using a 0.22µm filter to remove the AgCl precipitate. A transparent yellow catalytic solution was obtained. A vial was loaded with NBE(CO_2H) (2g, 14.49mmol, 10 000eq). Using a

microsyringe, 10µL the stock solution of **1** (7.8µL, 1.42 µmol, 1eq) was added and the vial was heated at 70°C under vigorous for 24h. The polymer was precipitated by adding diethyl ether (10ml) and was washed 5 times with diethyl ether. It was then filtered and dried under vacuum at 80°C overnight.

Preparation of PCA:

The polymer of CA, PCA could not be prepared without solvent as the monomer is a solid. A stock solution of catalyst **1** was prepared by adding $[PdCl(C_3H_5)]_2$ (100mg, 273 µmol, 546 µmol of Pd, 1 eq) and $AgSbF_6$ (230mg, 669 µmol, 1.22 eq) to nitromethane (10g, [**1**]=54.6 µmol/g). Then the solution was filtered using a 0.22µm filter to remove the AgCl precipitate. A transparent yellow catalytic solution was obtained. A vial was loaded with CA (1g, 6.09mmol, 200 eq) and nitromethane (5.74g,) and heated at 70°C to solubilize CA. Then the vial was loaded with part of the stock solution of **1** (0.56g, 30.78 µmol, 1eq, [CA]=0.87mmol/g). The solution was heated at 70°C under vigorous stirring for 24h. The precipitated polymer in nitromethane was separated by centrifugation and was washed 5 times with diethyl ether. It was then filtered and dried under vacuum at 80°C overnight. The polymer $PNBE(CO_2H)_2$ was prepared following the same protocol.

DFT calculations:

Calculations were performed using a DFT approach, using the B3LYP functional, and the 6-311g(d,p) basis set. Both *endo* and *exo* $NBE(CO_2Me)_2$ structures were minimized using the Gaussian software[8] which includes the three-parameter gradient-corrected exchange functional of Becke[9] and the correlation functional of Lee, Yang, and Parr, which includes both local and nonlocal terms.[10]

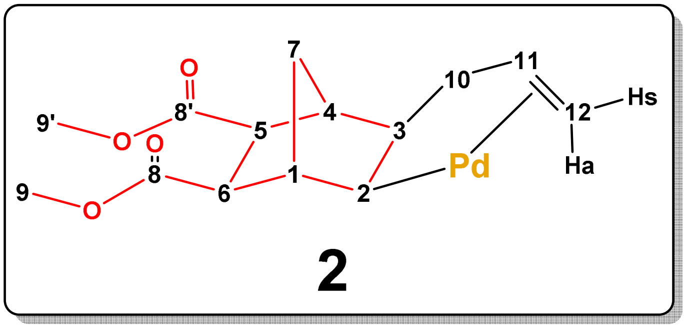

2

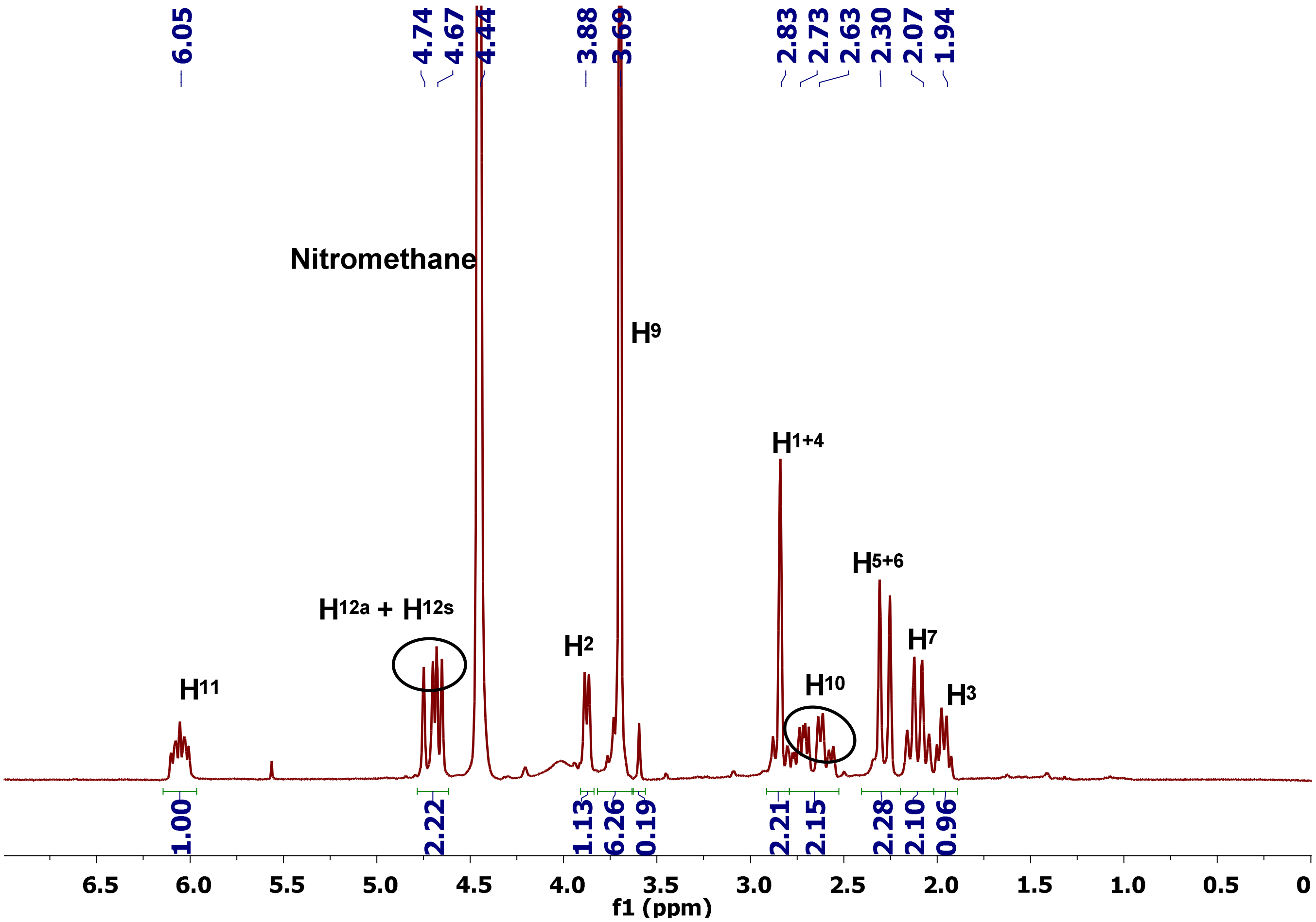

Figure S1. ^{1}H NMR of **2** in CD_3NO_2

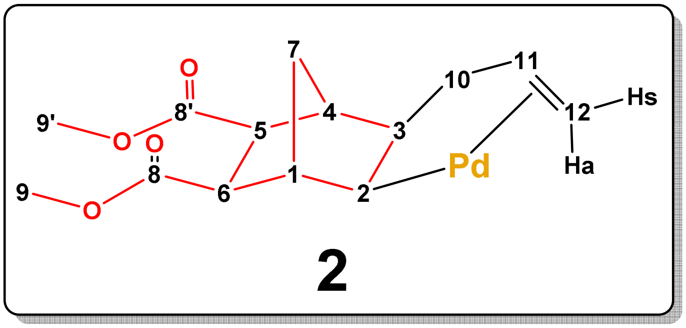

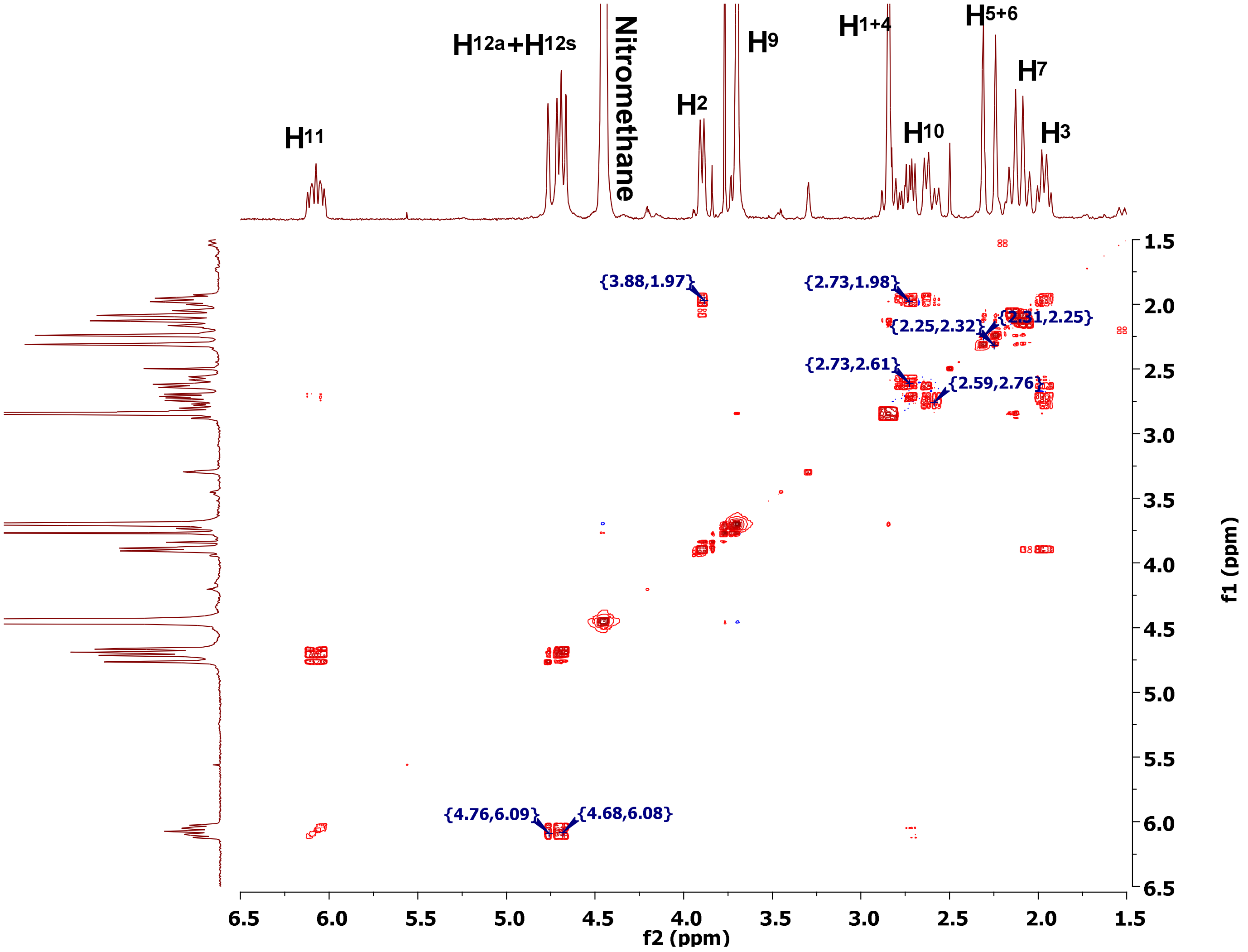

<u>Figure S2.</u> COSY of **2** in CD_3NO_2

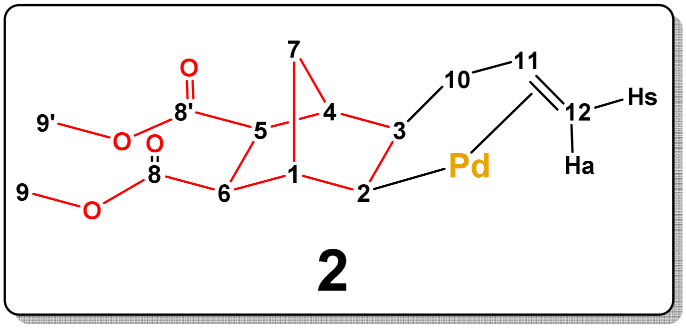

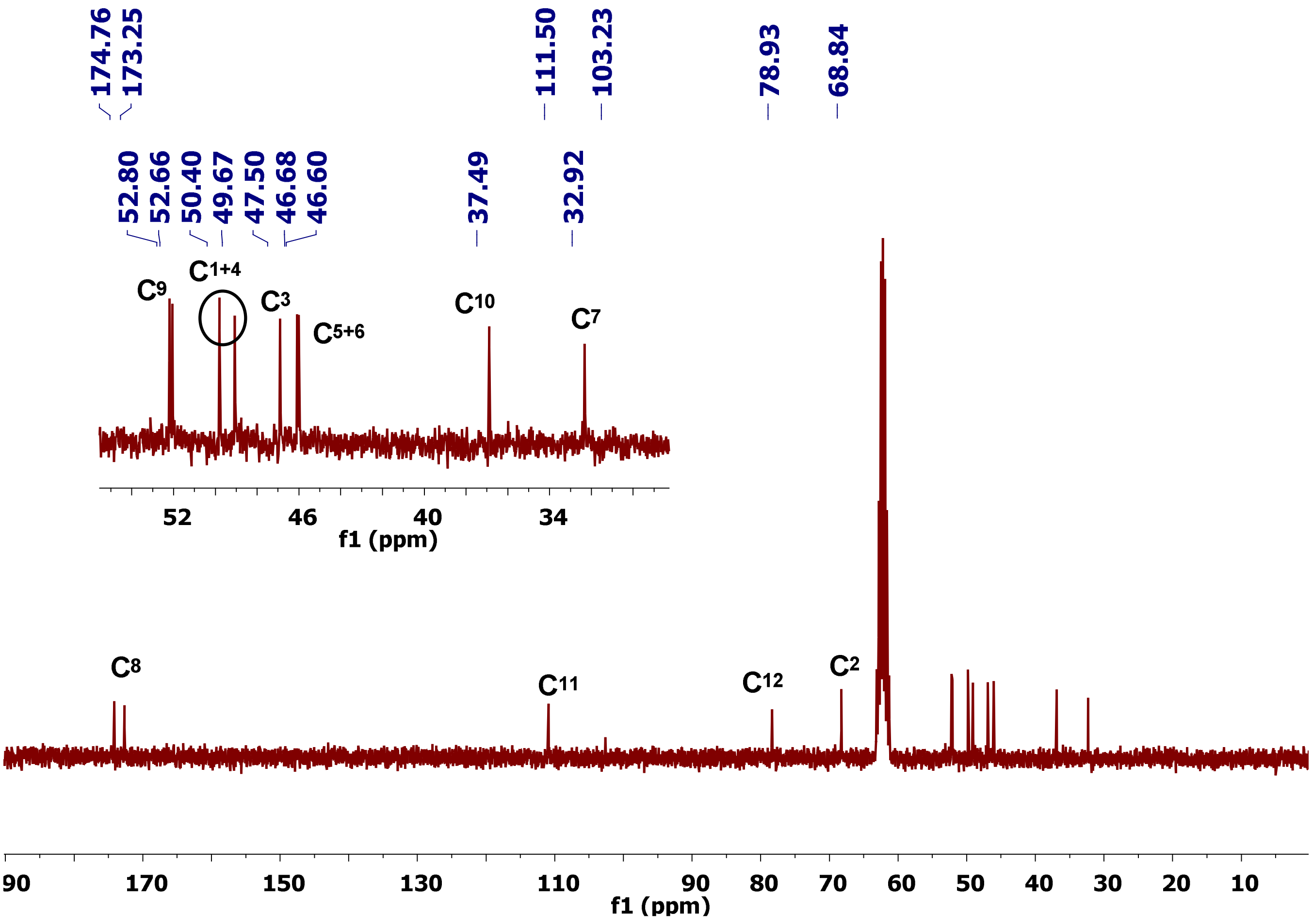

<u>Figure S3.</u> $^{13}C\{^{1}H\}$ NMR of **2** in CD_3NO_2

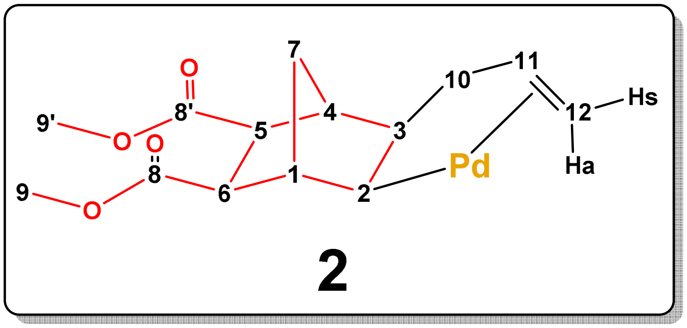

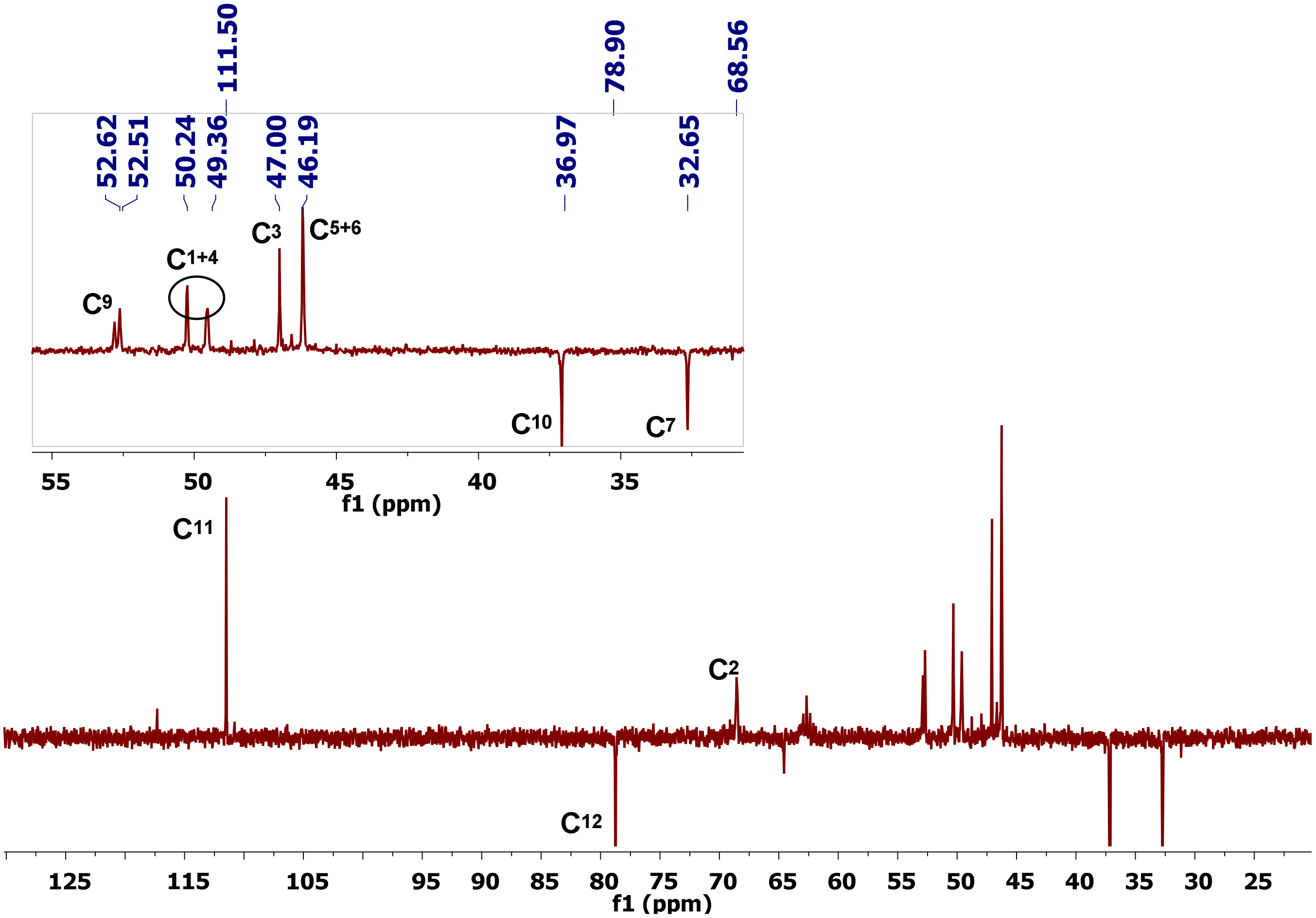

<u>Figure S4.</u> DEPT-135 of **2** in CD_3NO_2

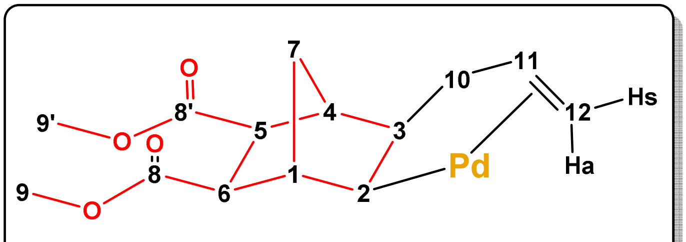

2

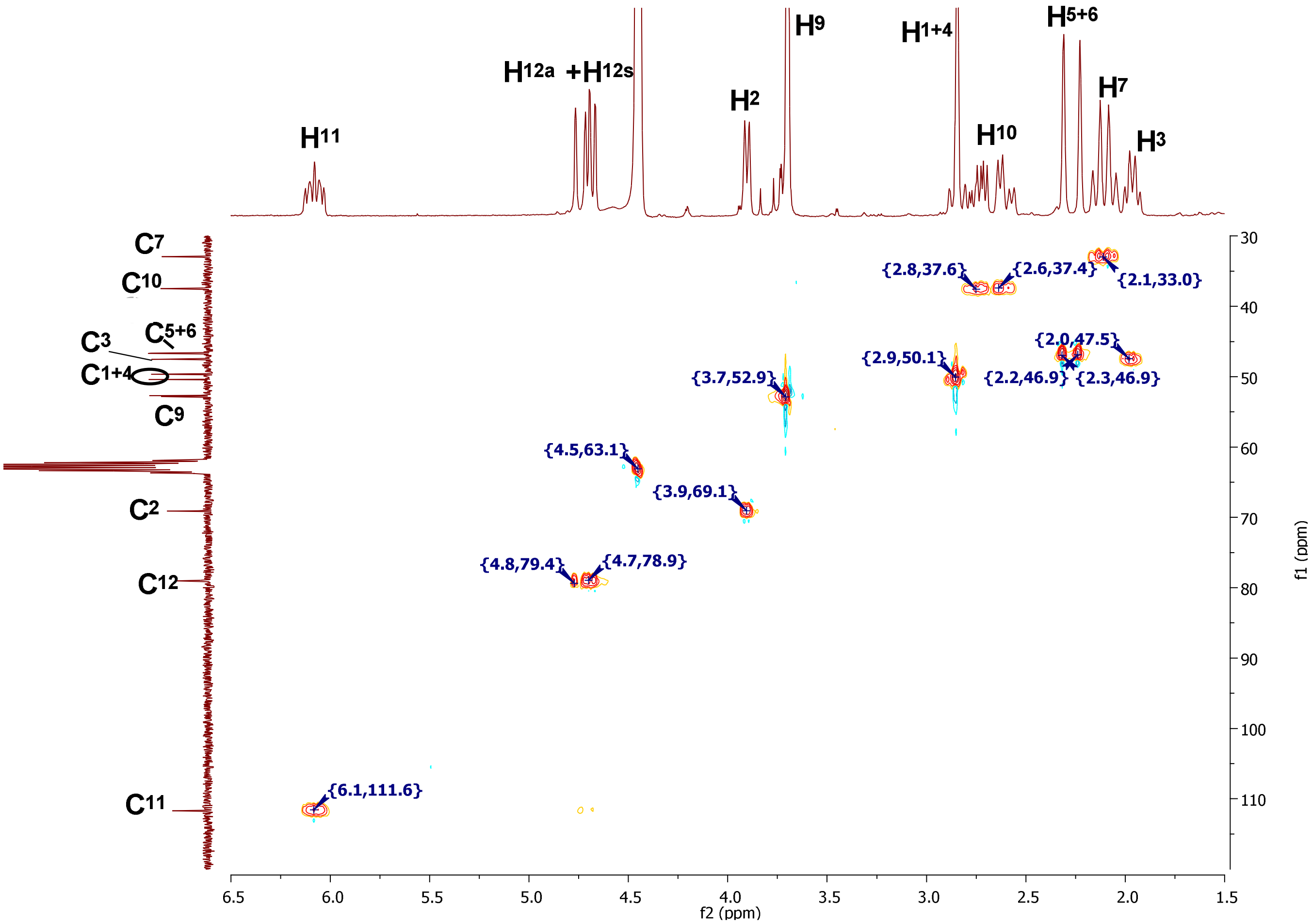

Figure S5. HSQC of **2** in CD_3NO_2

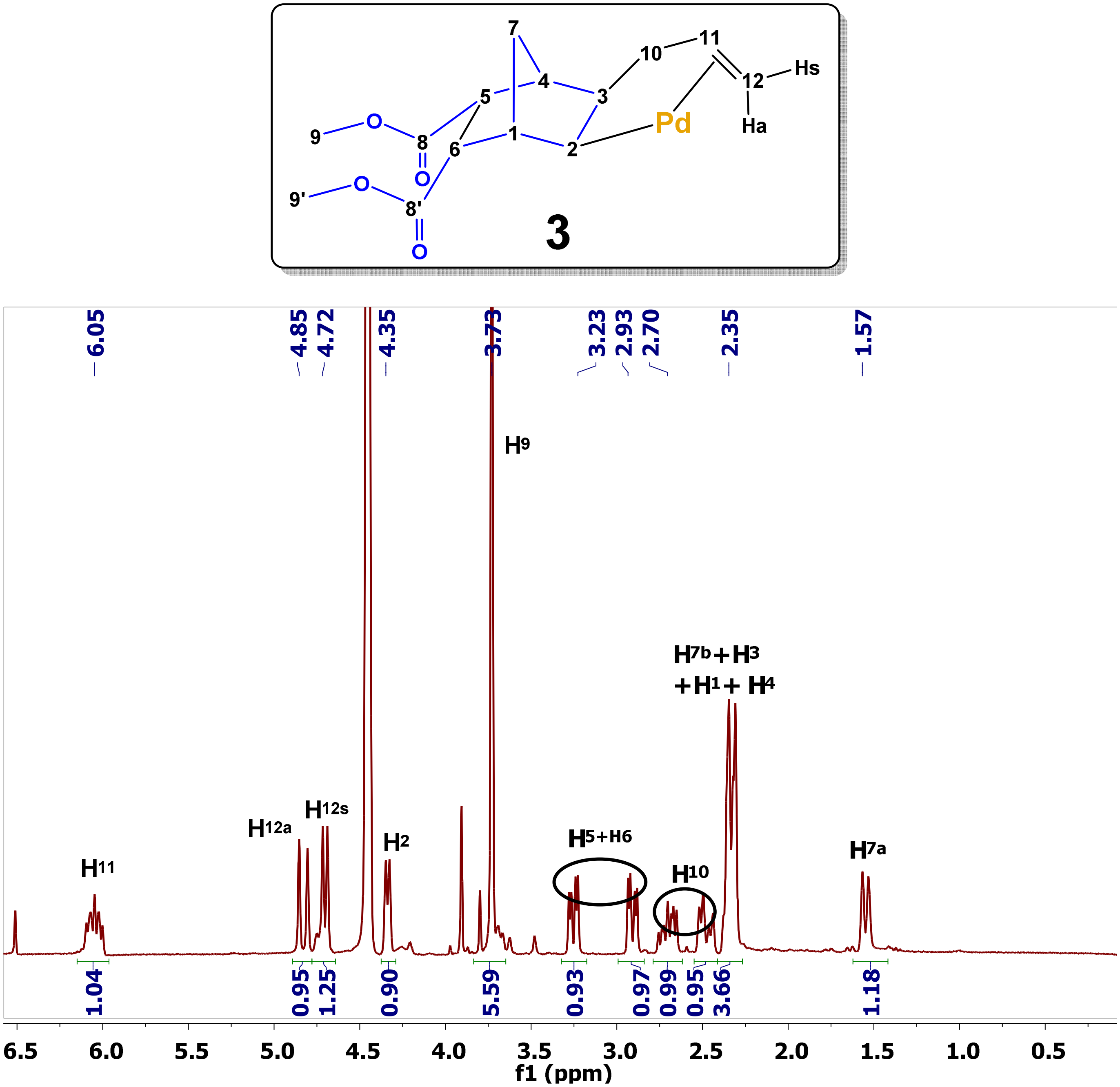

<u>Figure S6</u> ^{1}H NMR of **3** in CD_3NO_2

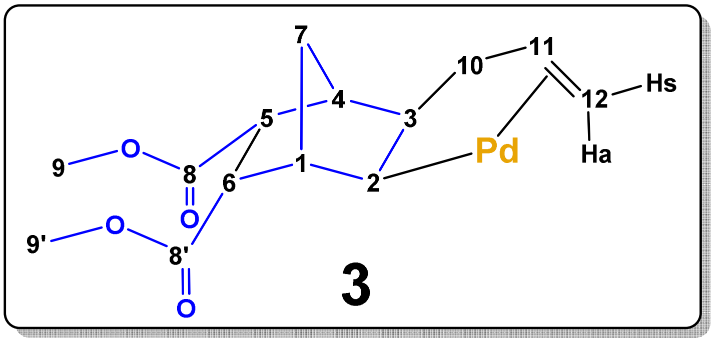

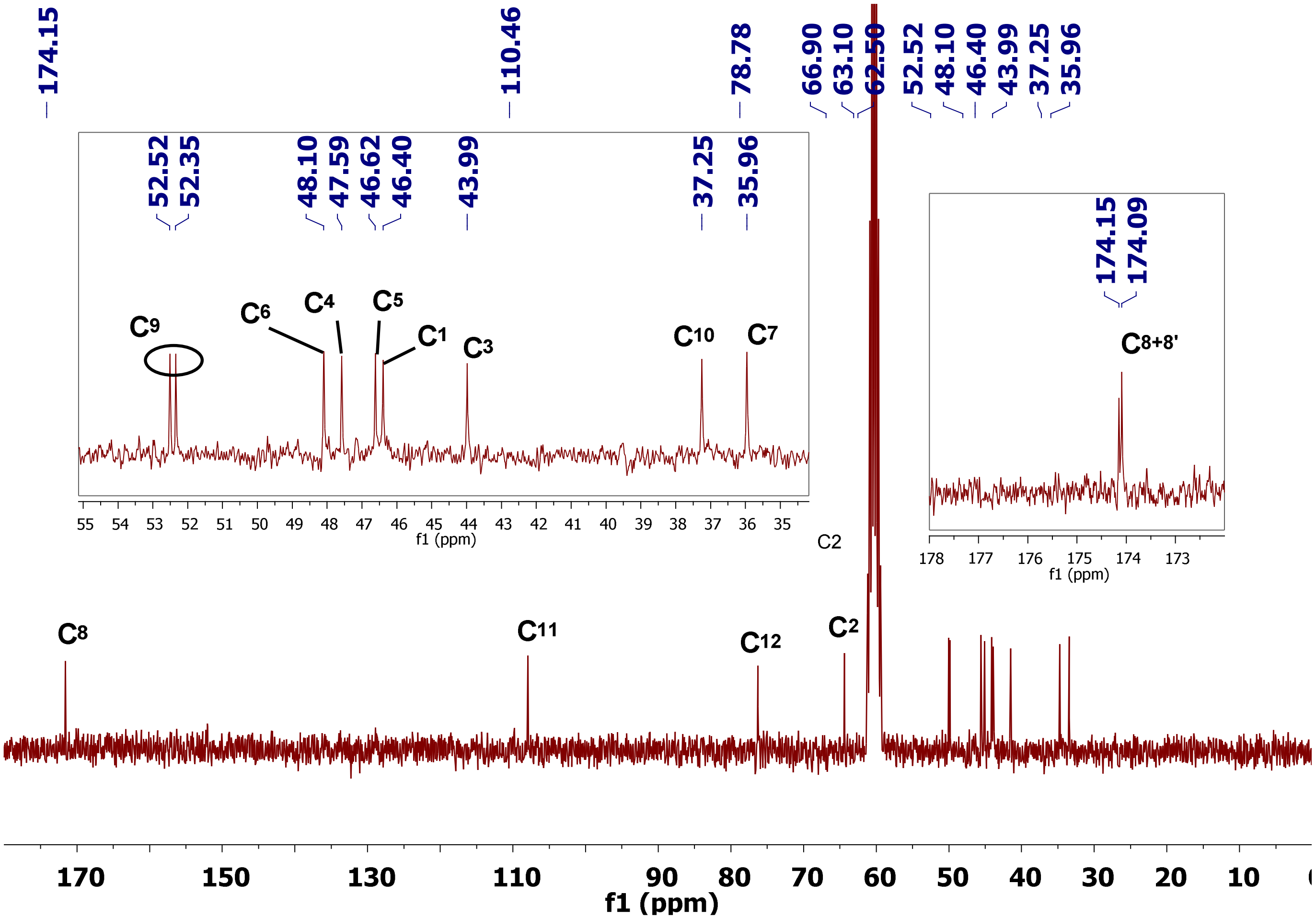

Figure S7. $^{13}C\{^{1}H\}$ NMR of **3** in CD_3NO_2

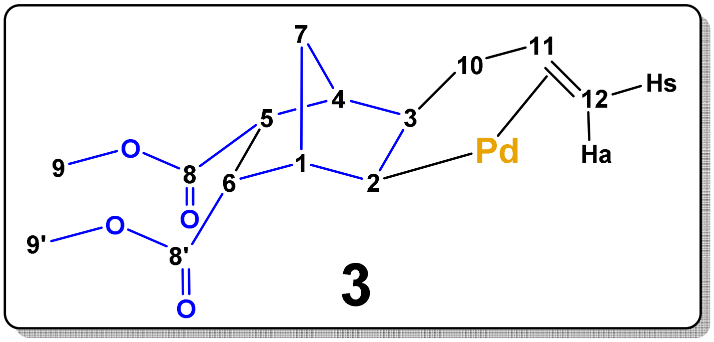

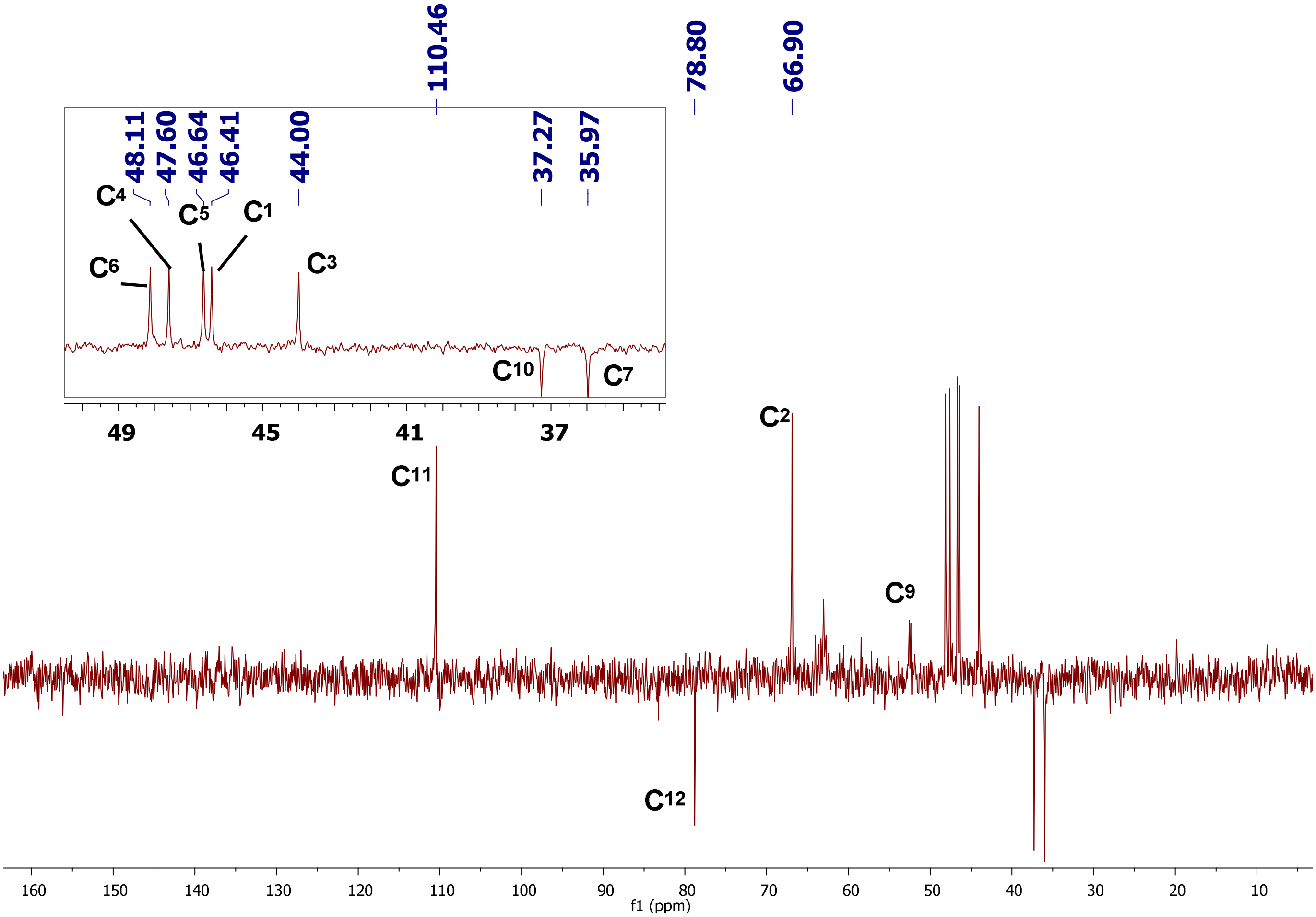

Figure S8. DEPT135 of **3** in CD_3NO_2

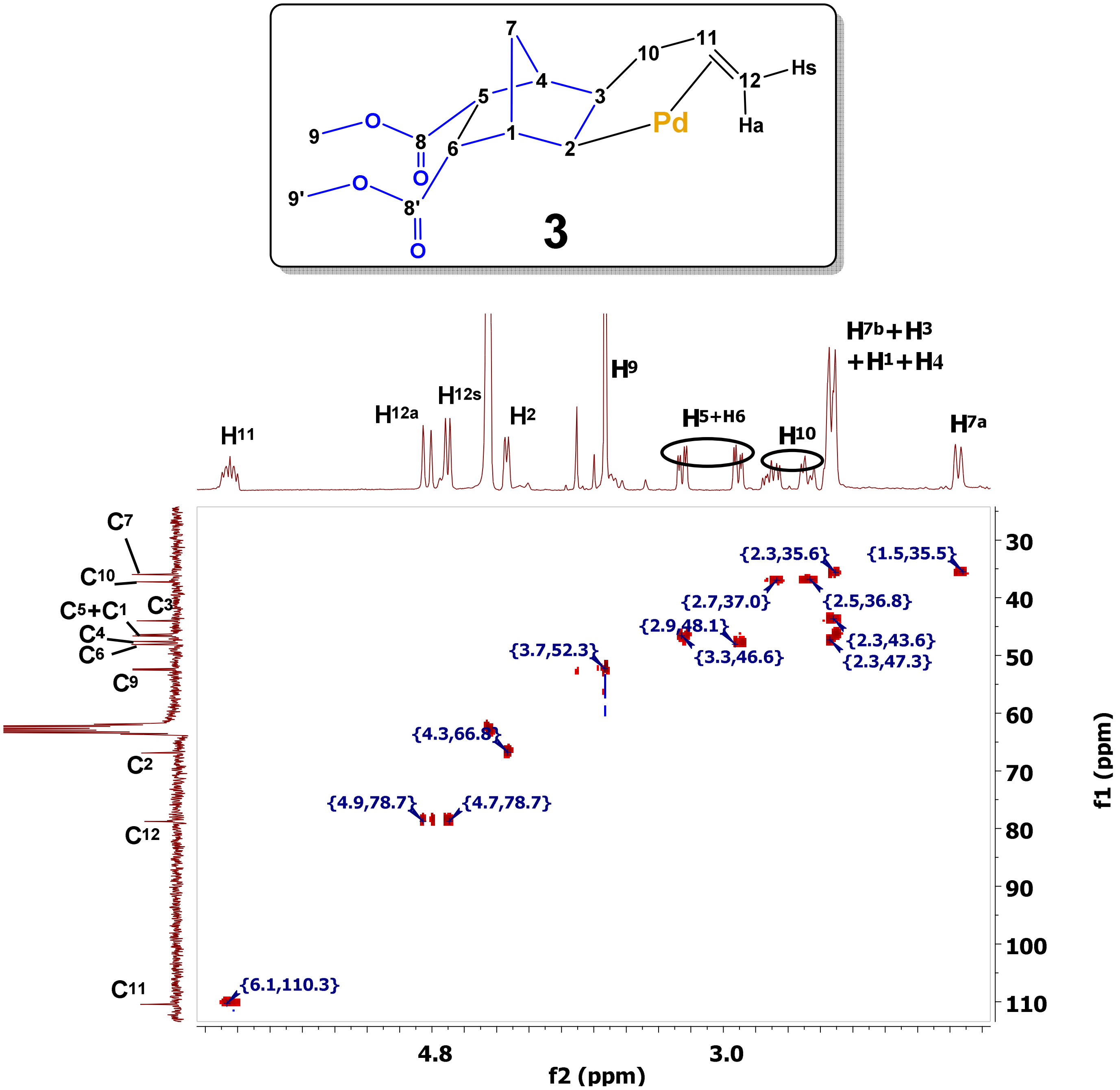

Figure S9. HSQC of **3** in CD_3NO_2

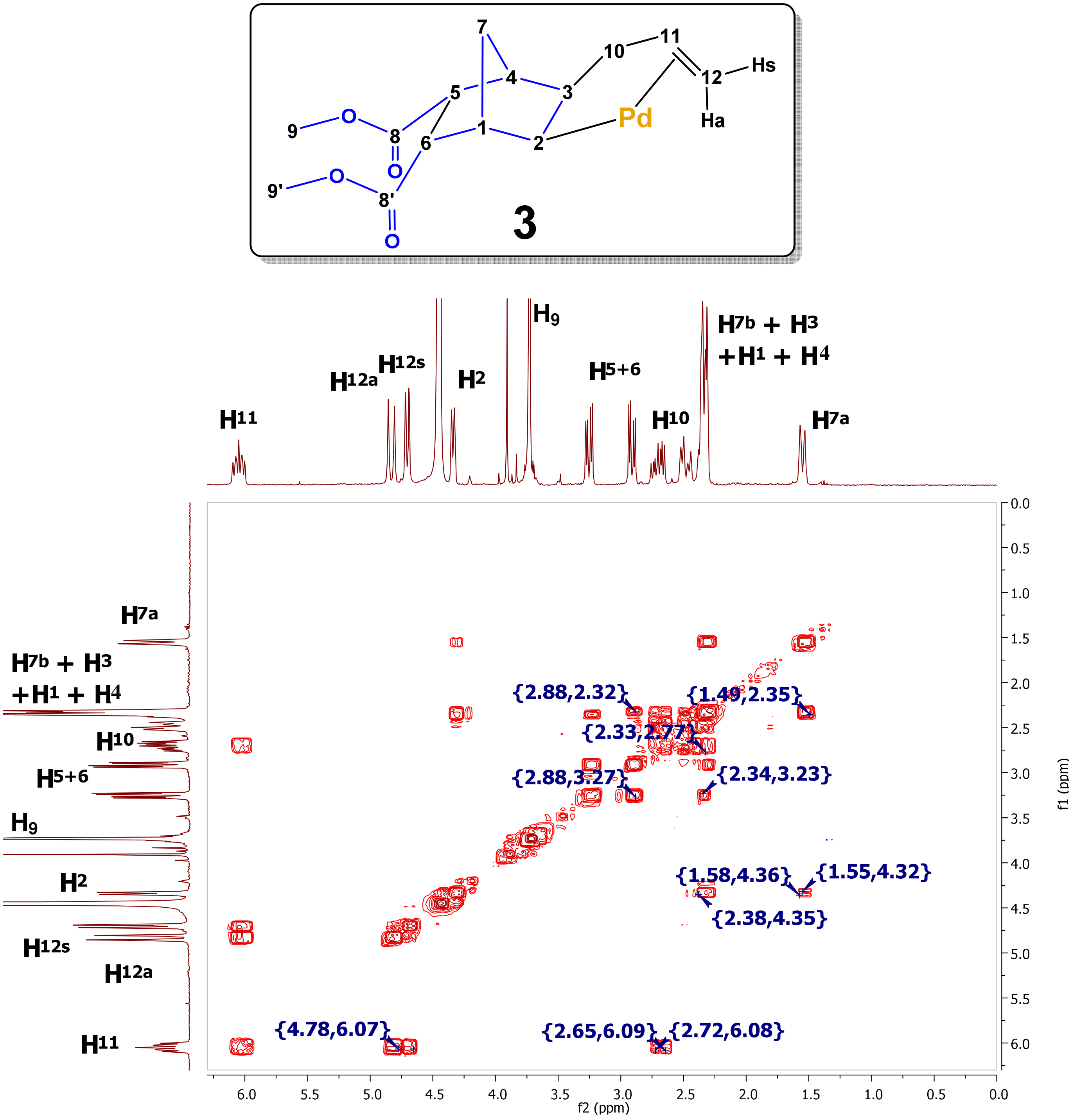

<u>Figure S10.</u> COSY of **3** in CD_3NO_2

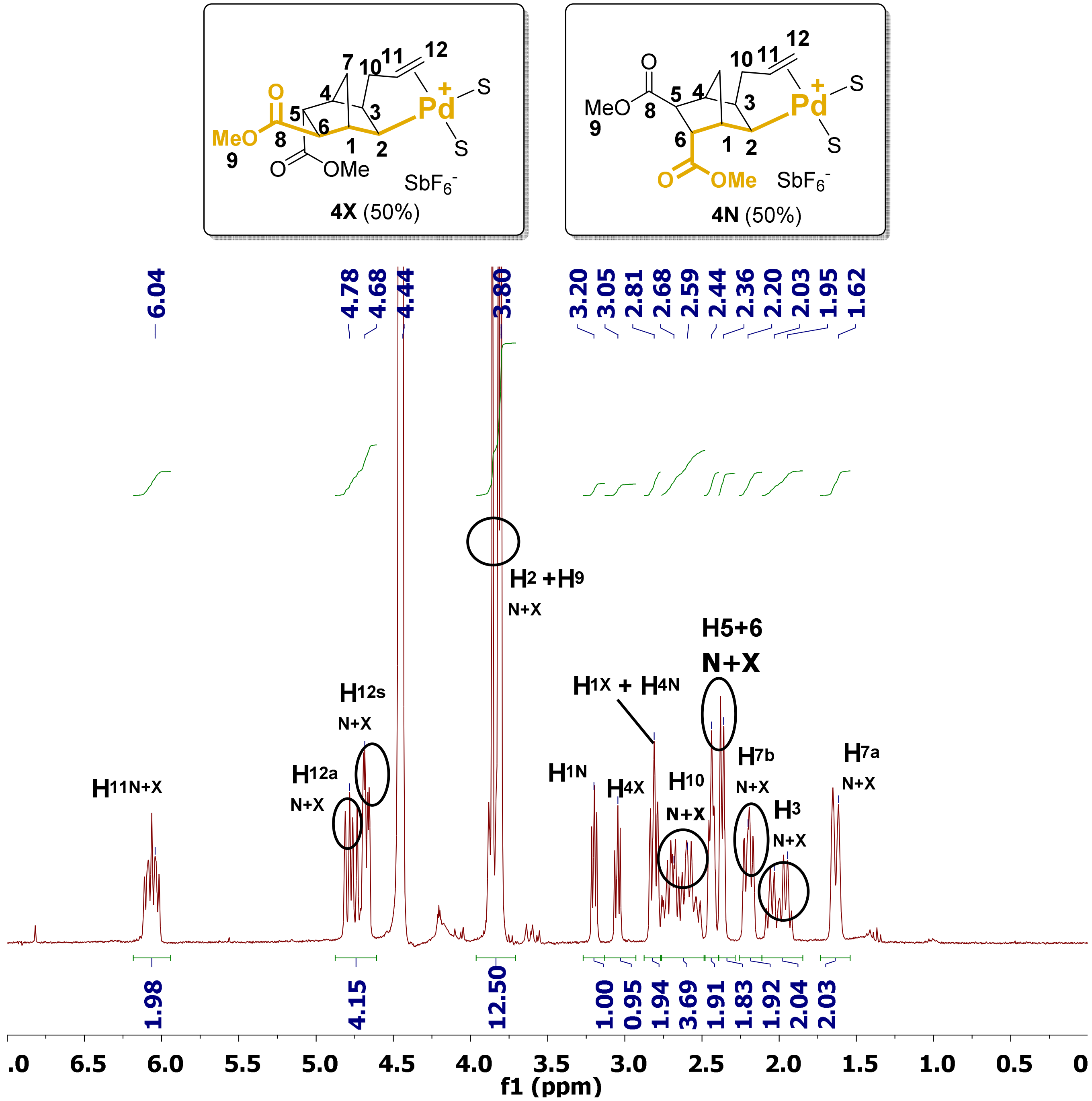

Figure S11. ^{1}H of **4** in CD_3NO_2

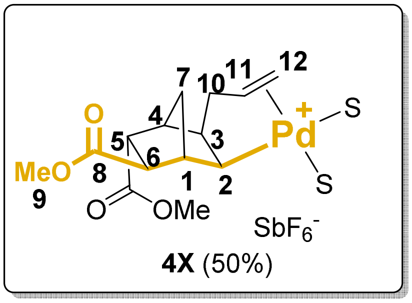

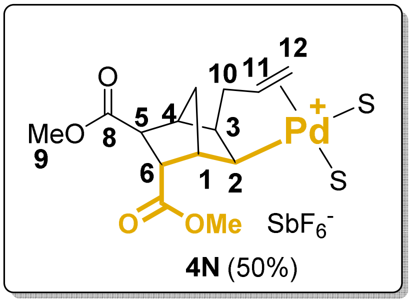

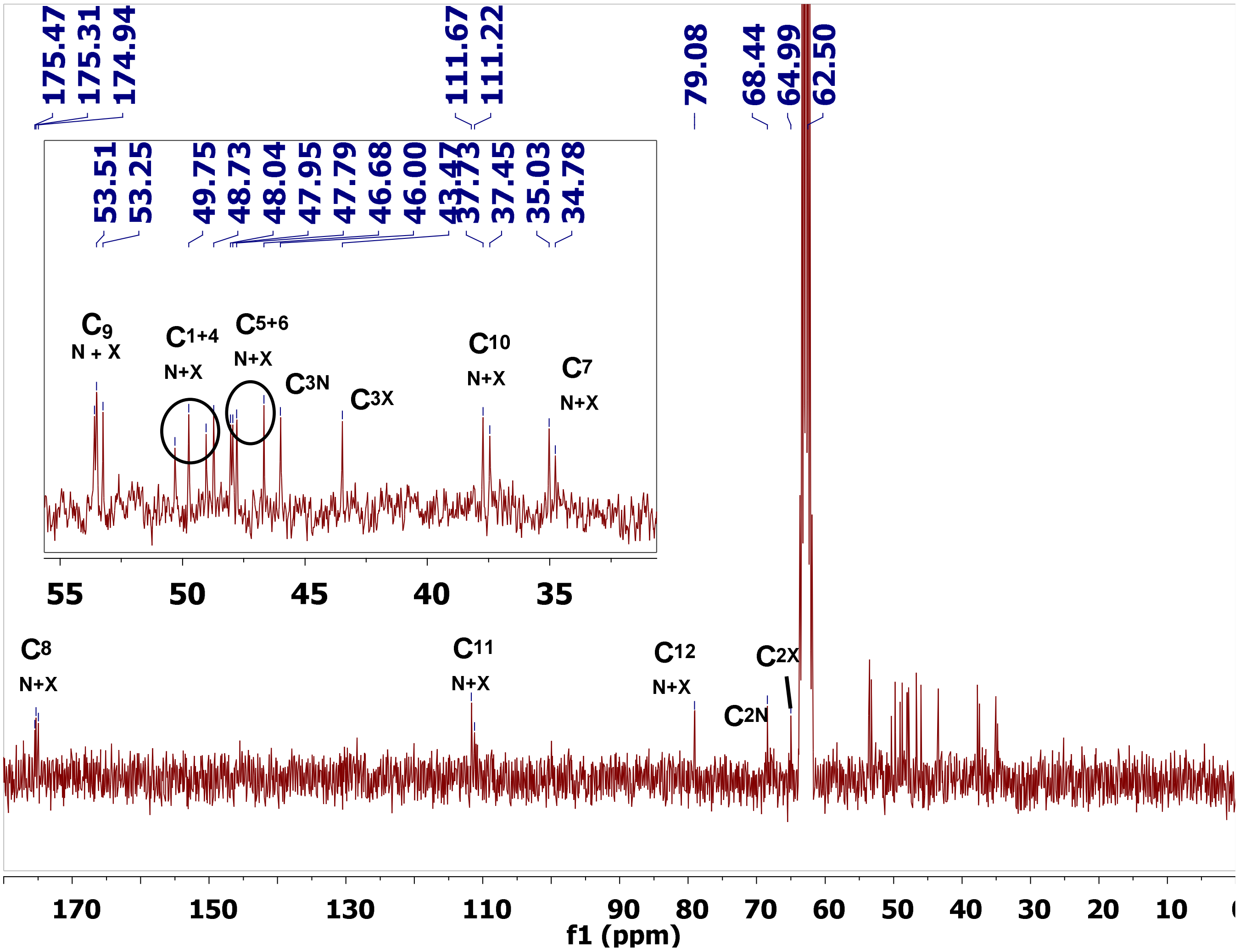

Figure S12. $^{13}C\{^1H\}$ of **4** in CD_3NO_2

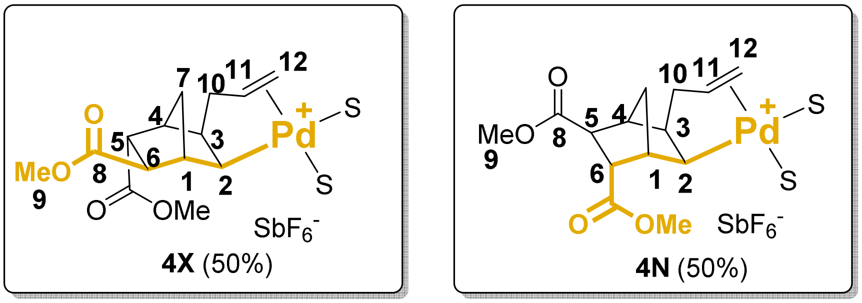

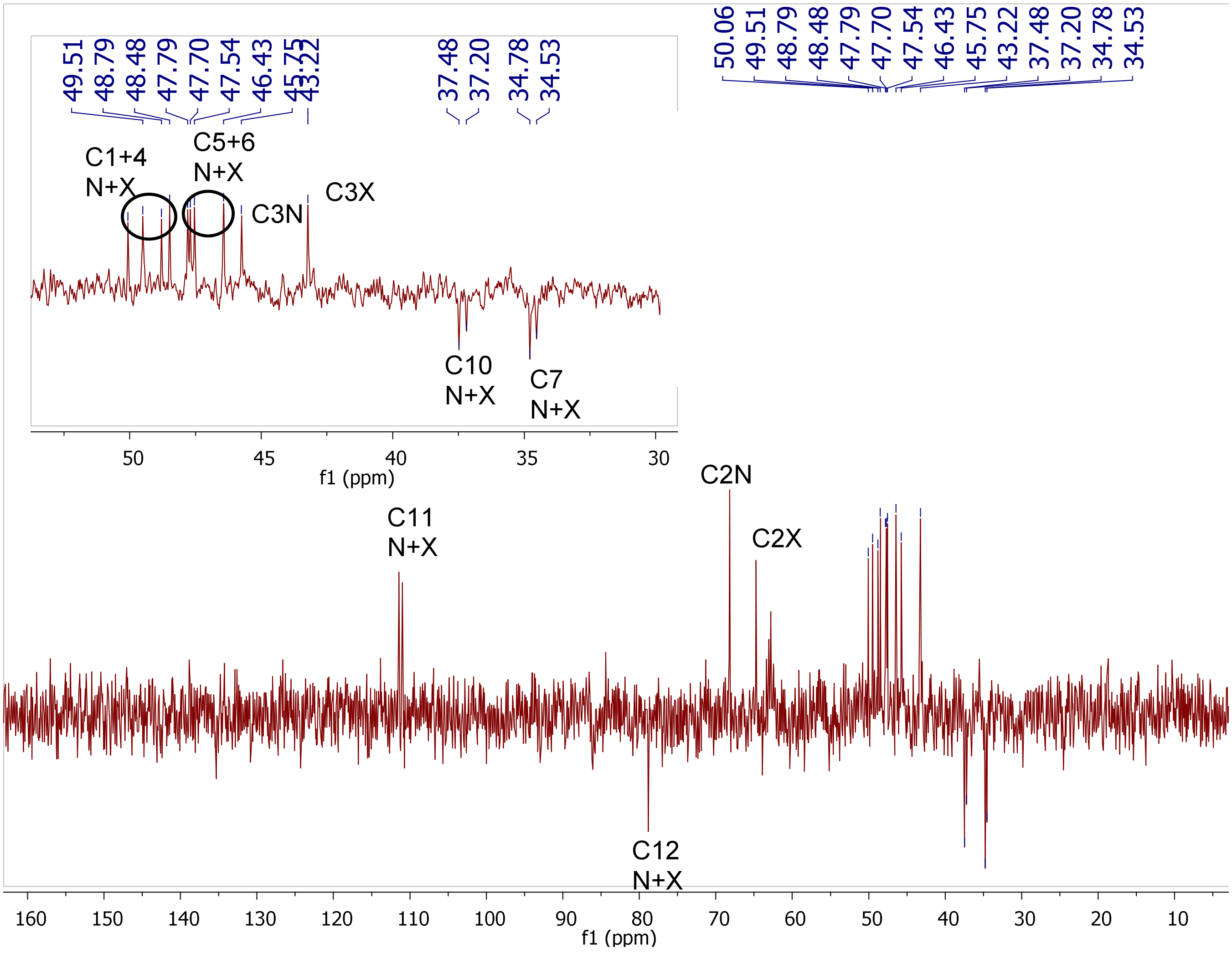

Figure S13. DEPT135 of **4** in CD_3NO_2

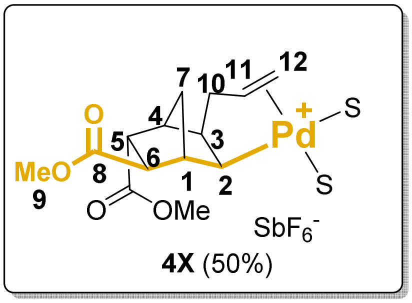

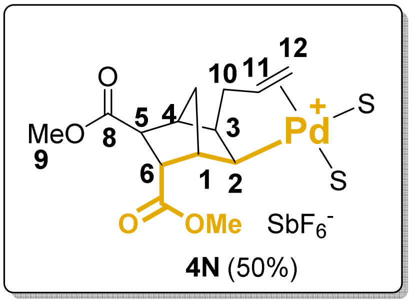

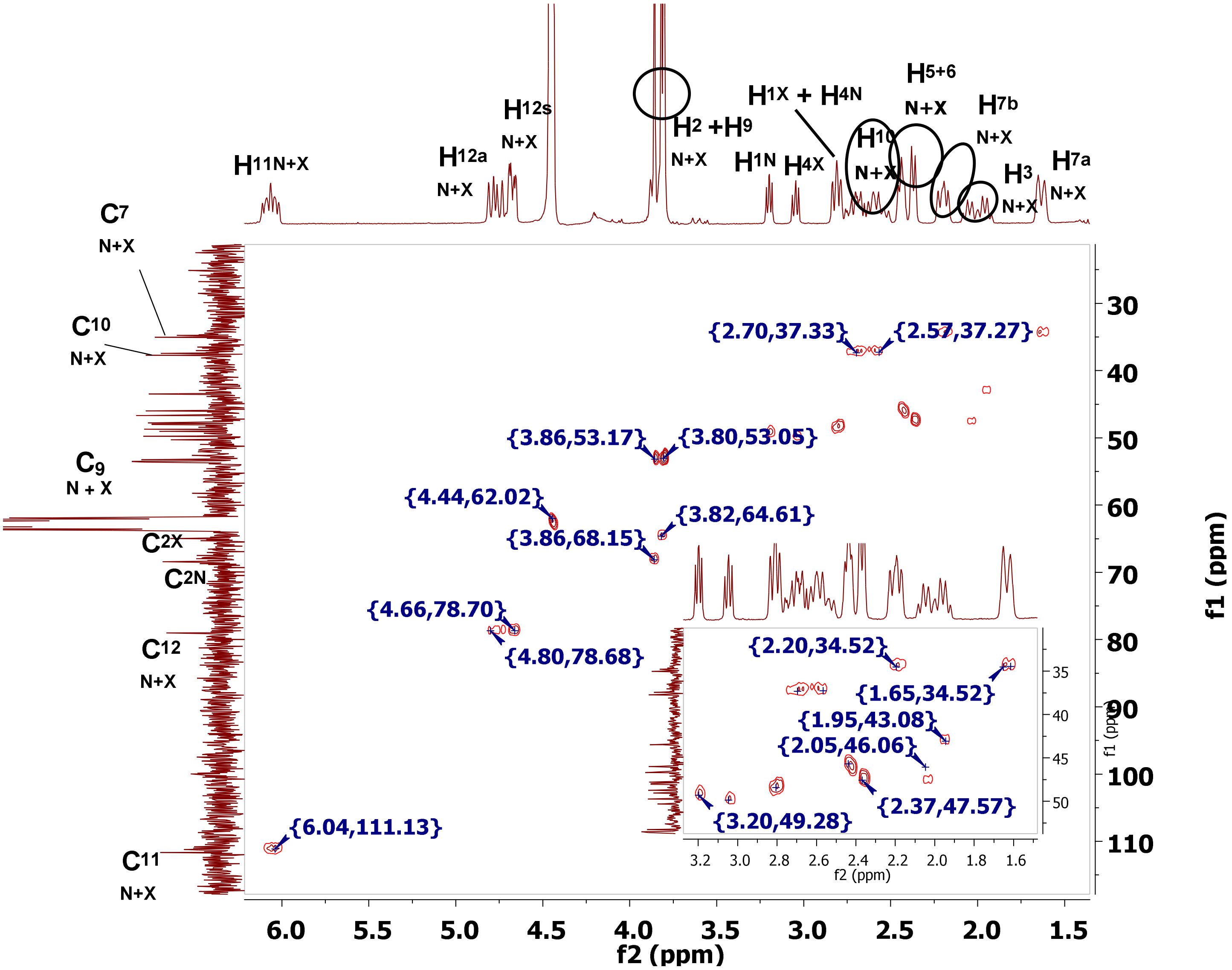

Figure S14. HSQC of **4** in CD_3NO_2

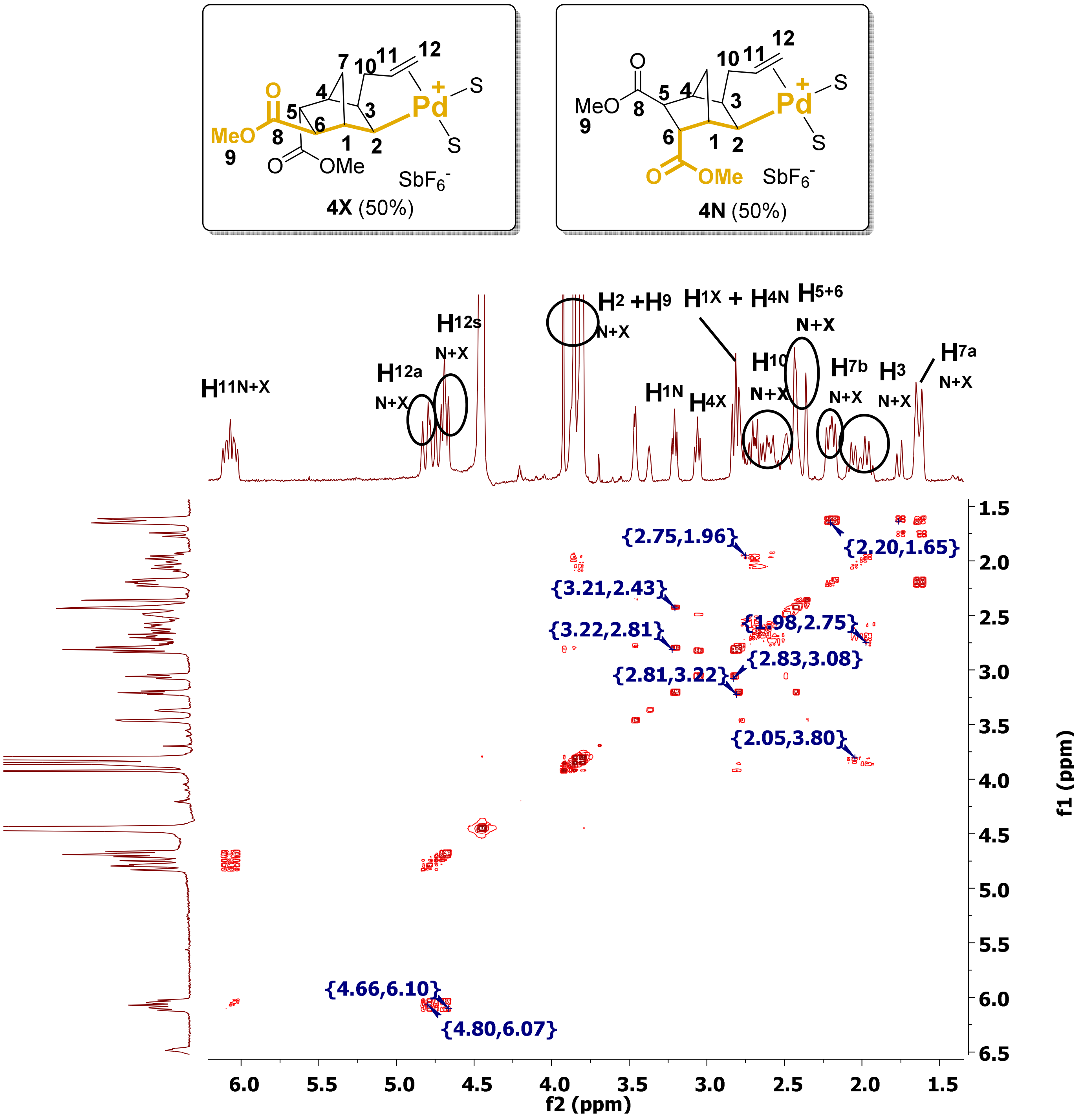

<u>Figure S15.</u> COSY of **4** in CD_3NO_2

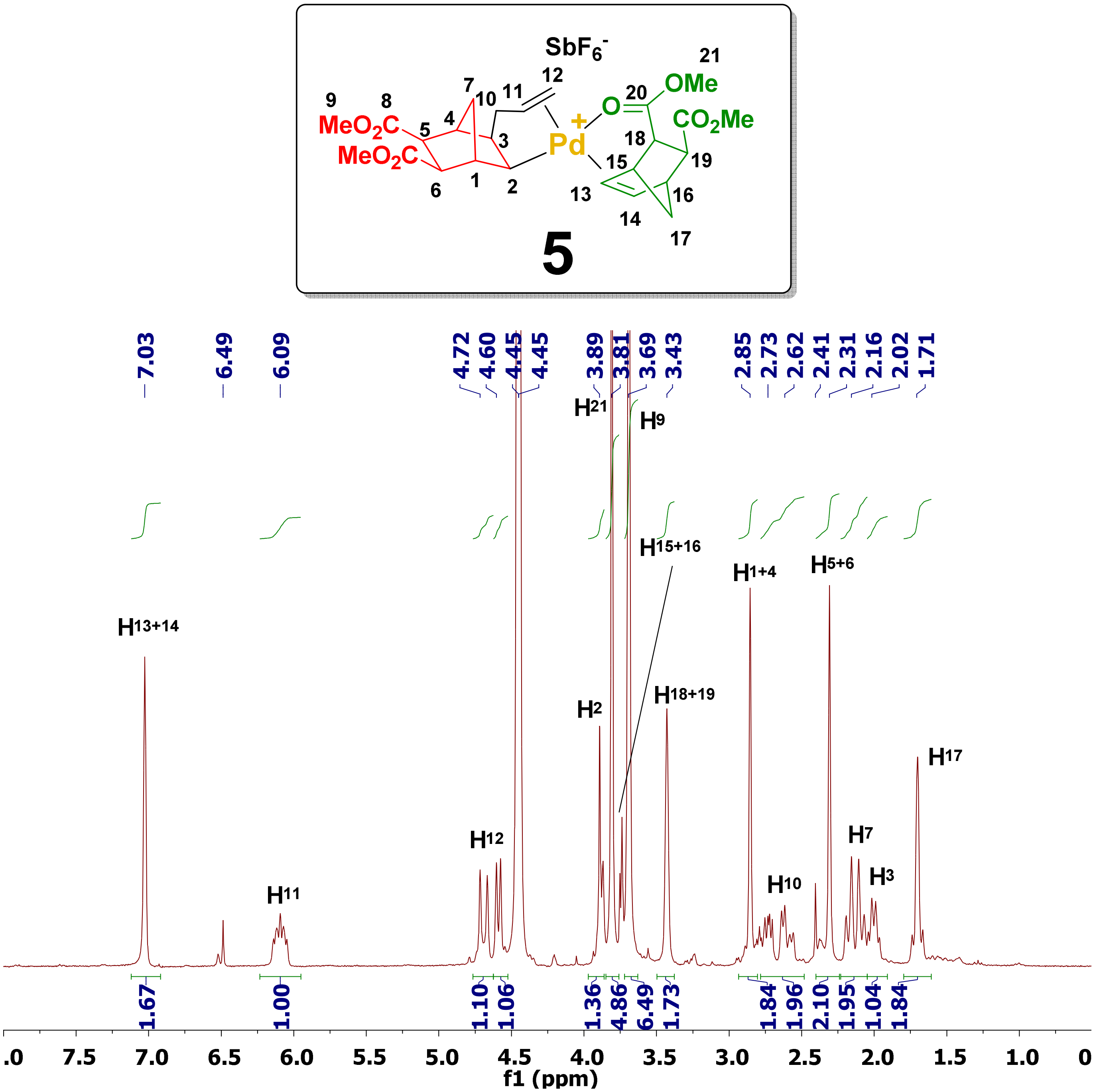

Figure S16. ^{1}H NMR of **5** in CD_3NO_2

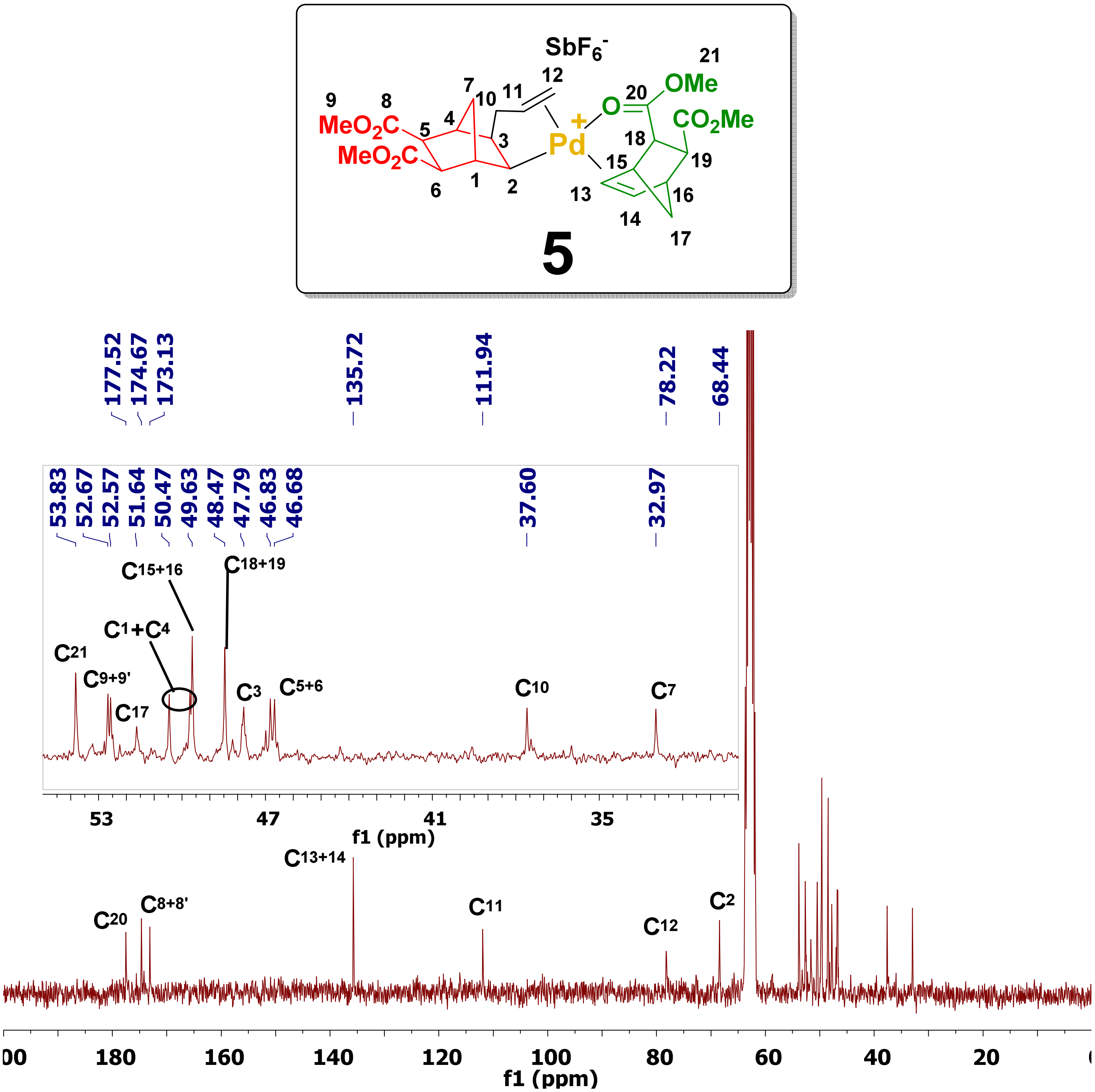

Figure S17. $^{13}C\{^1H\}$ of **5** in CD_3NO_2

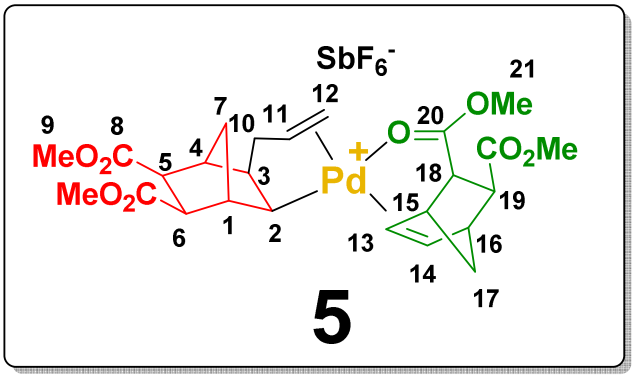

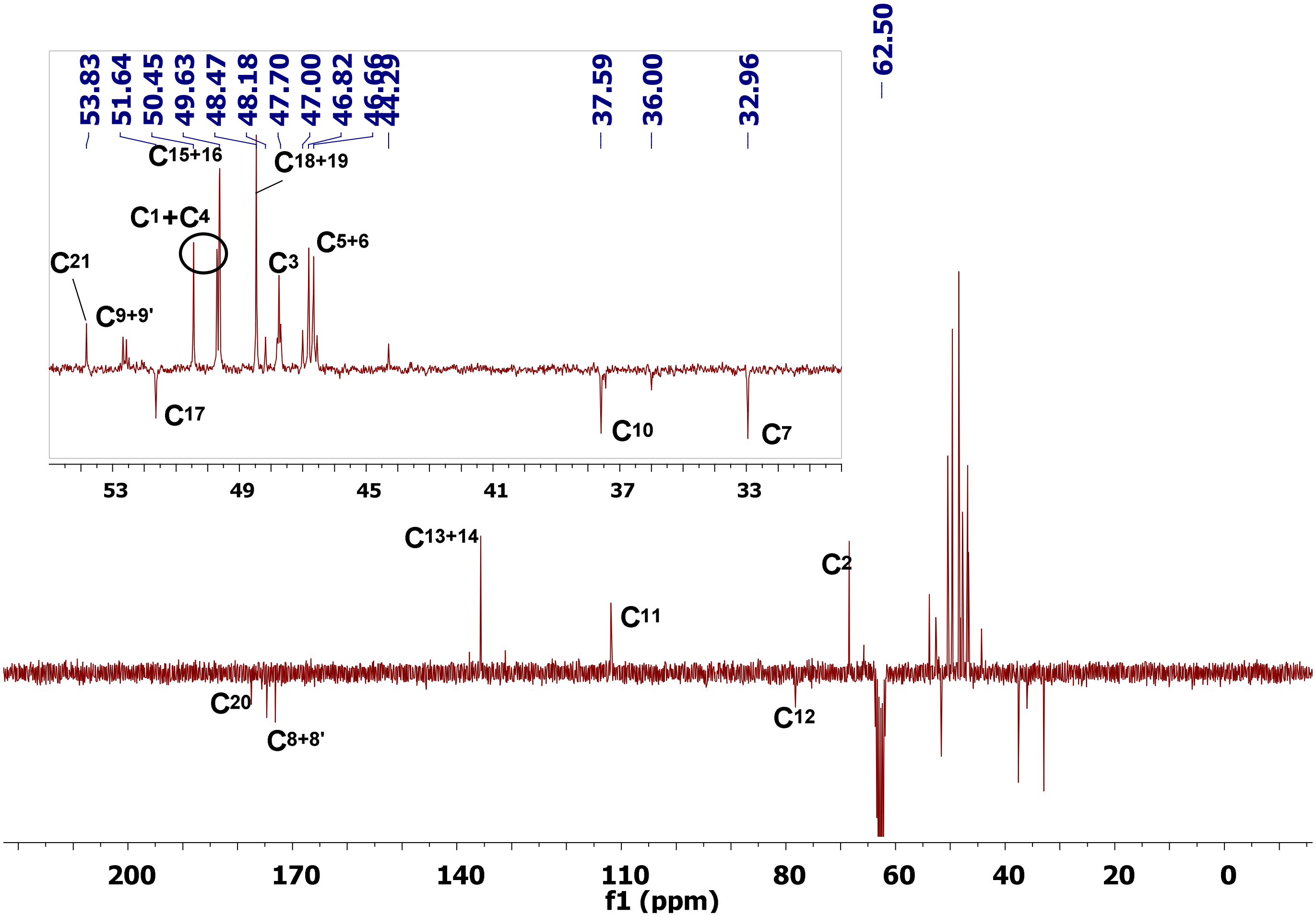

Figure S18. DEPT 135 of **5** in CD_3NO_2

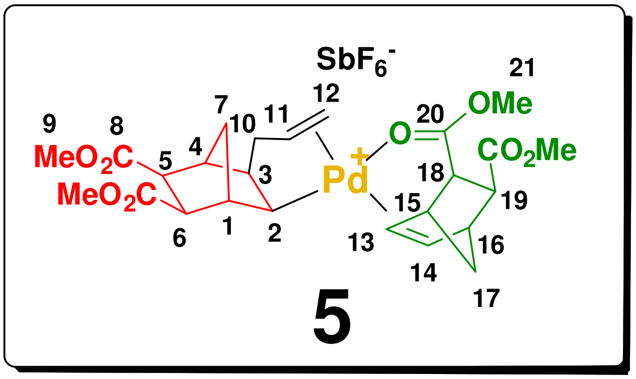

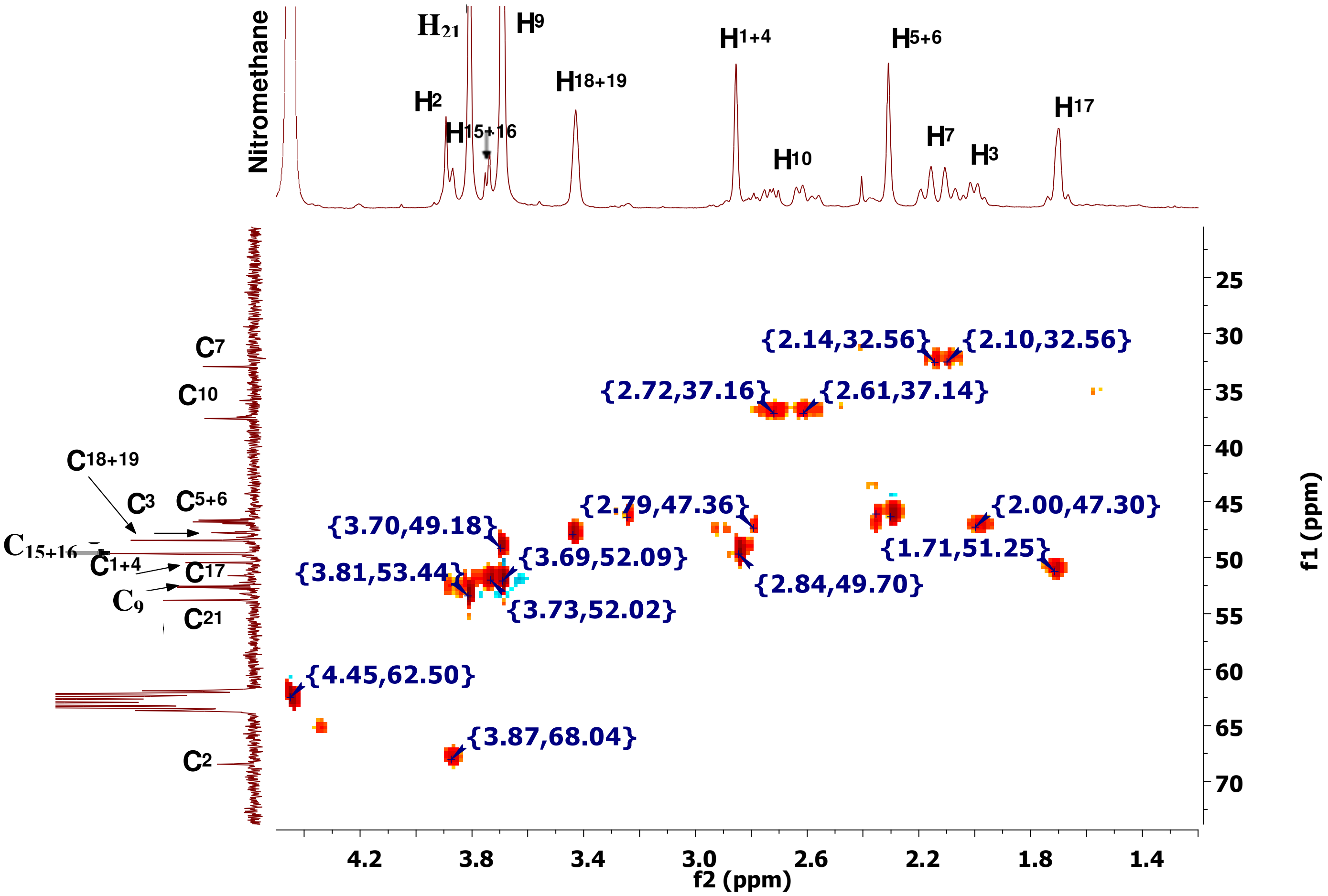

Figure S19. HMBC of **5** in CD_3NO_2

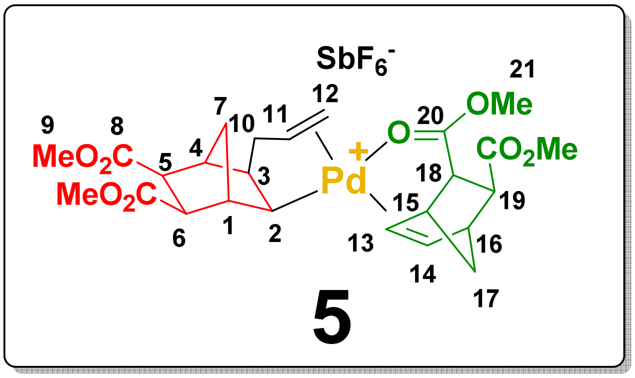

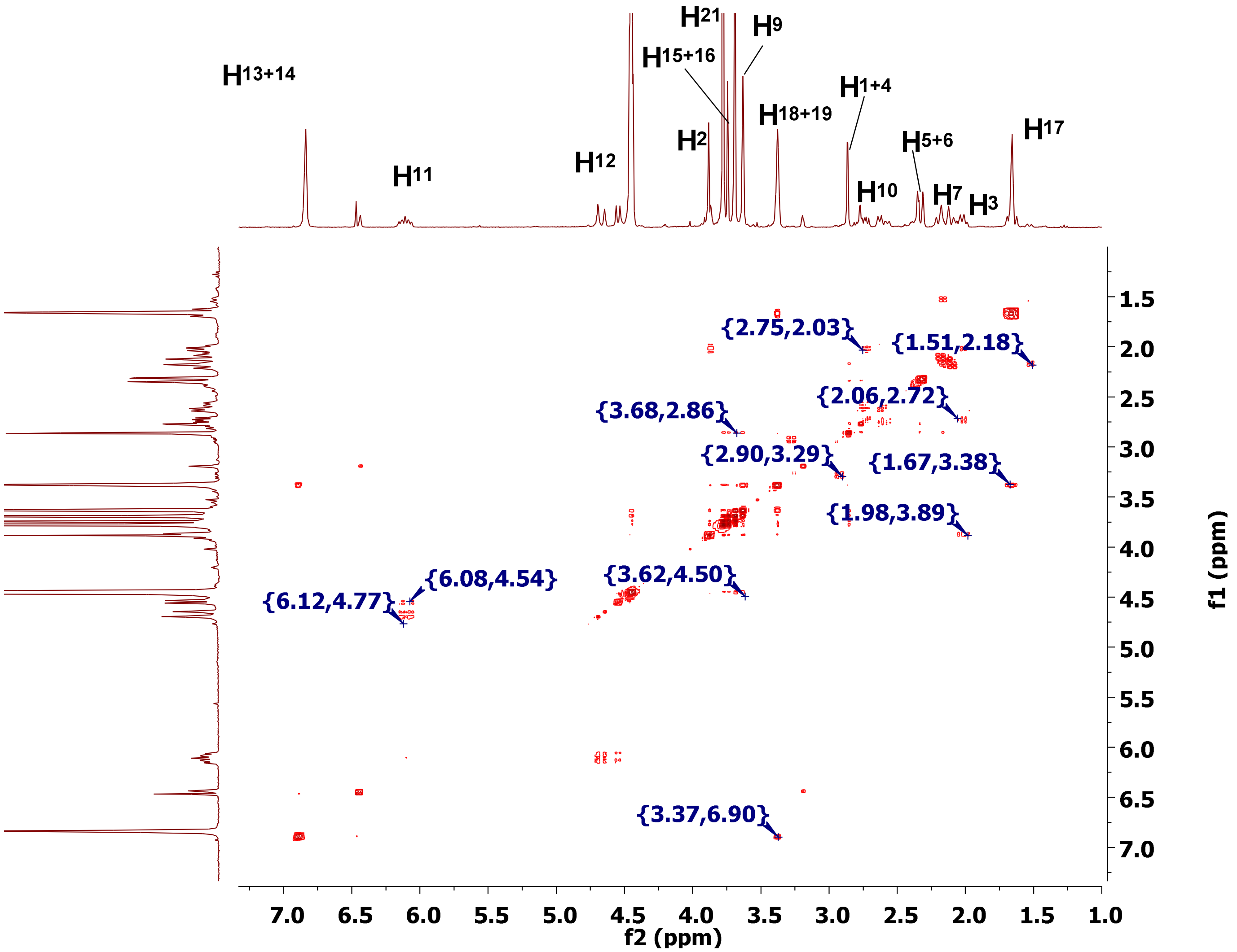

<u>Figure S20.</u> COSY of **5** in CD_3NO_2

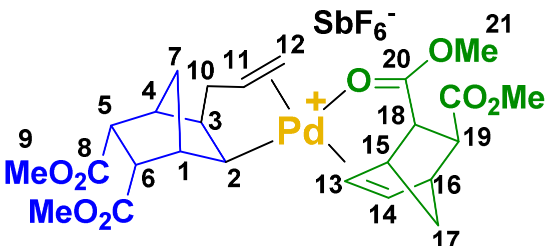

6

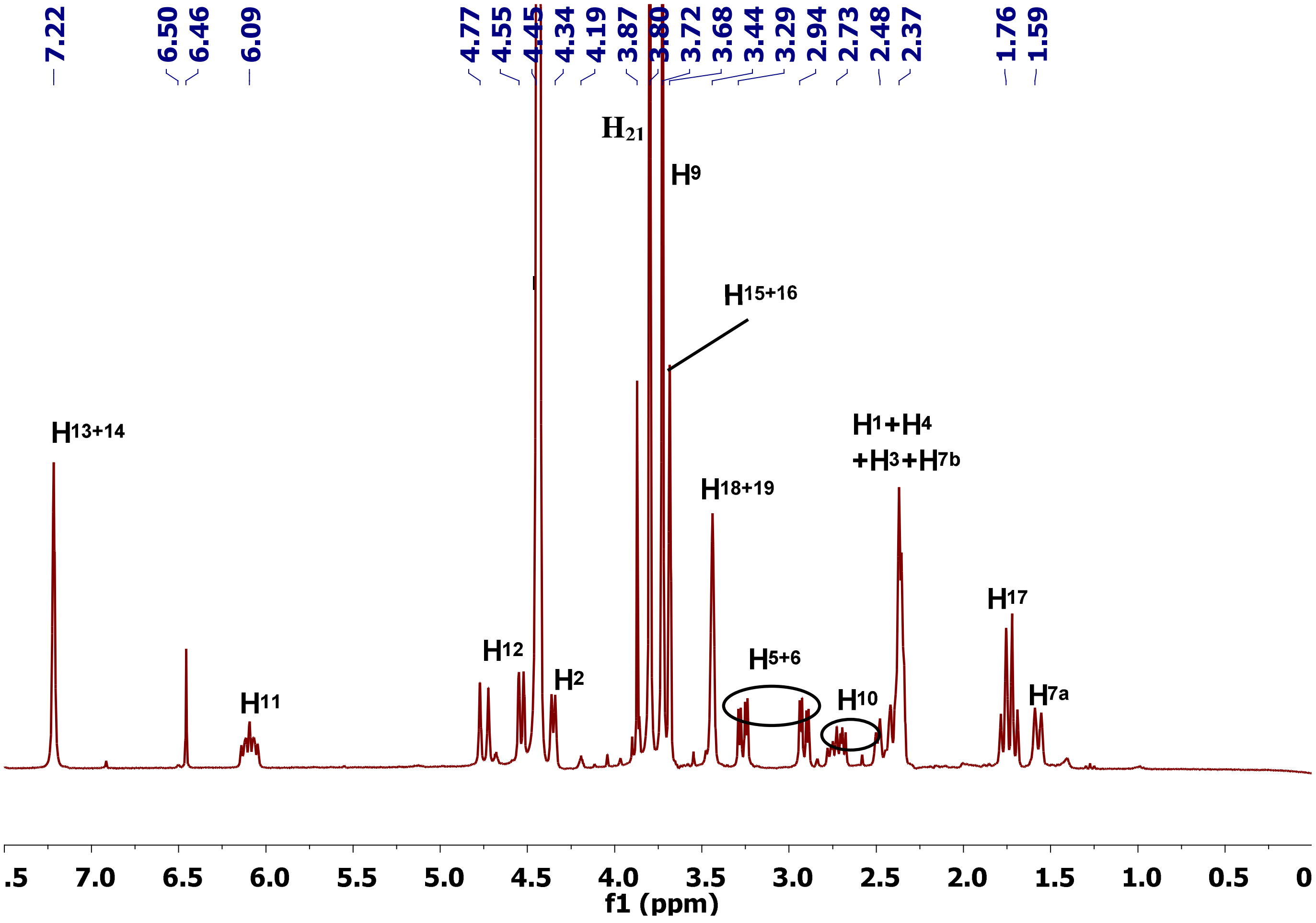

Figure S21. [1]H of **6** in CD_3NO_2 (impurity: dimethyl fumarate, less than 5%).

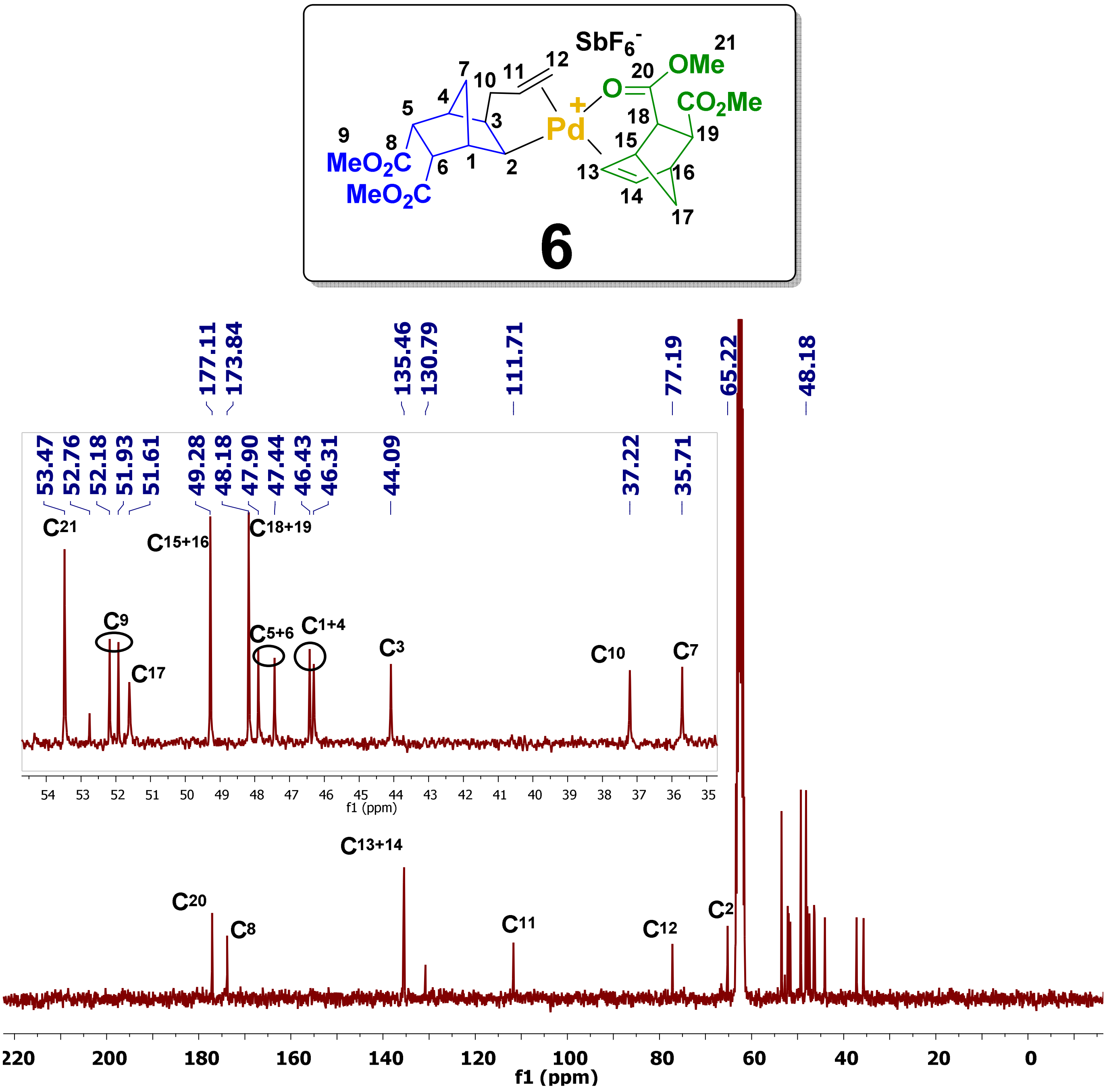

Figure S22. $^{13}C\{^{1}H\}$ NMR of **6** in CD_3NO_2 (impurity: dimethyl fumarate, less than 5%).

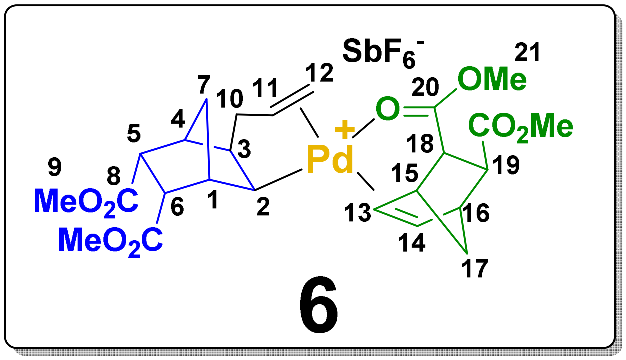

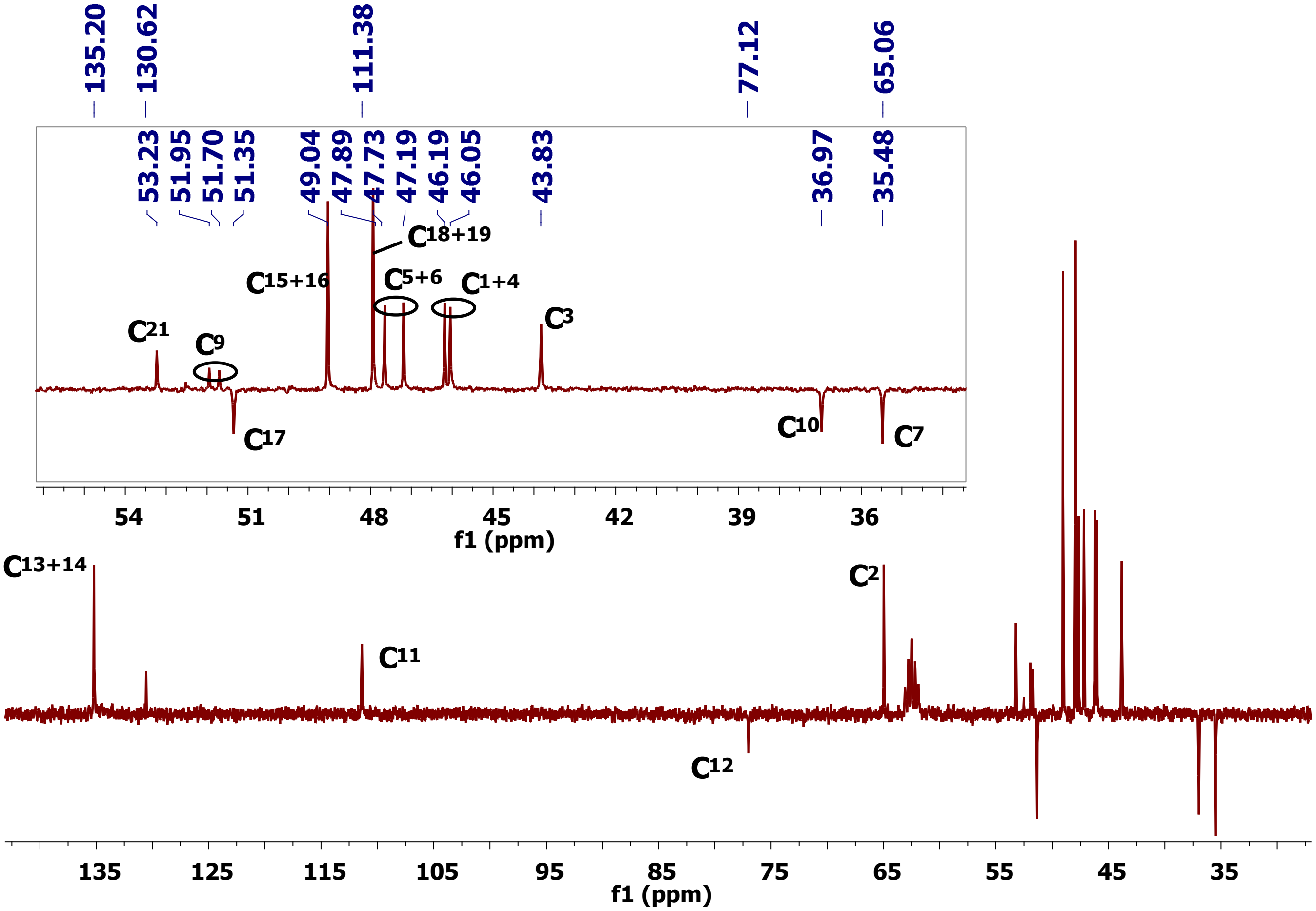

Figure S23. DEPT135 of **6** in CD_3NO_2

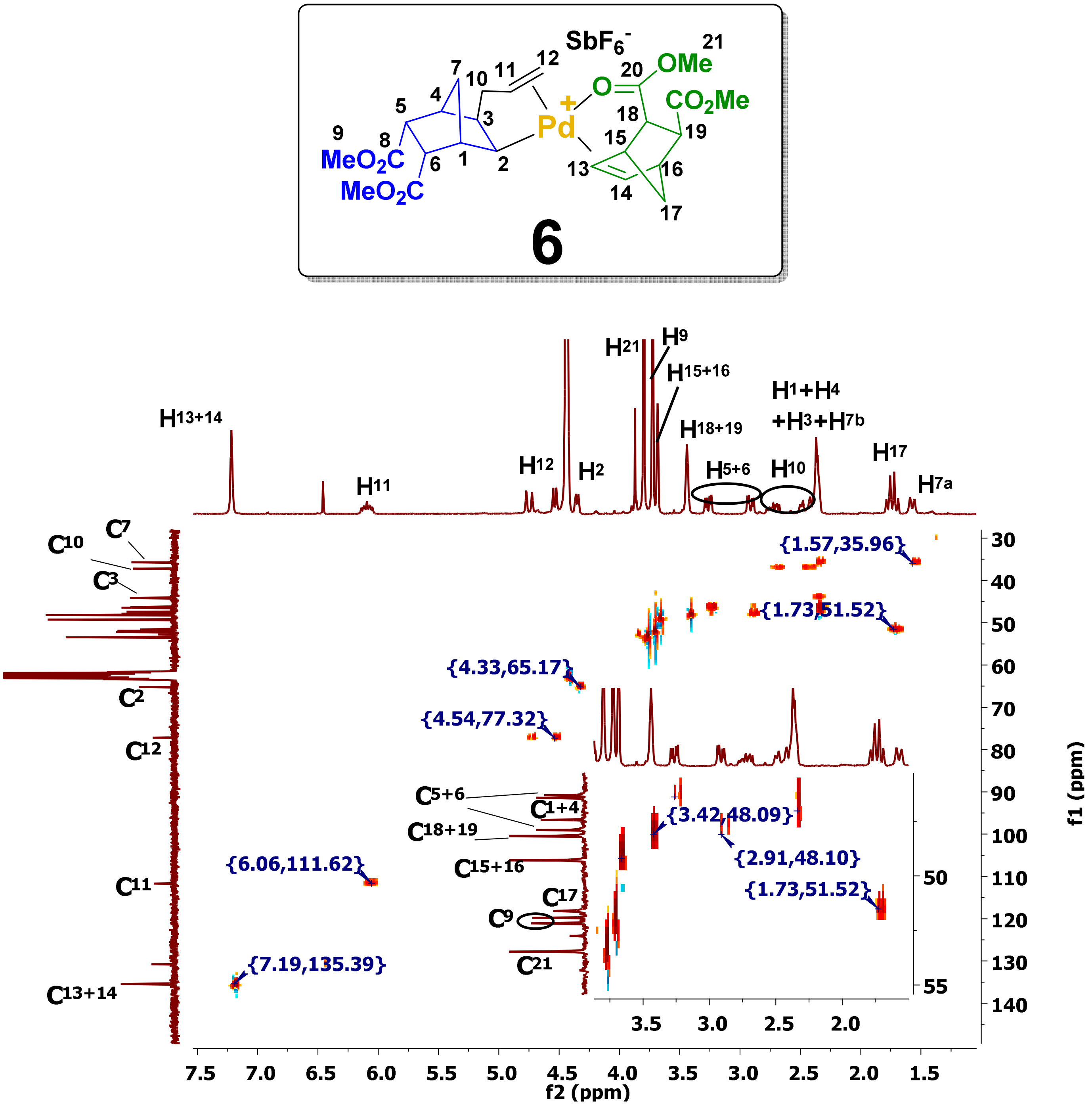

<u>Figure S24.</u> HSQC of **6** in CD_3NO_2

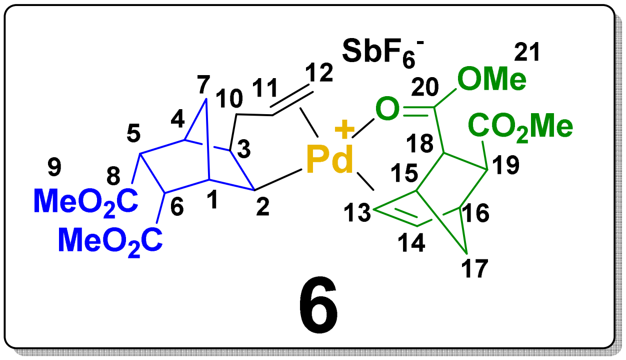

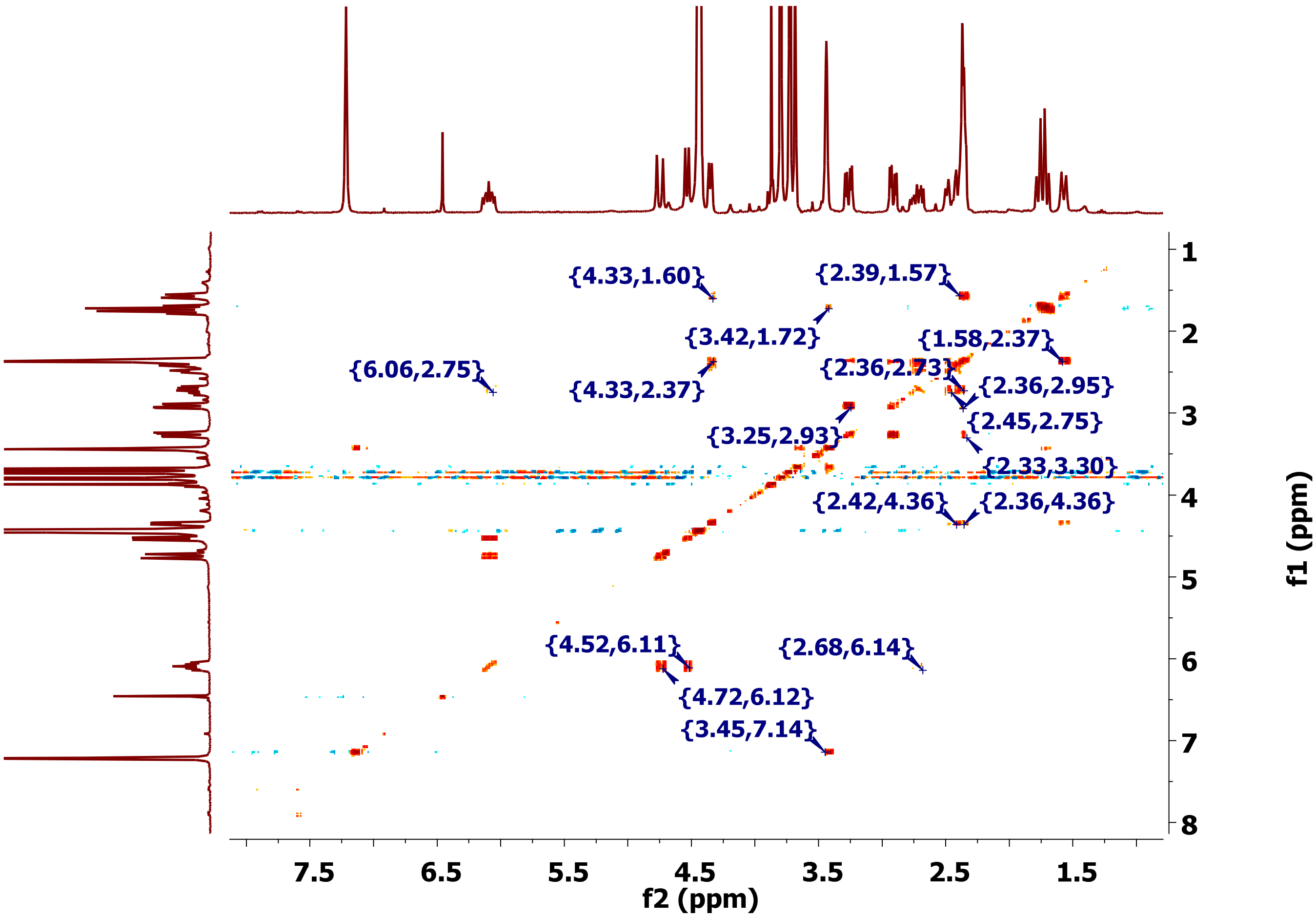

<u>Figure S25.</u> COSY of **6** in CD_3NO_2

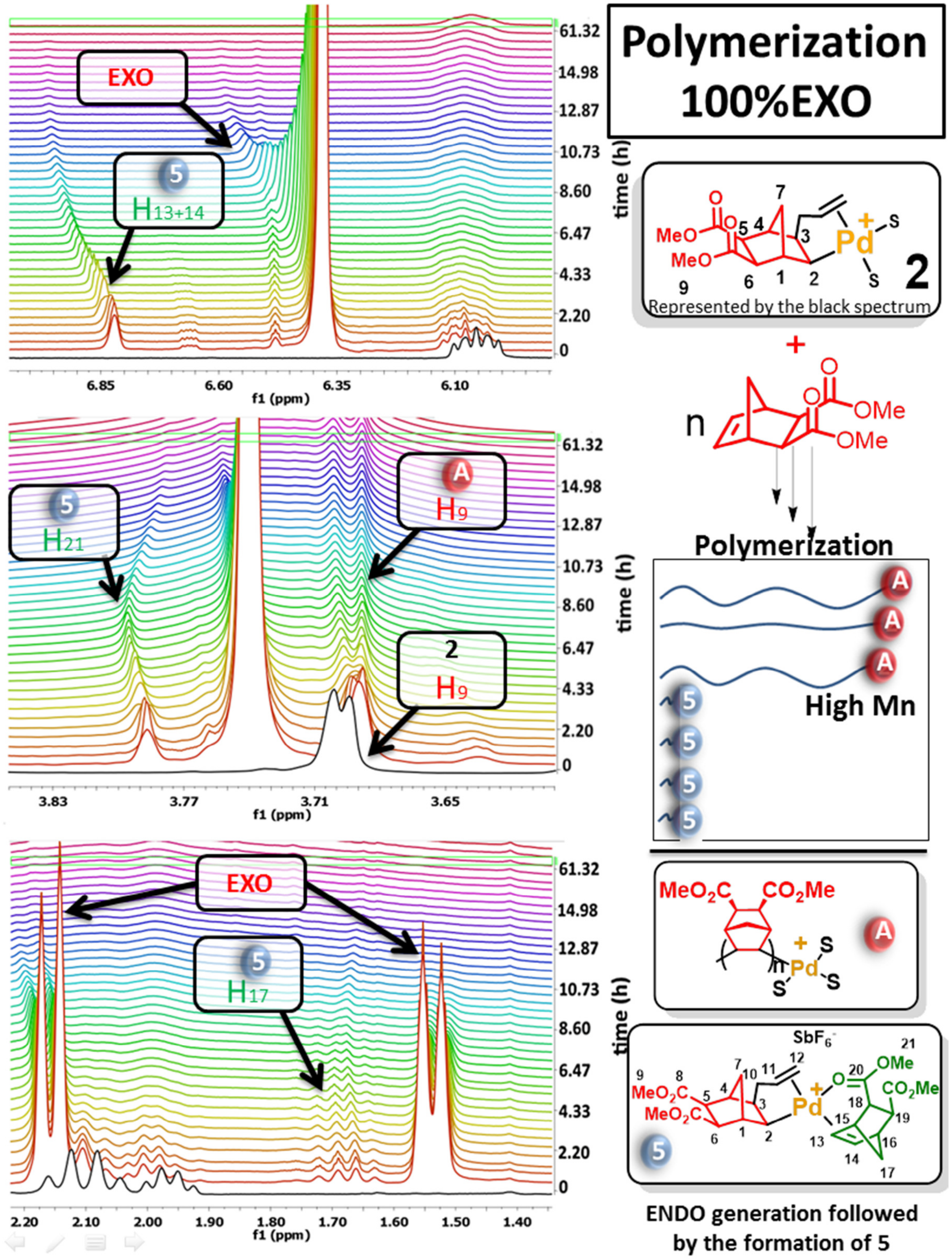

Figure S26. ^{1}H NMR spectra of the polymerization of $NBE(CO_2Me)_2$ (100% *exo*) with **2** in CD_3NO_2. Top: decrease of the *exo* monomer olefinic resonance at 6.35 ppm, and apparition of the chelated catalyst **5** (not initially present in **2**). Center: OCH_3 resonances, indicating the presence of a chelate (protons H_{21}). Bottom, characteristic bridge protons H_{17} of the chelated catalyst **5**.

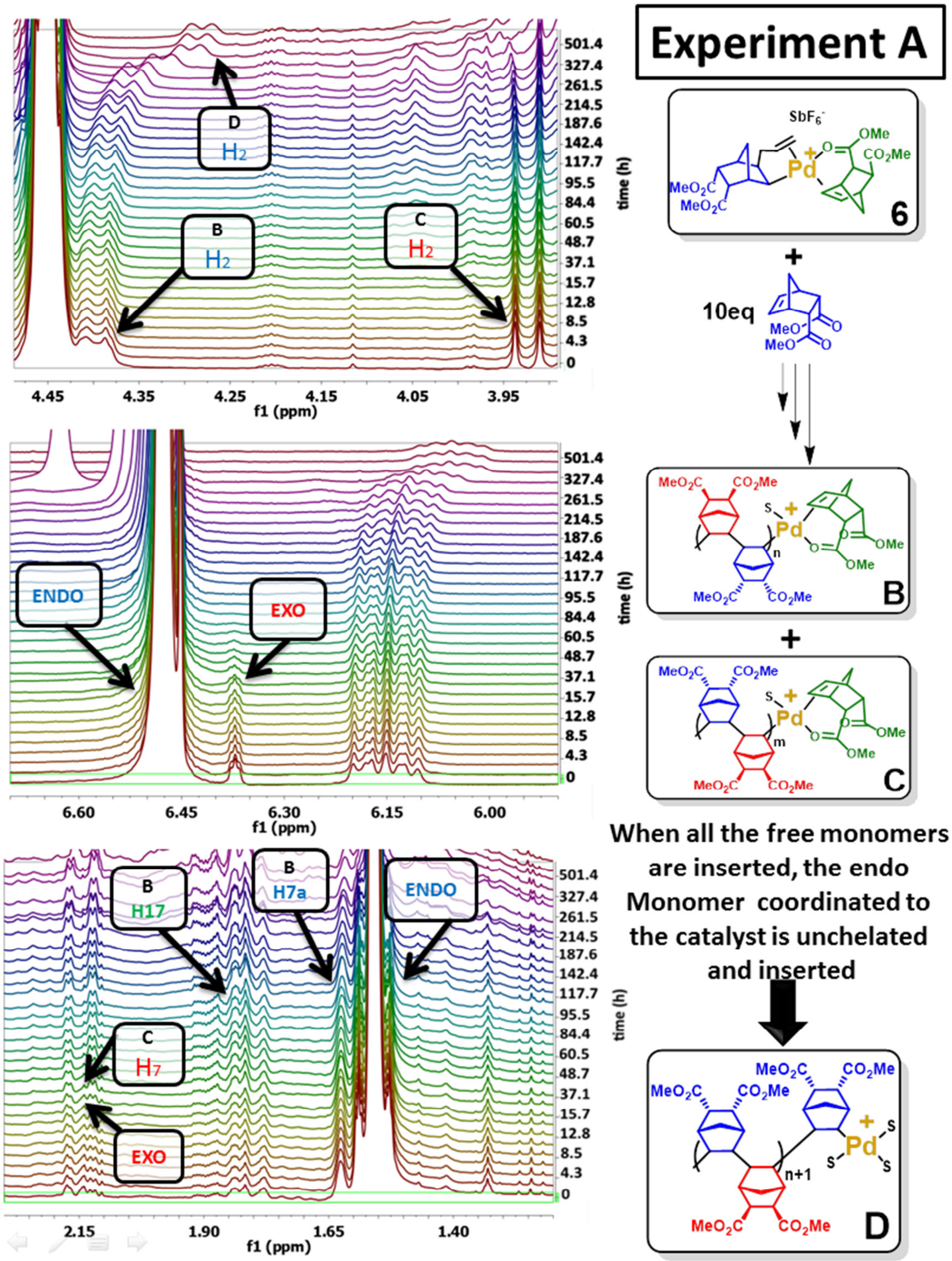

Figure S27. (*Experiment A*) ^{1}H NMR spectra of the polymerization of NBE(CO_2Me)$_2$ (100% endo, 10 eq.) with **6** in CD_3NO_2. Top: region of the H_2 protons, indicating that both *endo* and *exo* monomers are inserted. At the end of the polymerization, the chelate disappears and the last *endo* monomer is inserted. Center: region of the C=C double bonds, showing that only traces of *exo* monomer are present (less than 1.5% relative to *endo* monomer). Bottom: increase of the curvature of the baseline with time, indicative of the formation of a polymer (very broad resonance).

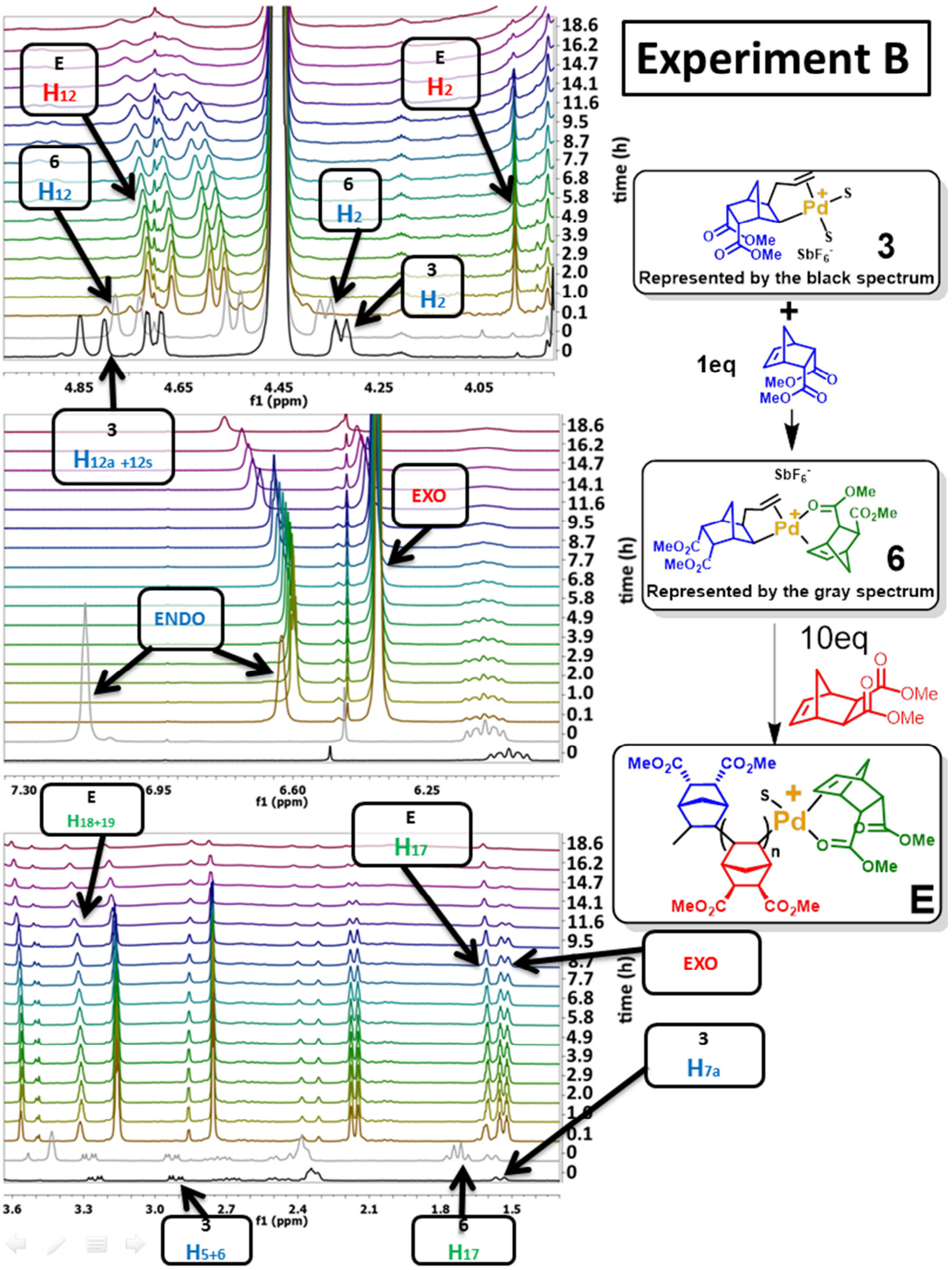

Figure S28. (*Experiment B*) ^{1}H NMR spectra of the polymerization of NBE(CO_2Me)$_2$ (100% *exo*, 10 eq.) with **6** in CD_3NO_2. Top: region of the H_2 protons. Center: region of the C=C double bonds. Bottom: region of the aliphatic protons.

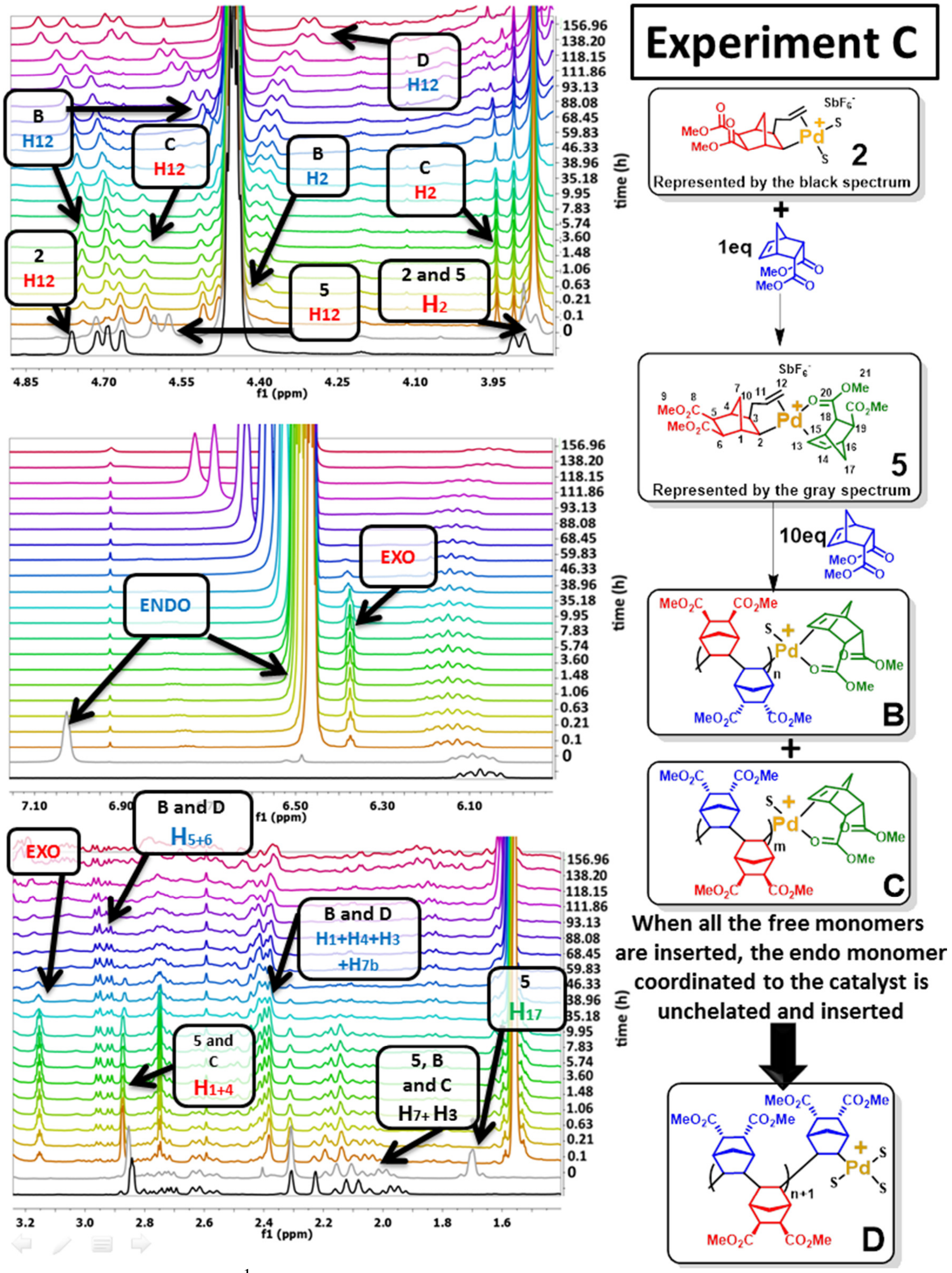

Figure S29. (*Experiment C*) ^{1}H NMR spectrums of NBE(CO_2Me)$_2$ (100% *endo*, 10 eq.) with **5** in CD_3NO_2. Top: region of the H_2 protons, showing that the *endo* H_2 appears gradually. Center: region of the C=C double bonds. Bottom: region of the aliphatic protons.

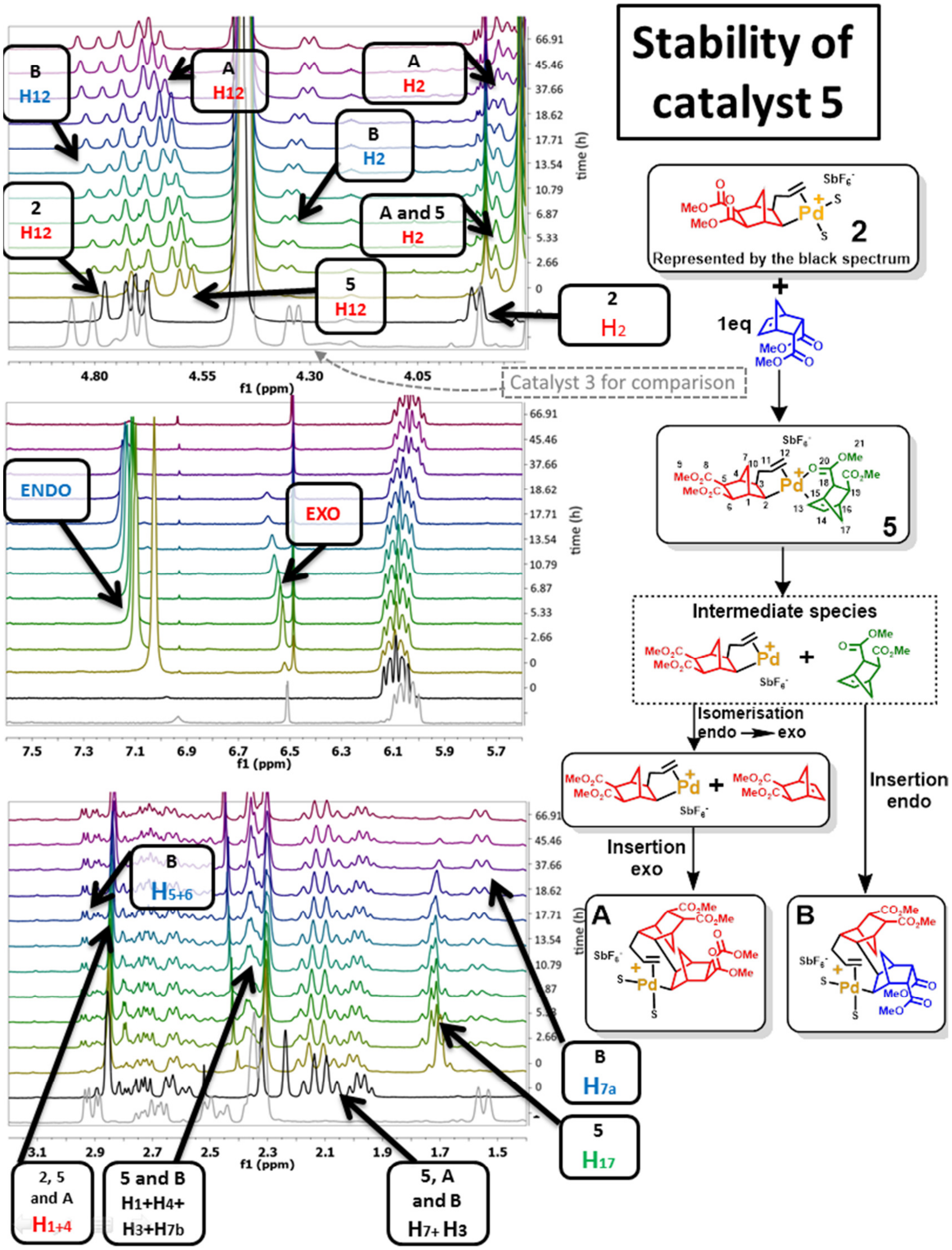

Figure S30. ^{1}H NMR spectra of catalyst **5** vs time. Top: region of the H_{12} protons and H_2, showing that the *endo* H_2 and H_{12} gradually appear to the expense of the exo H_2 and H_{12}. Center: region of the C=C double bonds, showing the rectification of *endo* monomer and the decrease of both monomers concentration. Bottom: region of the aliphatic protons.

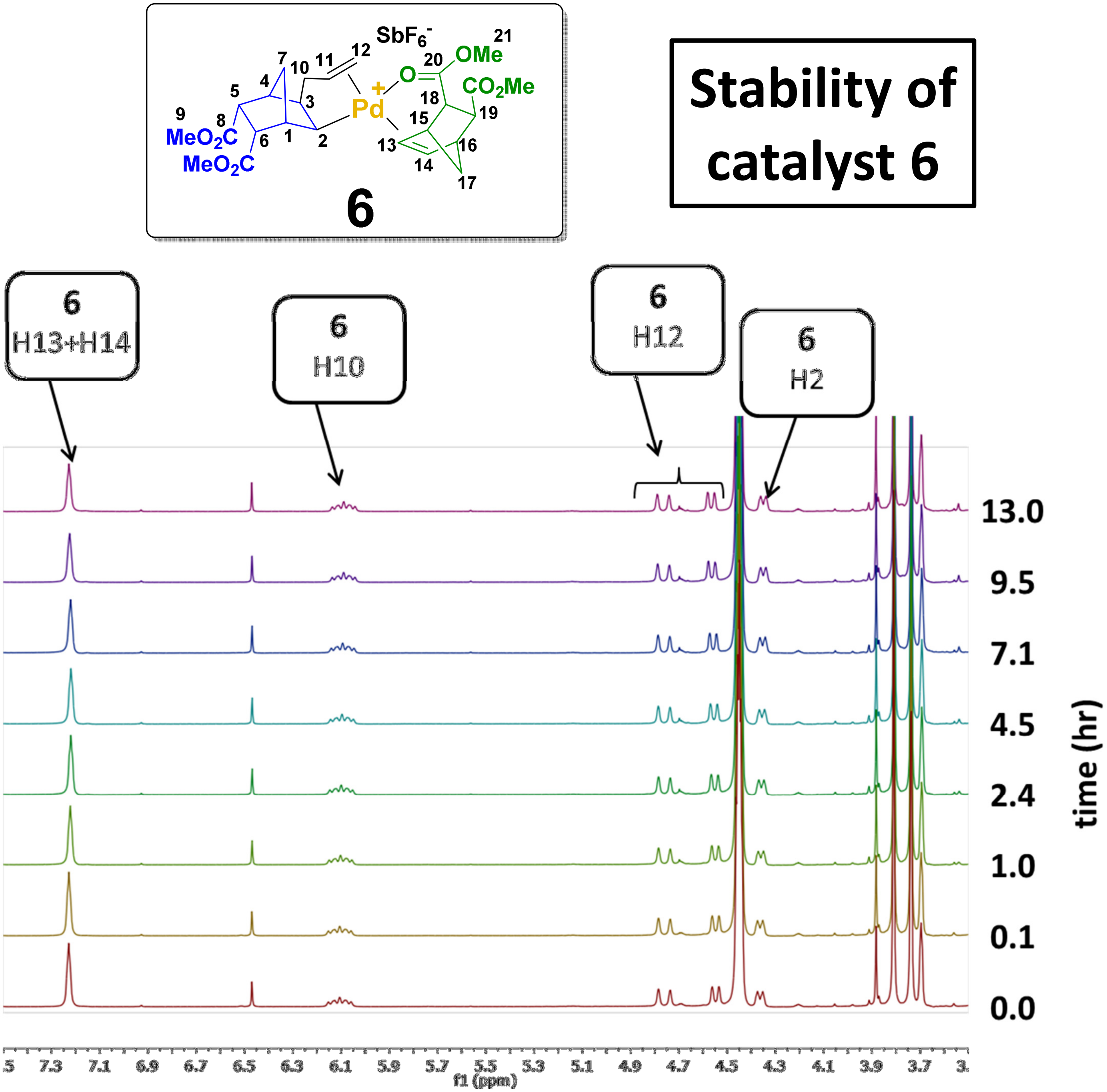

Figure S31. ^{1}H NMR of catalyst **6** over time, indicating the high stability of the chelate

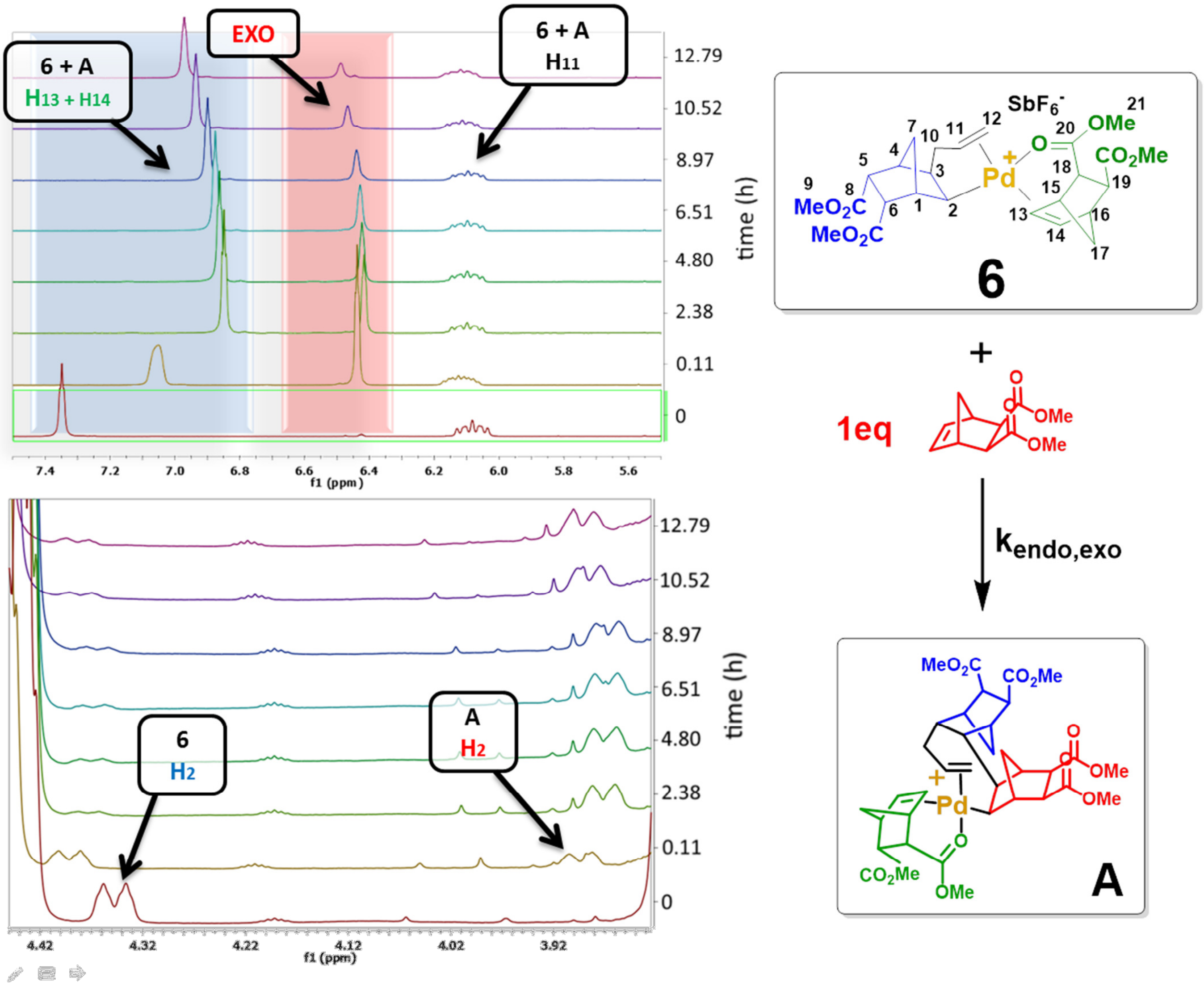

Figure S32. ^{1}H NMR spectra of the polymerization of NBE(CO_2Me)$_2$ (100% exo, 1 eq.) with **6** in CD_3NO_2. Top: vinyl region, showing that the exo monomer is inserted, but the endo chelate is conserved. Bottom. Rapid disappearance of the H_2 proton in 6 to the benefit of H_2 proton in A, indicative of the high value of $k_{endo,exo}$.

A

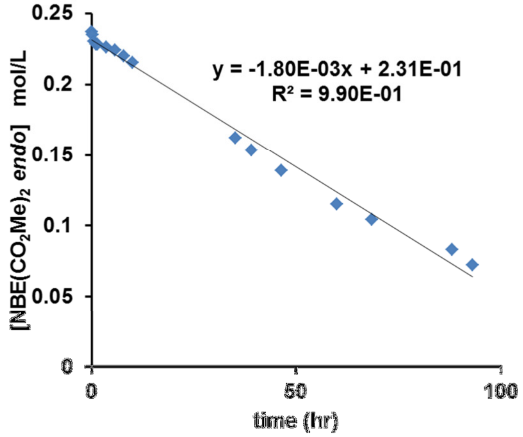

B

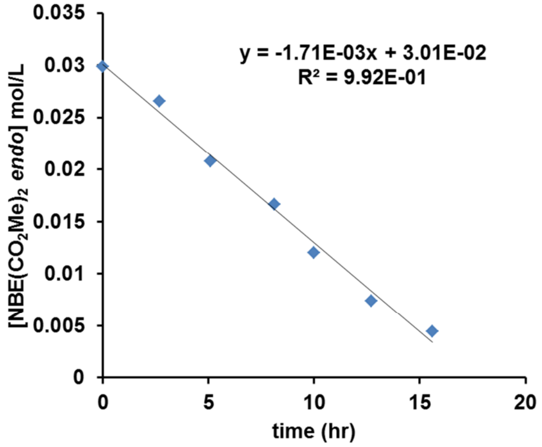

C

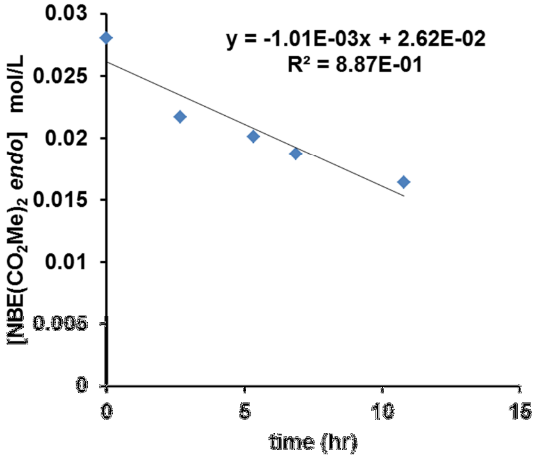

Figure S33. Kinetics plots for the reaction of **5** with *endo*-NBE(CO_2Me)$_2$. A. [**5**] = 0.037 mol/L, [*endo*-NBE(CO_2Me)$_2$]/[**5**] = 7, B. [**5**] = 0.041 mol/L, [*endo*-NBE(CO_2Me)$_2$]/[1] = 0.75 C. [**5**] = 0.031 mol/L, [*endo*-NBE(CO_2Me)$_2$]/[**5**] = 1. The value of $k_{exo,endo}$ was determined by taking the ratio of the slope to the catalyst concentration.

A

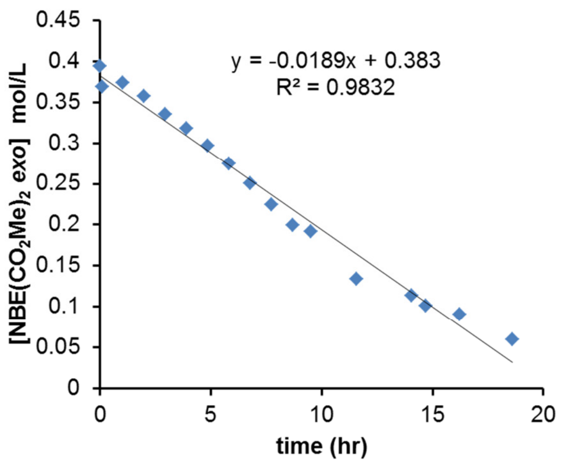

B

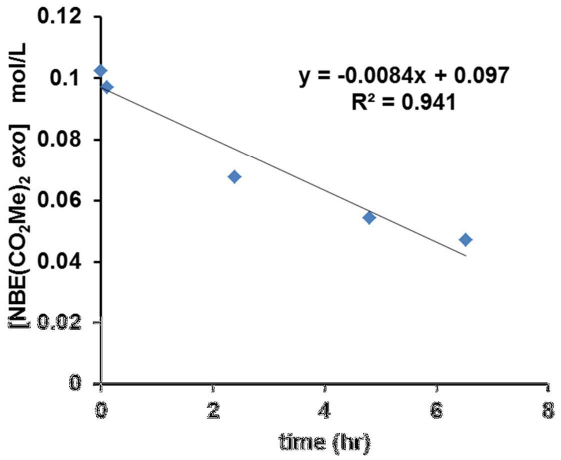

<u>Figure S34.</u> Kinetics plots for the reaction of **5** with *exo*-NBE$(CO_2Me)_2$. A. [**5**] = 0.038 mol/L, [NBE$(CO_2Me)_2$]/[**5**] = 10, B. [**5**] = 0.030 mol/L, [*exo*-NBE$(CO_2Me)_2$]/[**5**] = 3 The value of $k_{exo,exo}$ was determined by taking the ratio of the slope to the catalyst concentration.

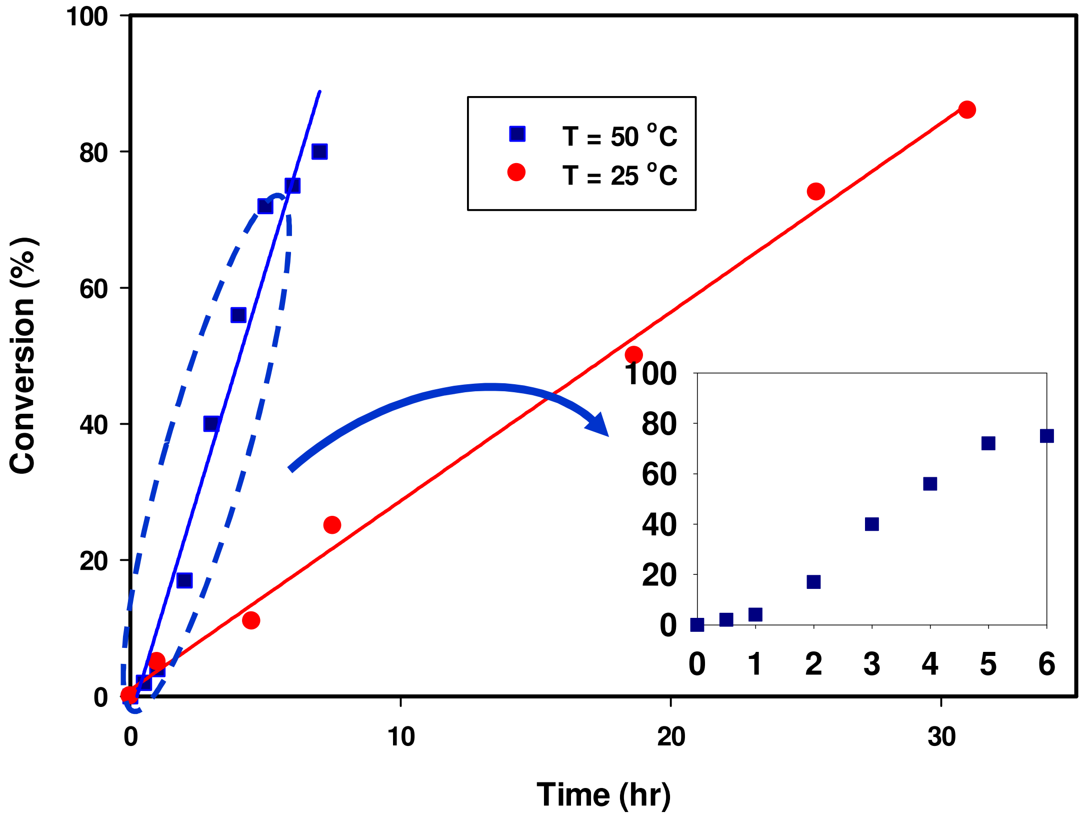

Figure S35. Kinetic plot for the polymerization of NBE(CO_2Me) (73% endo) at 25 °C and 50 °C by **1** in nitromethane ([NBE(CO_2Me)]/[**1**] = 200, [NBE($CO_2Me)_2$] = 0.47 mol/L). The lines correspond to linear fits. The inset corresponds to a zoom of the kinetics at early times (induction period).

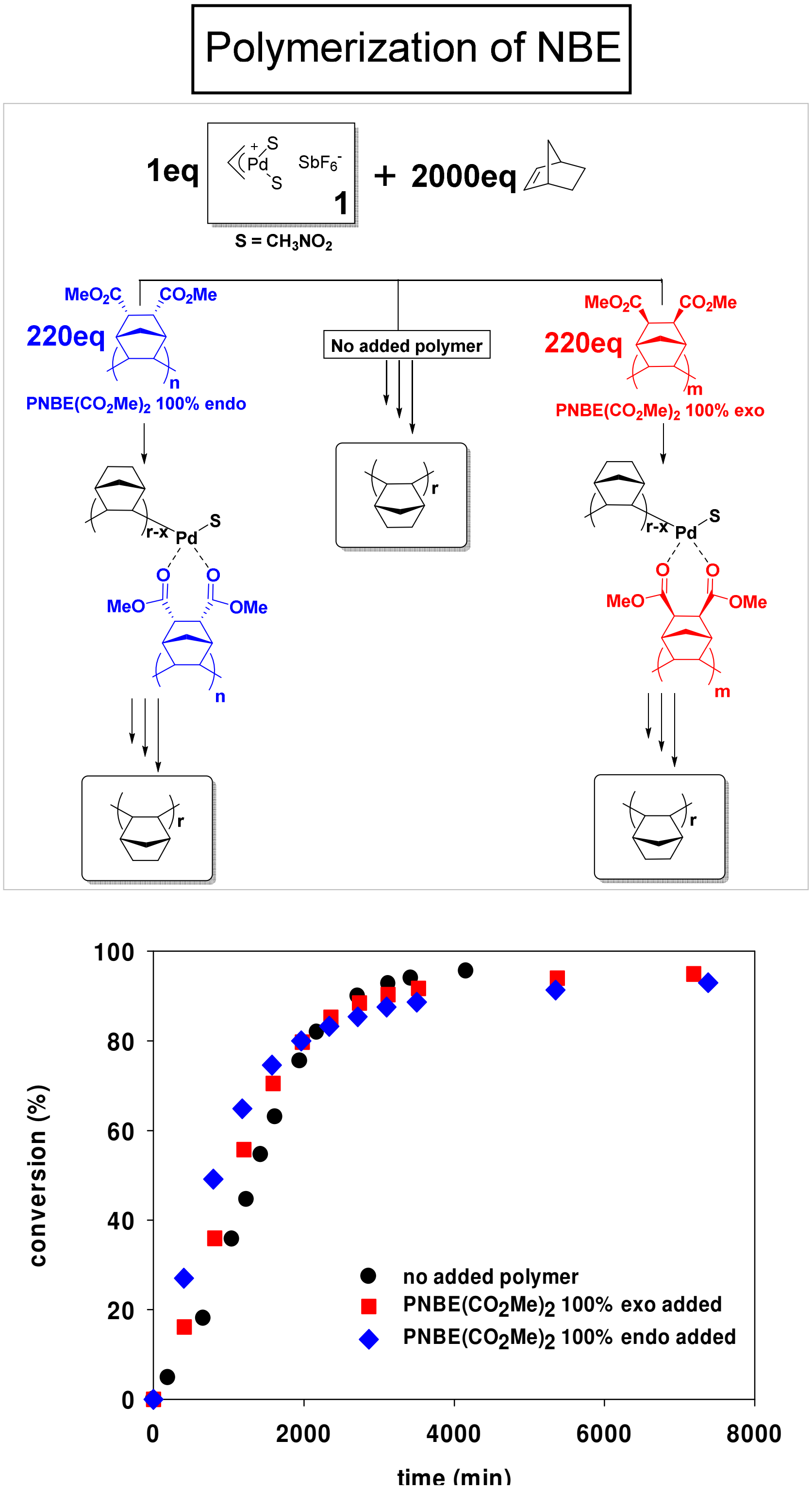

Figure S36. Kinetic plot for the polymerization of NBE at 25 °C by **1** in the absence of added polymer, and in the presence of PNBE(CO_2Me)$_2$ (100% exo or 100% endo). Experimental conditions T = 25 °C in in deuterated tetrachloroethane, [NBE] = 0.22 mol/L, [NBE]0/[1] = 2000, when added PNBE(CO_2Me)$_2$ is present [C=O] /[**1**] = 220)

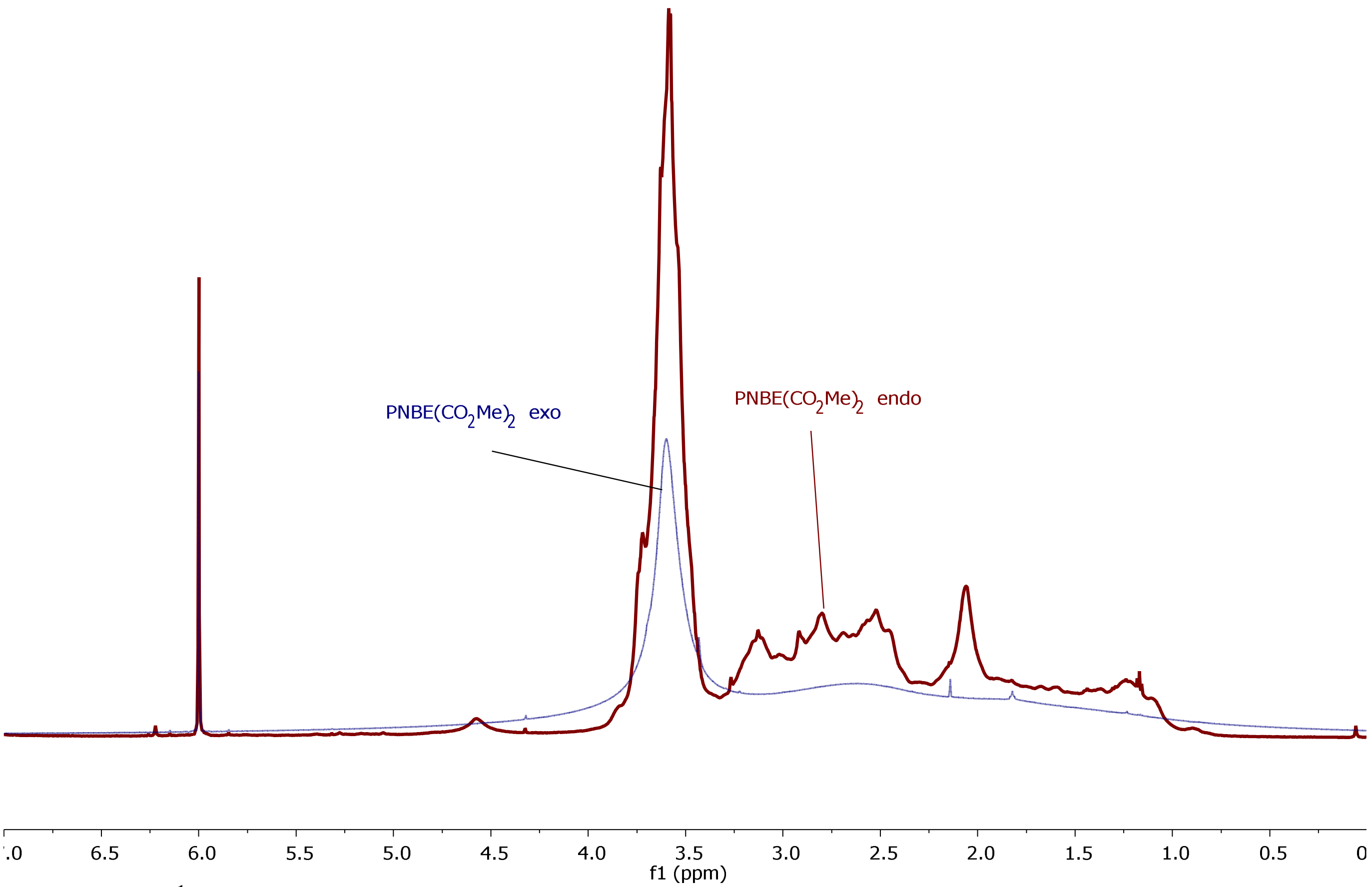

Figure S37. ^{1}H NMR of PNBE(CO_2Me)$_2$ prepared with the *exo* monomer and PNBE(CO_2Me)$_2$ prepared with the *endo* monome

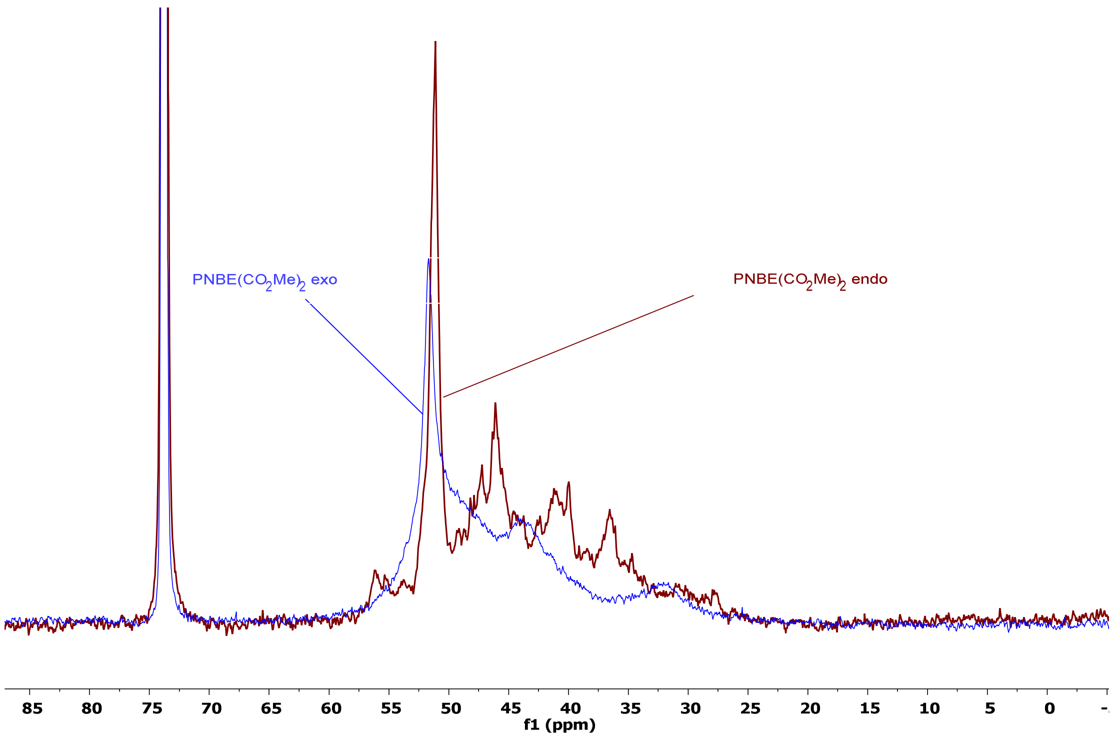

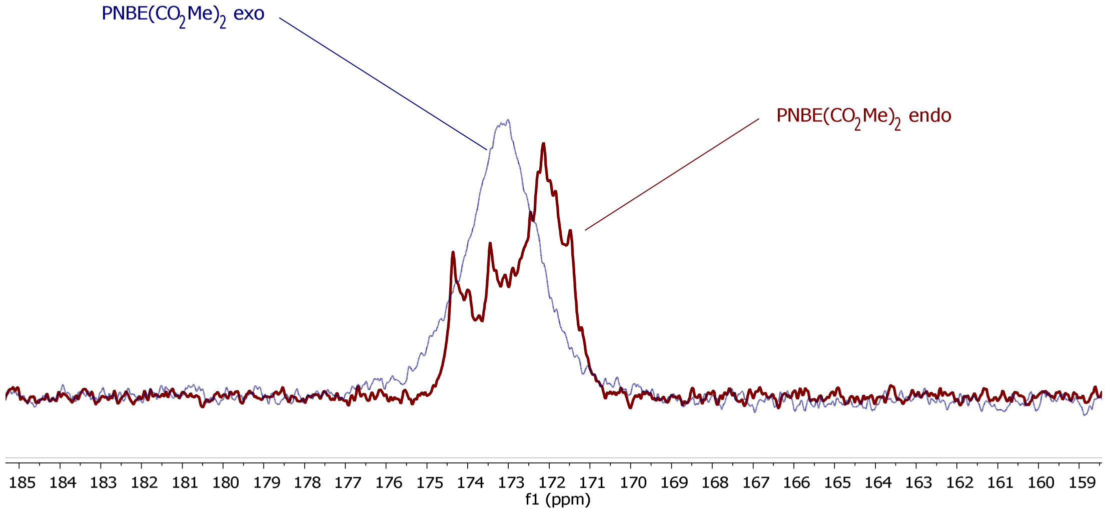

Figure S38. $^{13}C\{^{1}H\}$ NMR of PNBE(CO_2Me)$_2$ prepared with the *exo* monomer and PNBE(CO_2Me)$_2$ prepared with the *endo* monomer (top) and zoom of the carbonyl region (bottom)

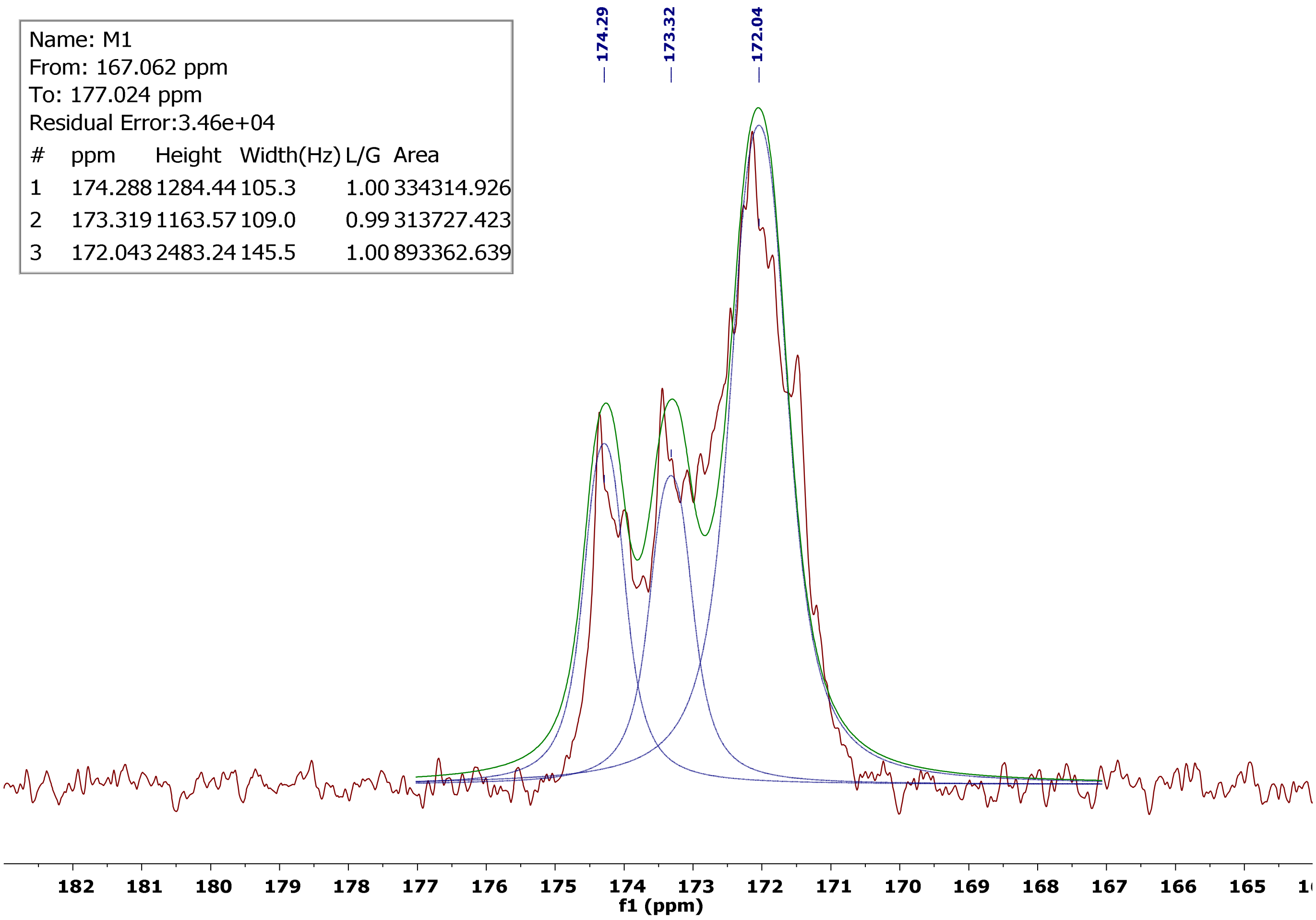

<u>Figure S39.</u> Zoom of the carbonyl region of the ^{13}C spectrum PNBE$(CO_2Me)_2$ prepared with the endo monomer and superimposed deconvolution by three peaks.

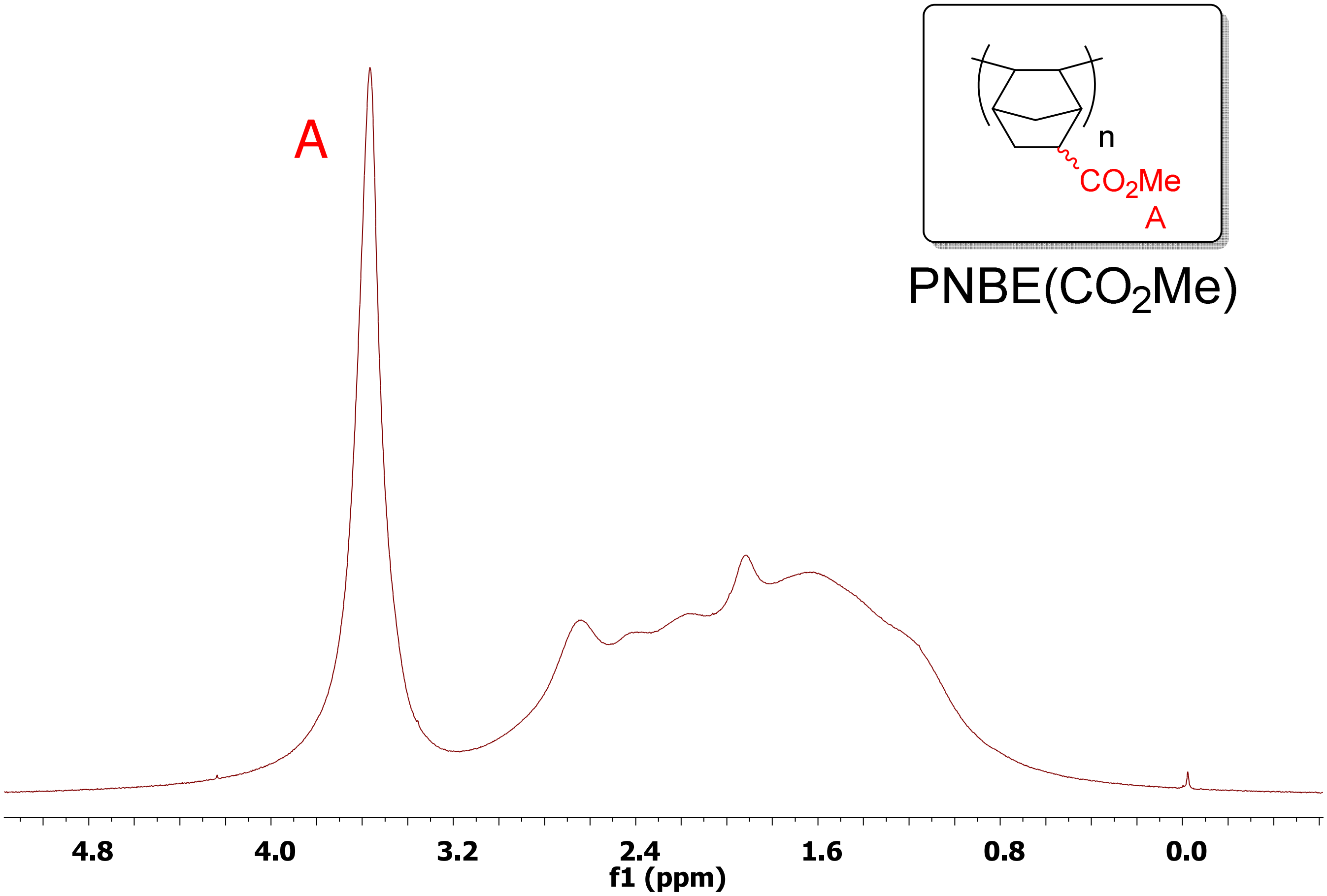

Figure S40. ^{1}H spectrum PNBE(CO_2Me) (catalyst loading = 0.5 mol%, prepared with NBE(CO_2Me) 75% *endo,* solvent 1,1,2,2-Tetrachloroethane-d2).

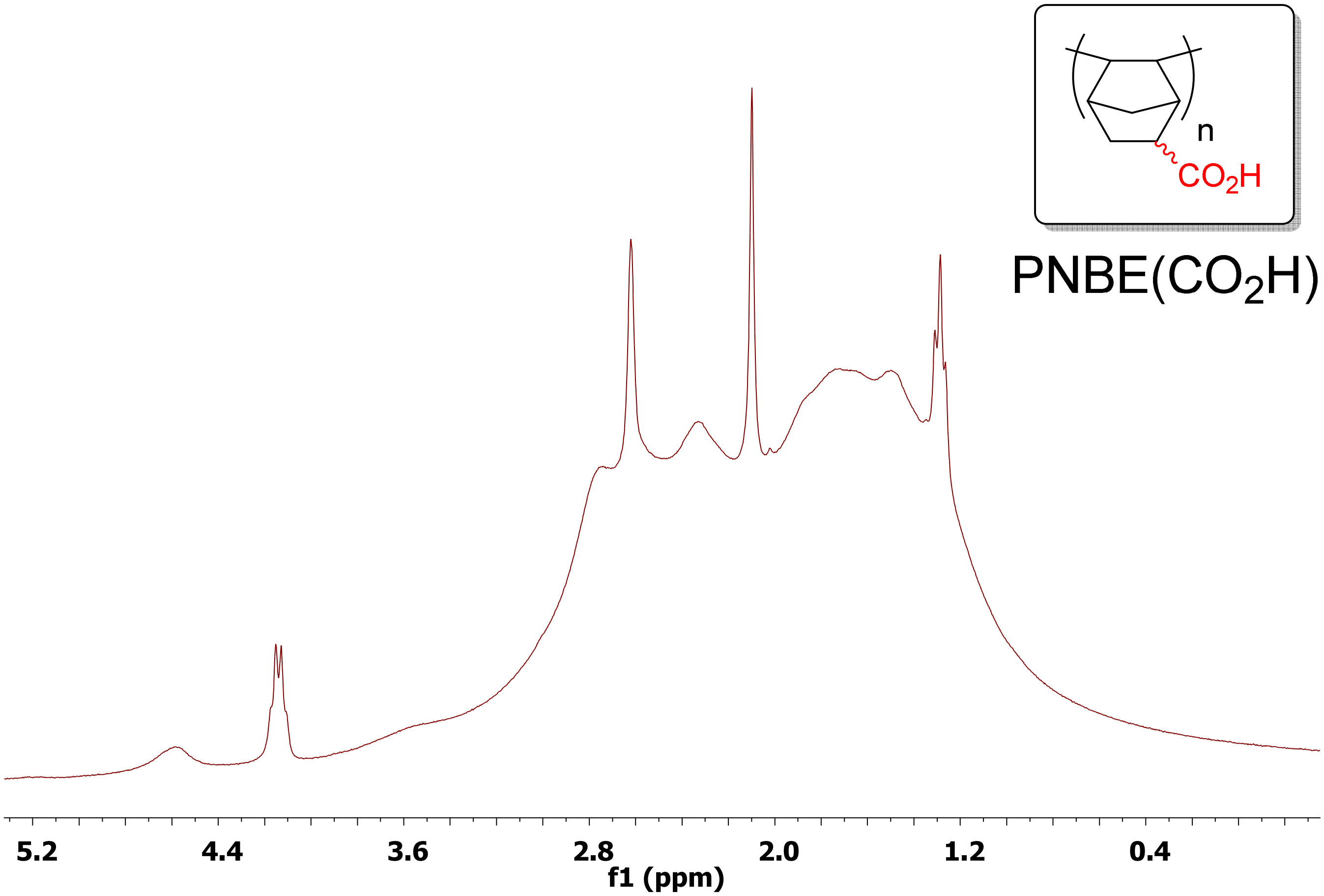

Figure S41. ^{1}H spectrum PNBE(CO_2H) (catalyst loading = 0.01 mol%, prepared with NBE(CO_2H) 75% *endo,* solvent DMSO-d6).

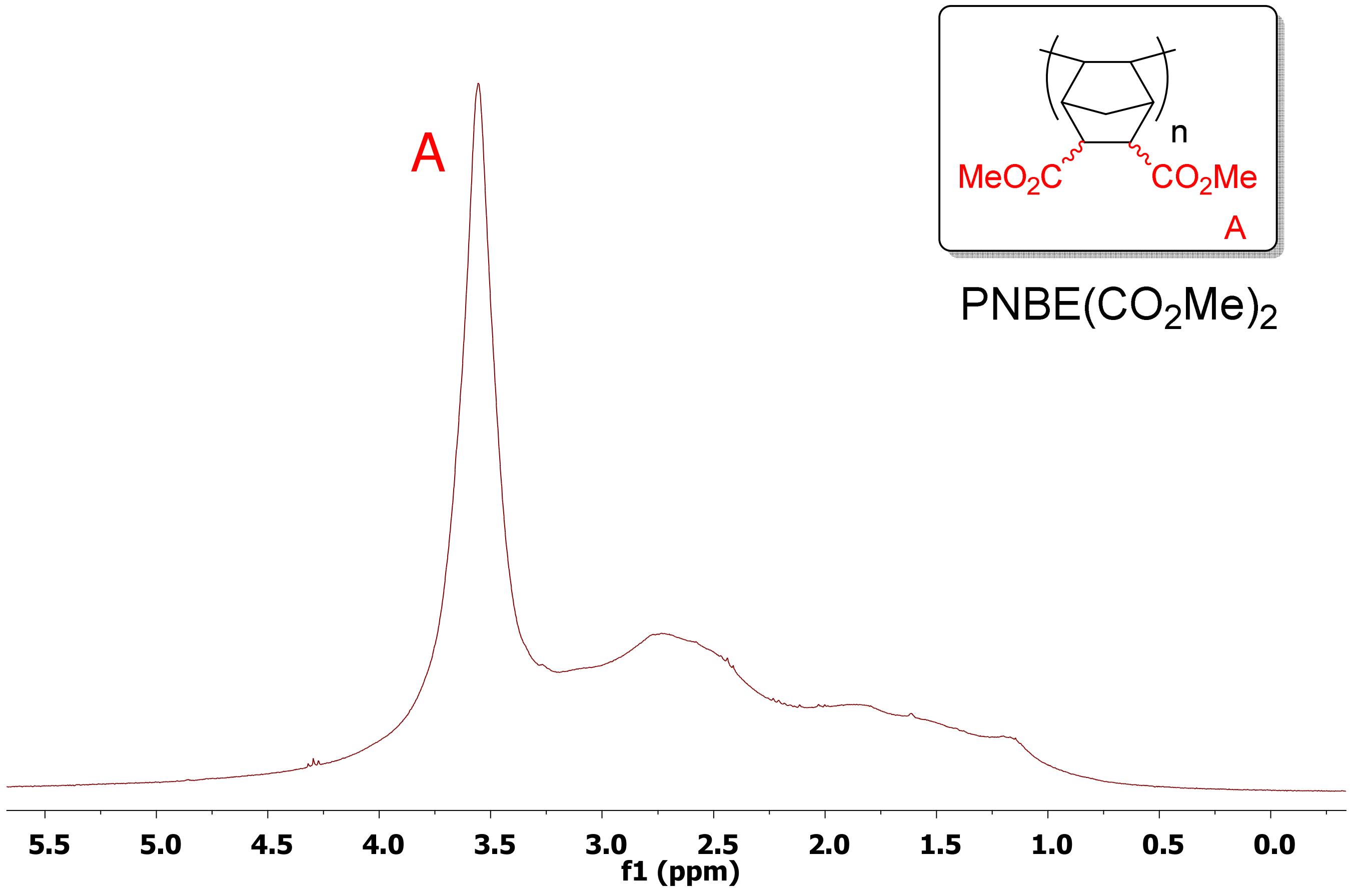

Figure S42. ^{1}H spectrum PNBE$(CO_2Me)_2$ (catalyst loading = 0.01 mol%, prepared with NBE$(CO_2Me)_2$ 75% *endo,* solvent chloroform-d).

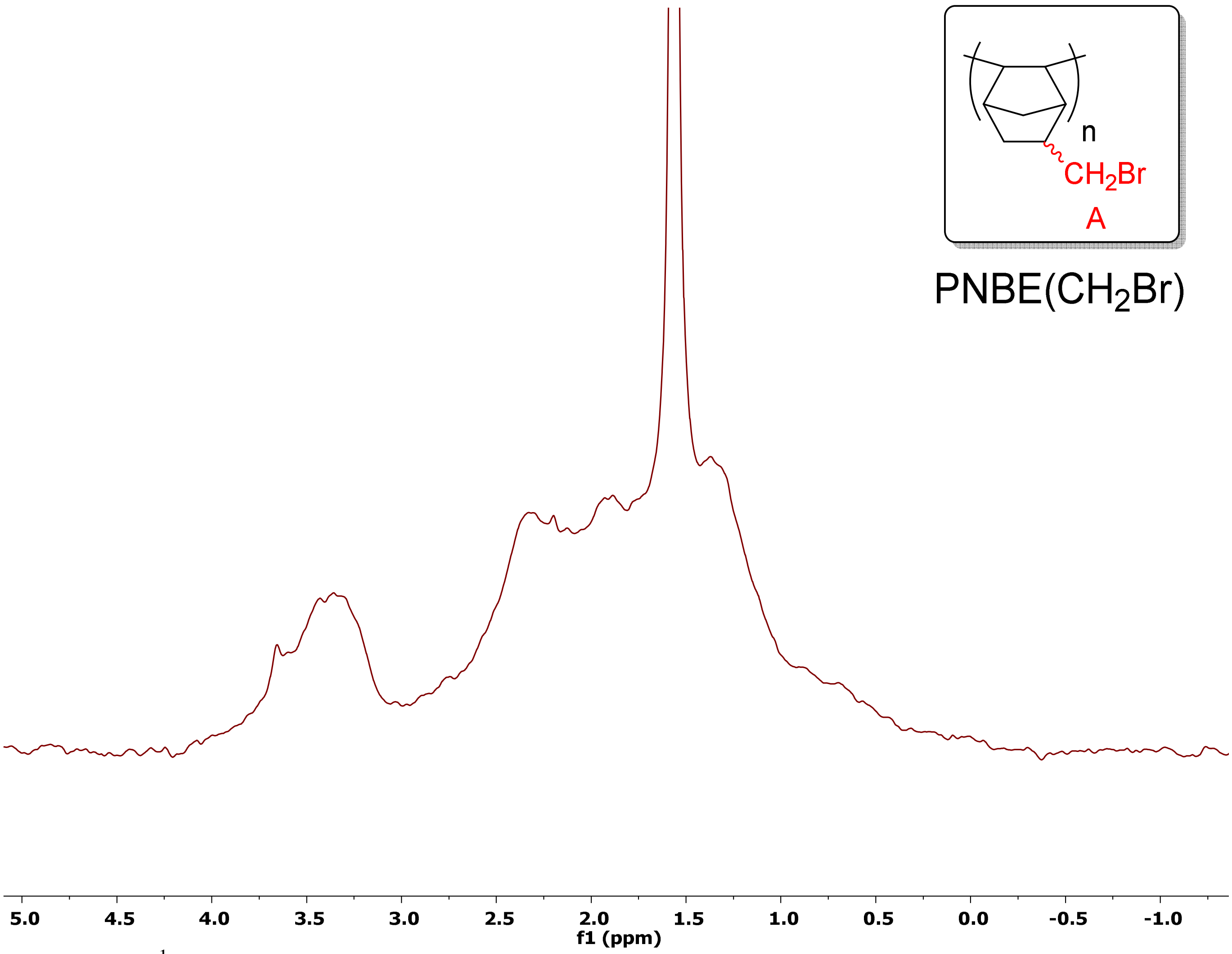

Figure S43. ^{1}H spectrum PNBECH$_2$Br (catalyst loading = 0.2 mol%, prepared with NBECH$_2$Br 86% *endo,* solvent CDCl$_3$)

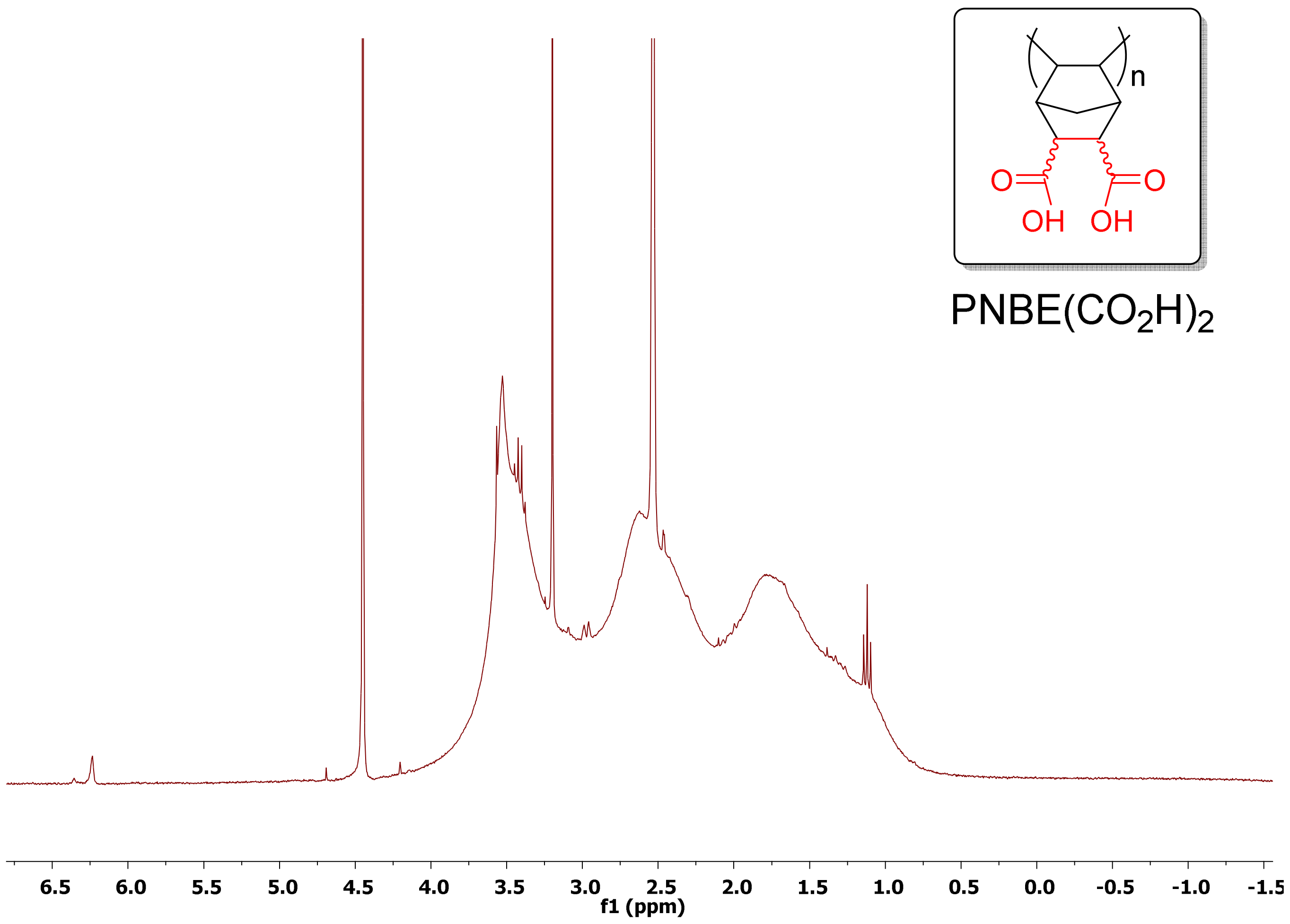

Figure S44. ^{1}H spectrum PNBE(COOH)$_2$ (catalyst loading = 1 mol%, prepared with NBE(COOH)$_2$ *exo*, solvent D_2O)

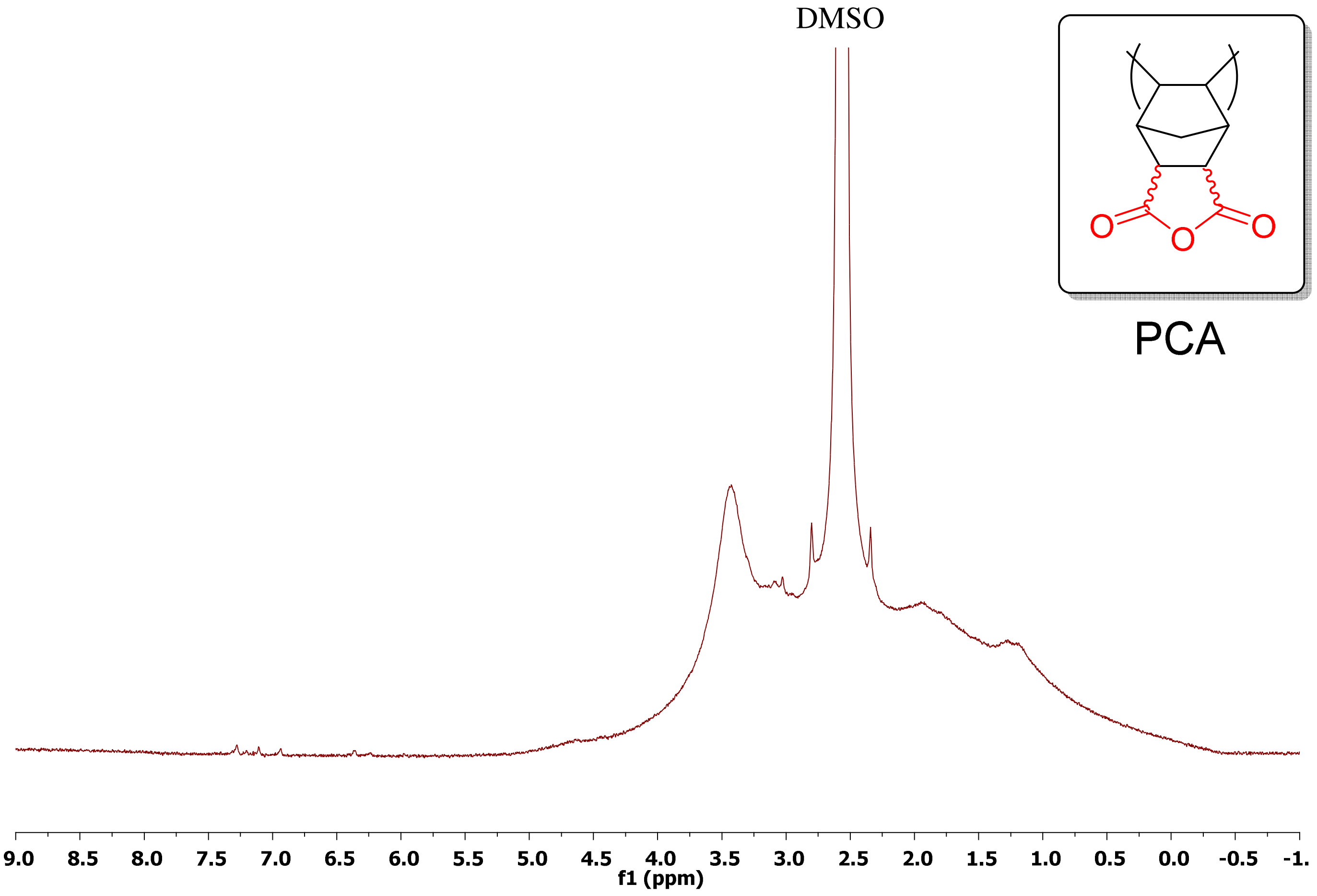

<u>Figure S45</u>. ^{1}H spectrum of PCA (catalyst loading = 0.5 mol%, prepared with CA *exo,* solvent DMSO d6)

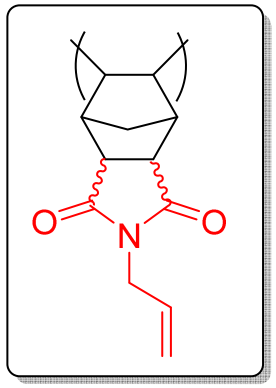

PNBE imide

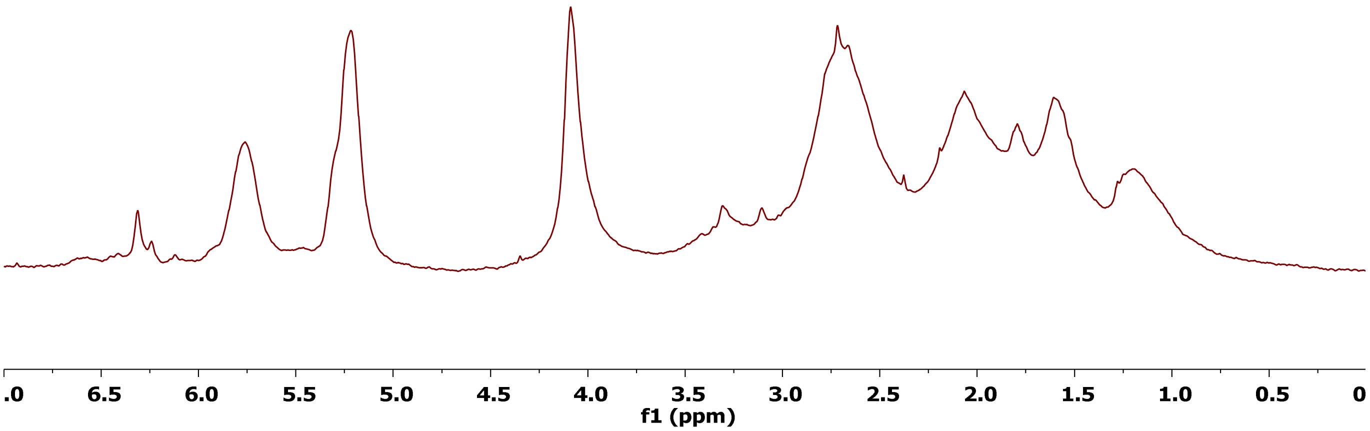

<u>Figure S46</u>. ^{1}H spectrum of PNBE(imide) (catalyst loading = 0.1 mol%, prepared with NBE imide 90% *exo,* solvent $CDCl_3$)

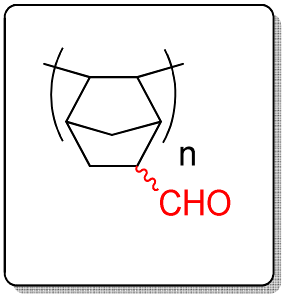

PNBE(CHO)

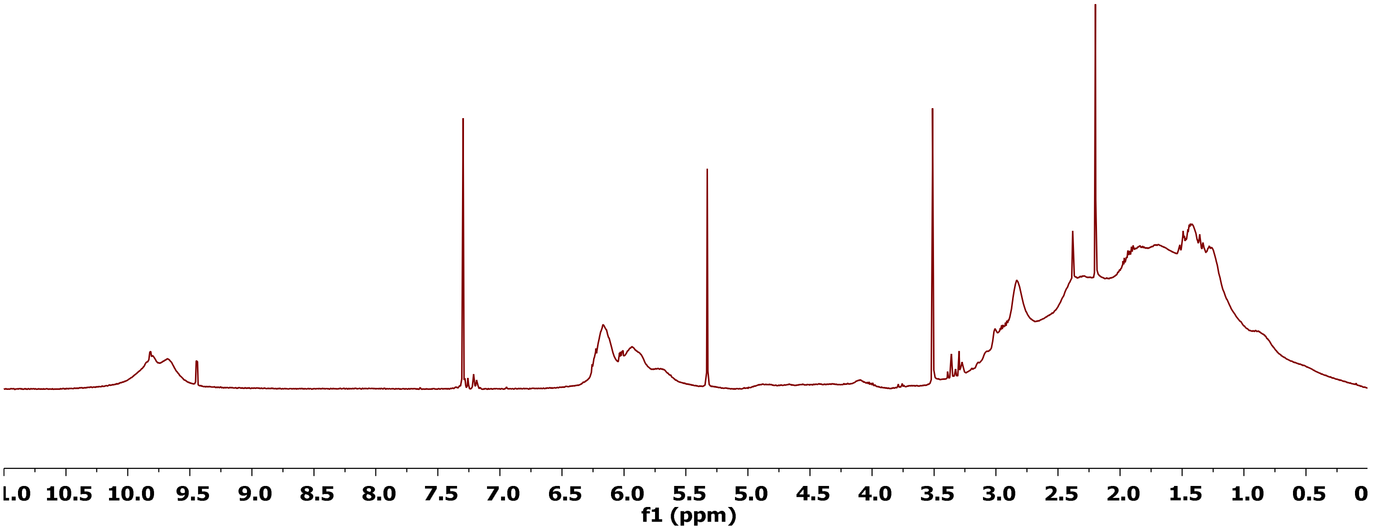

<u>Figure S47</u> ^{1}H spectrum PNBE(CHO) (catalyst loading = 0.05 mol%, prepared with NBE(CHO) 80% *endo,* solvent $CDCl_3$)

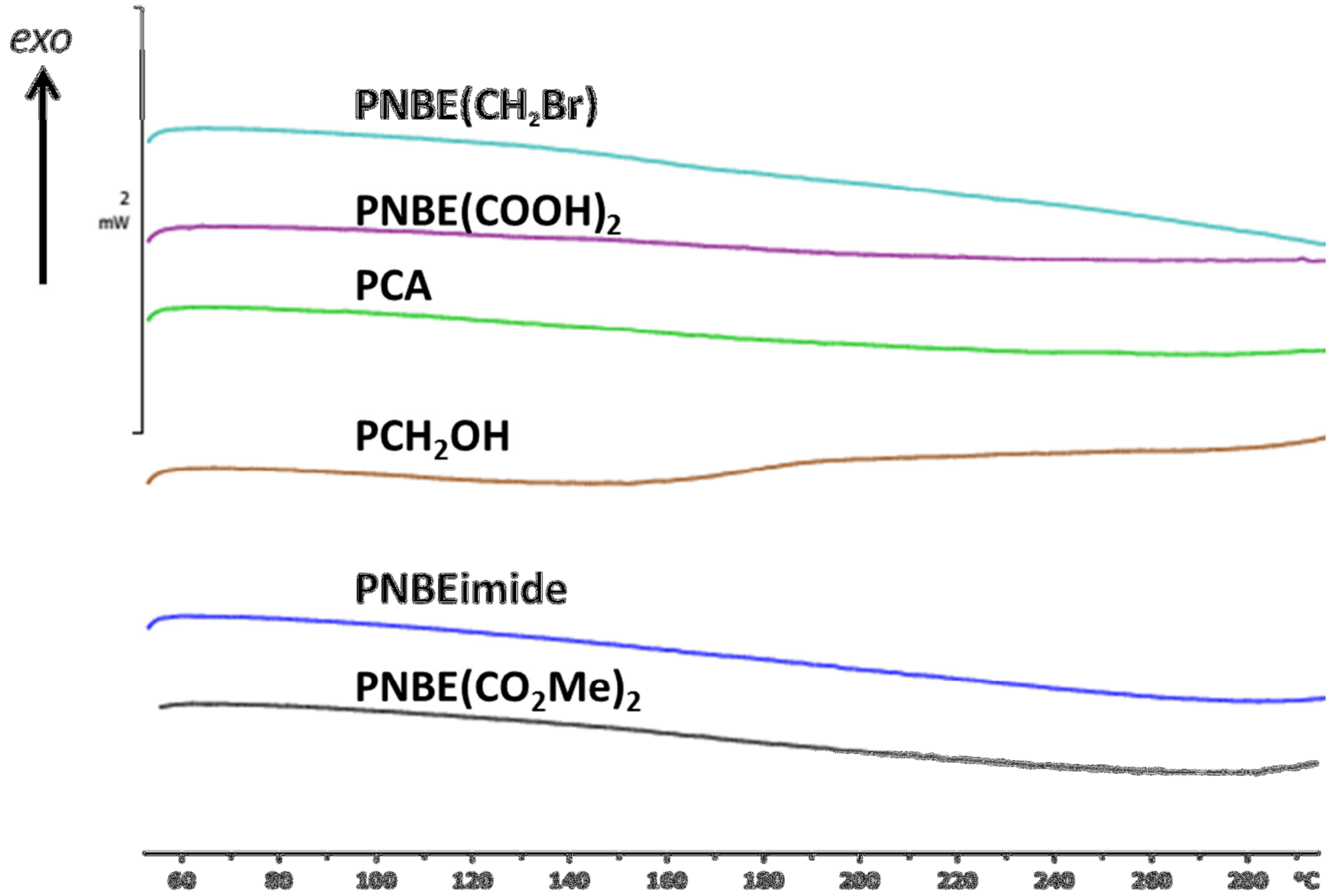

Figure S48 DSC thermograms of the polymers (second heating ramp 50 - 300 ^{o}C), showing the absence of Tg.

The exothermic transition at 160 ^{o}C for PCH_2OH is not associated with a Tg, as a Tg is expected to be endothermic and the corresponding cooling curve does not show any thermal transition. Putatively, this thermal transition is assigned to an irreversible dehydration reaction. The polymer PNBE(CHO) is not sufficiently stable to be heated up to 300 ^{o}C.

Table S1. Characteristic bond lengths and bond angles for structures **2-H_2O, 3-THF**, **2-s**, **3-s** and **4-s** (for the sake of simplicity, the atom numbering used in this table corresponds to the attached scheme, and not to the atom numbering in the X-Ray structures)

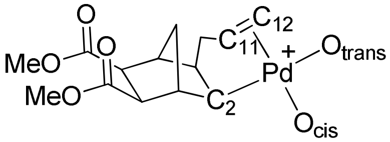

Structures	2-H2O	3-THF	2-s	3-s	4-s
distances (Å)					
Pd-C2	2.005	2.017	2.010, 2.014<br>2.015, 2.022	2.013	2.022
Pd-C11	2.110	2.141	2.140, 2.147,<br>2.139, 2.176	2.139	2.146
Pd-C12	2.110	2.153	2.146, 2.132,<br>2.129, 2.146	2.133	2.129
Pd-O_{trans}	2.236	2.236	2.235, 2.232,<br>2.201, 2.255	2.204	2.249
Pd-O_{cis}	2.125	2.103	2.132, 2.130,<br>2.143, 2.125	2.129	2.143
C=O	1.193<br>1.230	1.196<br>1.215	1.237, 1.228,<br>1.243, 1.218,<br>1.232, 1.223<br>1.221, 1.241	1.226,<br>1.220	1.221,<br>1.229
angles (^{o})					
C2-Pd-O_{cis}	94.46	94.35	96.1, 97.83,<br>93.47, 95.93	94.01	90.65
C2-Pd-O_{trans}	179.93	175.73	176.01, 178.97,<br>174.18, 177.96	175.86	173.91

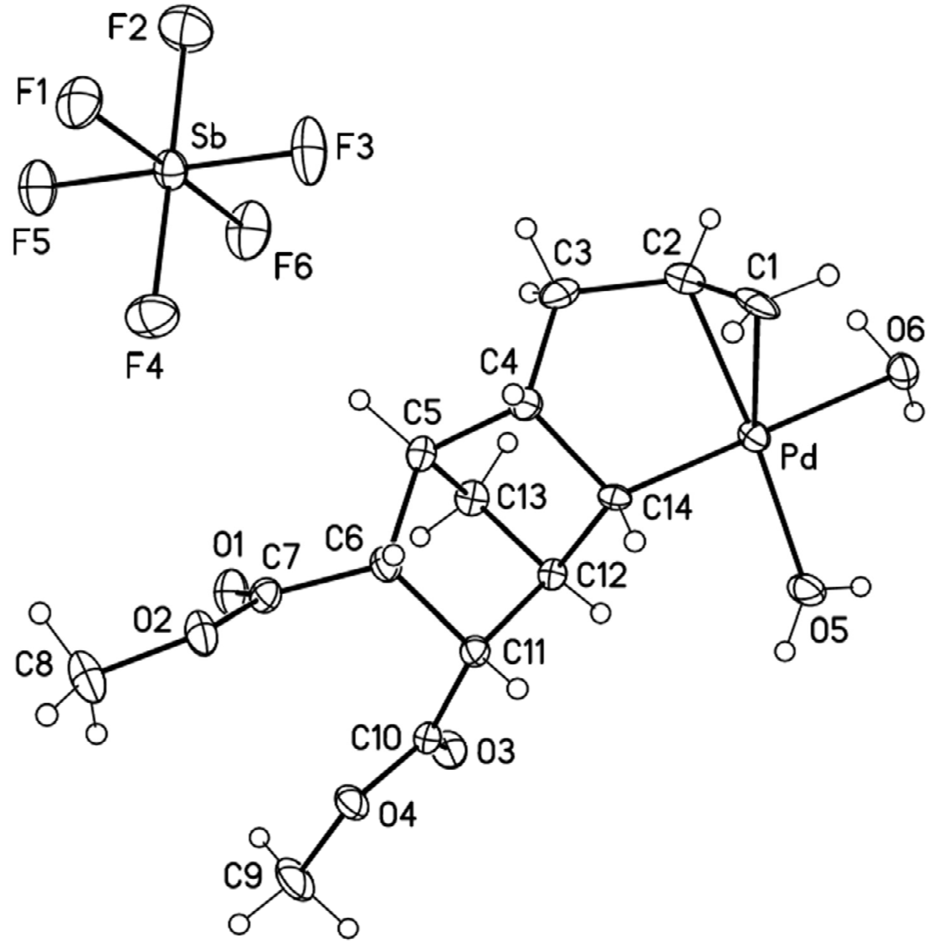

<u>Figure S49</u> ORTEP view of **2-H_2O** with 50% probability ellipsoids

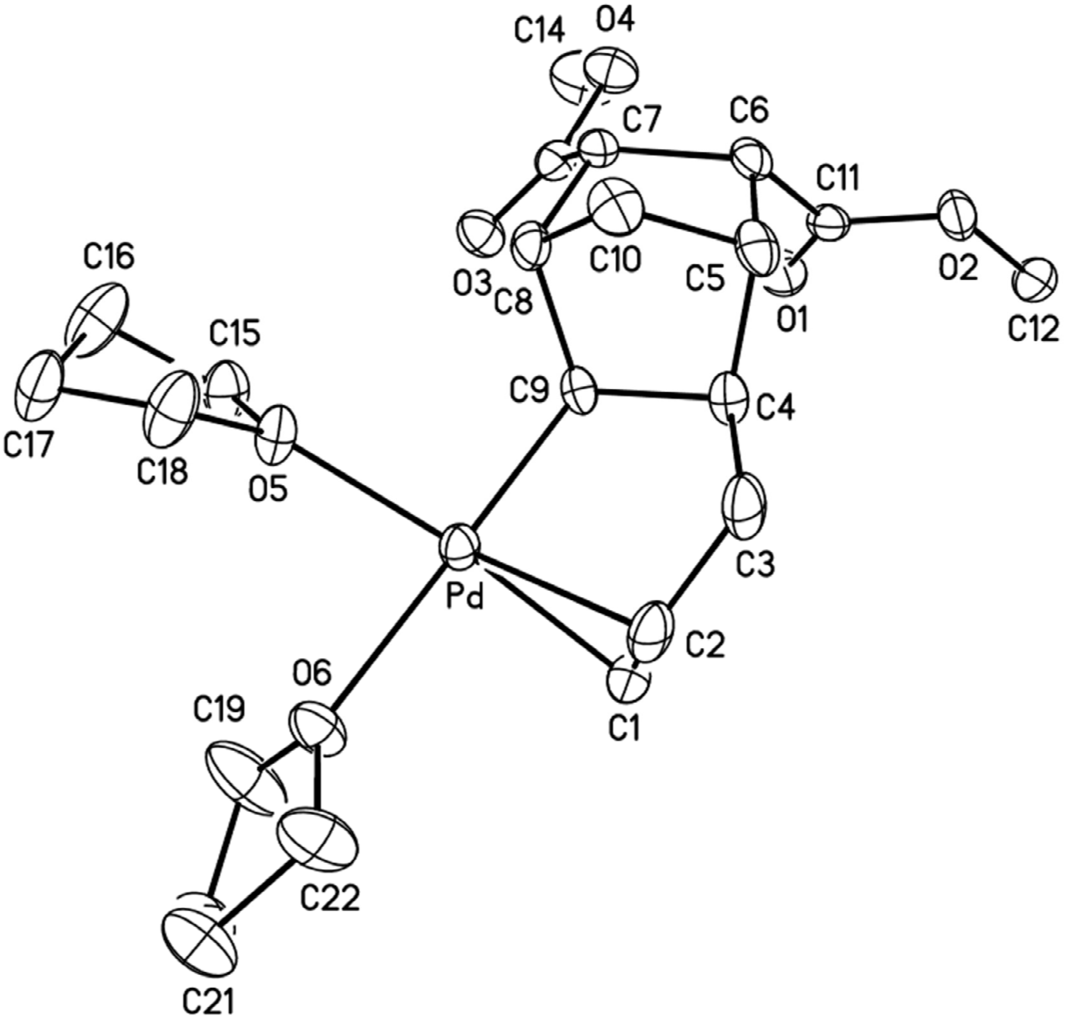

Figure S50. ORTEP view of **3-THF** with 50% probability ellipsoids (for the sake of clarity, the SbF_6 anion has been omitted)

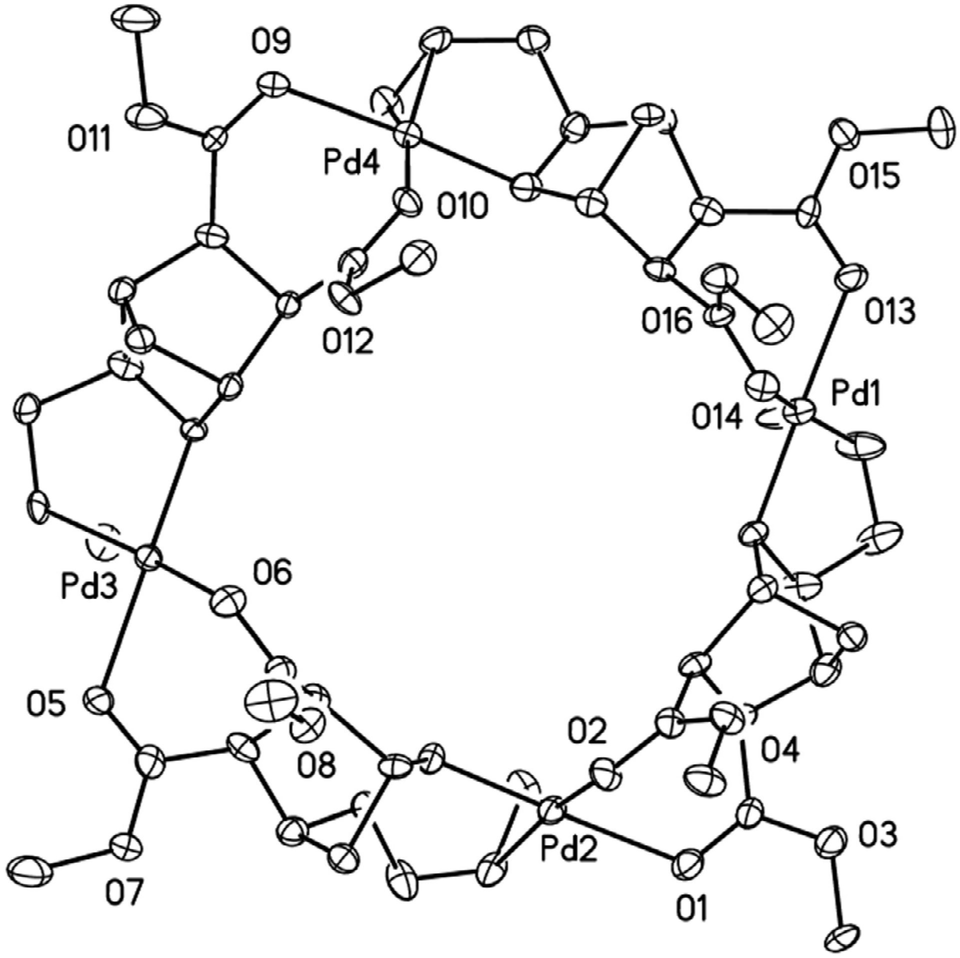

Figure S51. ORTEP view of **2s** with 50% probability ellipsoids (for the sake of clarity, the four SbF_6 anions and solvent molecules have been omitted)

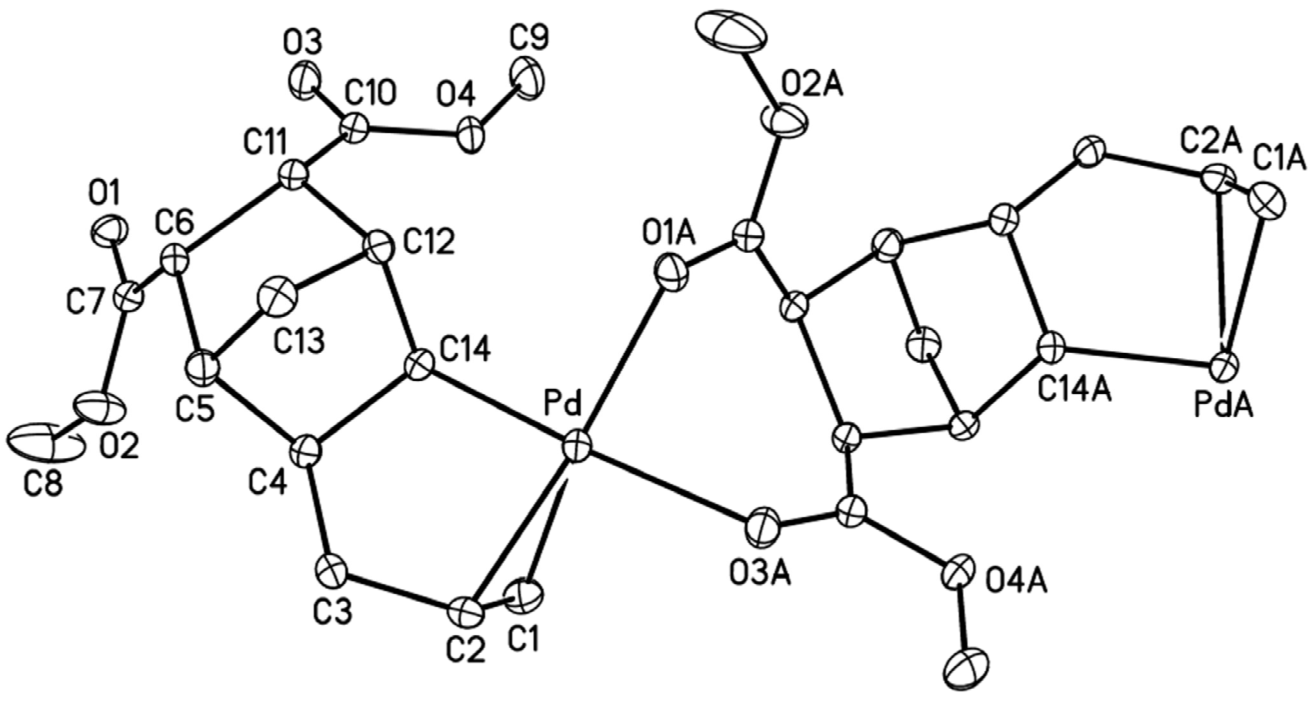

Figure S52. ORTEP view of **3s** with 50% probability ellipsoids (for the sake of clarity, the SbF_6 anion and solvent molecules have been omitted)

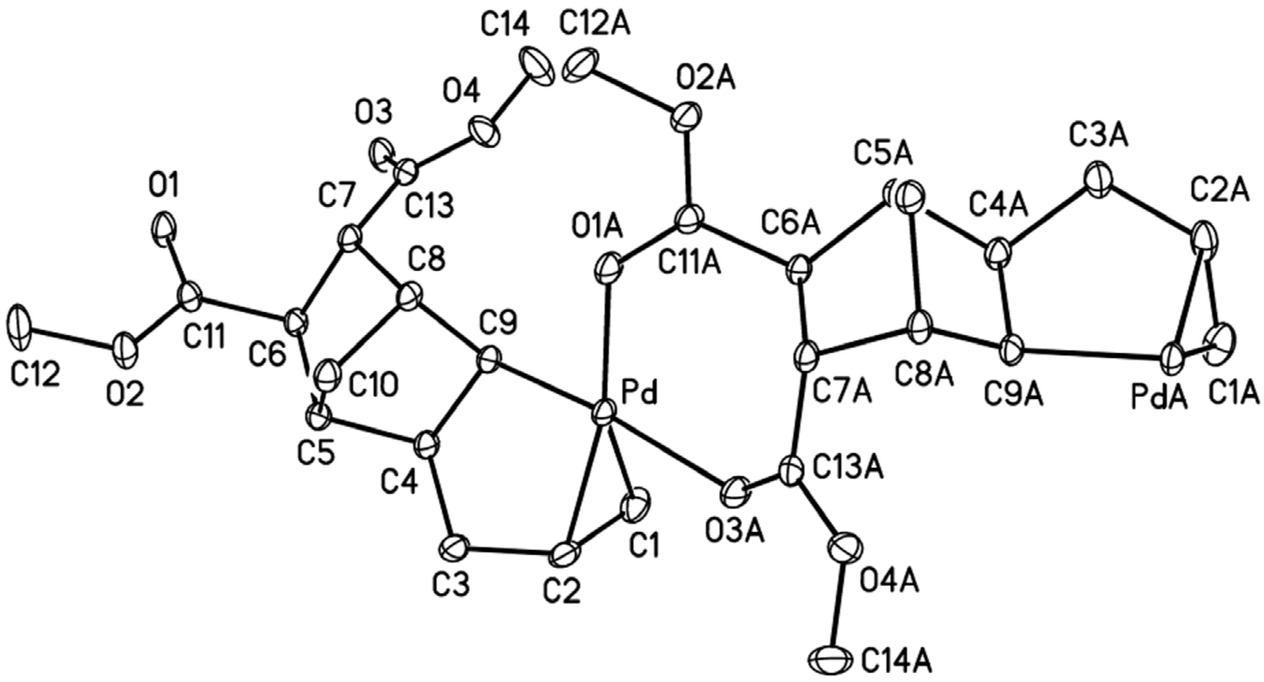

<u>Figure S53.</u> ORTEP view of **4Ns** with 50% probability ellipsoids (for the sake of clarity, the SbF_6 anion and solvent molecules have been omitted)

References

(1) Roberts, J. D.; Trumbull, E. R.; Bennett, W.; Armstrong, R. *J. Am. Chem. Soc.* **1950**, *72*, 3116.
(2) Antonović, D. G.; Nikolić, A. D.; Petrović, S. D. *J. Mol. Struct.* **1988**, *174*, 255.
(3) Windmon, N.; Dragojlovic, V. *Green Chem. Lett. Rev.* **2008**, *1*, 155.
(4) Shin, B.-G.; Jang, M.-S.; Yoon, D. Y.; Heitz, W. *Macromol. Rapid Commun.* **2004**, *25*, 728.
(5) Craig, D. *J. Am. Chem. Soc.* **1951**, *73*, 4889.
(6) Kim, S. Y.; Kim, E. K.; Park, M. S.; Cho, Y. I.; Chun, S. H.; Lee, H. J.; Kim, H. Retardation film, a method for preparing retardation film and polarizer comprising the retardation film, 2010.
(7) Dolman, S. J.; Hultzsch, K. C.; Pezet, F.; Teng, X.; Hoveyda, A. H.; Schrock, R. R. *J. Am. Chem. Soc.* **2004**, *126*, 10945.
(8) Frisch, M. J. et al. Gaussian 09, Revision A.02. *Gaussian 09, Revision A.02*, 2009.
(9) Becke, A. D. *J. Chem. Phys.* **1993**, *98*, 5648.
(10) Lee, C.; Yang, W.; Parr, R. G. *Phys. Rev. B* **1988**, *37*, 785.
